# Increased Atmospheric SO_2_ Detected from Changes in Leaf Physiognomy across the Triassic–Jurassic Boundary Interval of East Greenland

**DOI:** 10.1371/journal.pone.0060614

**Published:** 2013-04-10

**Authors:** Karen L. Bacon, Claire M. Belcher, Matthew Haworth, Jennifer C. McElwain

**Affiliations:** 1 School of Biology and Environmental Science, University College Dublin, Belfield, Dublin, Ireland; 2 College of Life and Environmental Sciences, Hatherly Laboratories, University of Exeter, Exeter, United Kingdom; 3 CNR – Istituto di Biometeorologia (IBIMET), Firenze, Italy; Ludwig-Maximilians-Universität München, Germany

## Abstract

The Triassic–Jurassic boundary (Tr–J; ∼201 Ma) is marked by a doubling in the concentration of atmospheric CO_2_, rising temperatures, and ecosystem instability. This appears to have been driven by a major perturbation in the global carbon cycle due to massive volcanism in the Central Atlantic Magmatic Province. It is hypothesized that this volcanism also likely delivered sulphur dioxide (SO_2_) to the atmosphere. The role that SO_2_ may have played in leading to ecosystem instability at the time has not received much attention. To date, little direct evidence has been presented from the fossil record capable of implicating SO_2_ as a cause of plant extinctions at this time. In order to address this, we performed a physiognomic leaf analysis on well-preserved fossil leaves, including Ginkgoales, bennettites, and conifers from nine plant beds that span the Tr–J boundary at Astartekløft, East Greenland. The physiognomic responses of fossil taxa were compared to the leaf size and shape variations observed in nearest living equivalent taxa exposed to simulated palaeoatmospheric treatments in controlled environment chambers. The modern taxa showed a statistically significant increase in leaf roundness when fumigated with SO_2_. A similar increase in leaf roundness was also observed in the Tr–J fossil taxa immediately prior to a sudden decrease in their relative abundances at Astartekløft. This research reveals that increases in atmospheric SO_2_ can likely be traced in the fossil record by analyzing physiognomic changes in fossil leaves. A pattern of relative abundance decline following increased leaf roundness for all six fossil taxa investigated supports the hypothesis that SO_2_ had a significant role in Tr–J plant extinctions. This finding highlights that the role of SO_2_ in plant biodiversity declines across other major geological boundaries coinciding with global scale volcanism should be further explored using leaf physiognomy.

## Introduction

The Triassic–Jurassic (Tr–J; ∼201 Ma) boundary interval marks a period of intense climatic change and major biodiversity loss and saw rearrangement of the structure of terrestrial and marine environments. Several studies have identified a significant increase in background CO_2_ levels across the boundary [1]–[4] and report a large negative stable carbon isotope excursion [5]–[9]. These are postulated to be due to emissions from Central Atlantic Magmatic Province (CAMP) volcanism [10], [11]. Tanner et al., [12], suggested that the environmental degradation observed across the boundary may have been, in part, caused by emissions of sulphur dioxide (SO_2_) and other volcanic gases. The same study [12] further suggested that repeated pulses of CAMP volcanism could have led to a cumulative effect of SO_2_ in the stratosphere prolonging atmospheric acidification and the resultant acid rains over much of northern Pangaea. More recently, van de Schootbrugge et al., [13] suggested that SO_2_ may have had a significant role in causing direct environmental stress to plants through soil acidification in response to emissions from CAMP activity and indirectly by the intrusion of CAMP basalts into coal and evaporate deposits.

To date, few studies have examined the influence of SO_2_ on vegetation across the Tr–J. This study explores the physiognomic responses of a group of gymnosperms to fumigation with SO_2_ by undertaking plant growth experiments in simulated palaeoatmospheric treatments and comparing the results to physiognomic measurements of fossil leaves. Many previous studies have identified climate-related signals in leaf shape (e.g. [14]–[19]), but few have attempted to determine if exposure to high levels of atmospheric SO_2_ can also influence leaf physiognomy. Such studies have identified the potential negative effects of SO_2_ on leaf development and functioning e.g. [20]–[24]; however, this research represents the first time that the effects of elevated atmospheric SO_2_ on leaf physiognomy have been examined in detail and compared directly to physiognomic changes observed in leaf fossils of Tr–J age.

## Methods

### Simulated palaeoatmosphere treatments

Five nearest living equivalent (NLE) taxa were selected as analogues for abundant Late Triassic and Early Jurassic fossil taxa – *Agathis australis* and *Nageia nagi* were selected as NLEs for broad-leaved conifers, such as *Podozamites*; *Ginkgo biloba* was selected for ginkgophytes, such as *Ginkgoites* and *Baiera*; *Lepidozamia hopei* and *L. peroffskyana* for the Bennettites, such as *Anomozamites* and *Pterophyllum*. Three plants of each NLE species were placed in each of three Conviron BDW 40 walk-in controlled atmosphere and environment chambers, each with differing levels of SO_2_, CO_2_ and O_2_. The Tr–J boundary interval is characterized by high CO_2_ [1]–[4], [25], and hypothesized to have had elevated SO_2_ [12], [13], [26] and sub-ambient O_2_ [27]. The chambers therefore recreated the following conditions: 1) a Tr–J type atmosphere with elevated CO_2_ (1,500 ppm), SO_2_ (0.2 ppm), and sub-ambient O_2_ (13%); 2) an elevated SO_2_ treatment with ambient CO_2_ (380 ppm) and O_2_ (21%) and fumigation with SO_2_ (0.2 ppm); and 3) a control treatment with ambient CO_2_ and O_2_ and no SO_2_. Conditions within the chambers were monitored as outlined by Haworth et al., [28]. Atmospheric concentrations of gases were monitored as follows: CO_2_ by a WMA-4 IRGA (PP-systems, Amesbury, MA, USA), O_2_ by an OP-1 Oxygen Sensor (PP-systems) and SO_2_ by a Horriba APSA-370 Air Pollution Monitor. All other growth conditions remained constant between chambers. Plants were exposed to 16 hours of light each day in a simulated day/night program (5.00–6.00 dawn; 6.00–9.00 light intensity rises from 300 to 600 µmol m^−2^ s^−1^; 9.00–17.00 midday light intensity of 600 µmol m^−2^ s^−1^; 17.00–20.00 light intensity decreases 600 to 300 µmol m^−2^ s^−1^; 20.00–21.00 dusk); a standard temperature regime (night time temperature of 18°C rising to a midday peak of 28°C); relative humidity of 80%; downward ventilation to ensure mixing of atmospheric gases; and also received 60 ml of water each day (see [28]). Additionally, to avoid any potential chamber effects the plants in both sulphur-containing treatments were rotated between chambers every three months [29]. Leaves were sampled from mature new growth material only to ensure that the sampled leaves were those that had grown and developed under the simulated palaeoatmospheric treatments.

### Ethics statement

A permit was obtained from the Geological Survey of Denmark and Greenland to collect rock samples from Greenland (GEUS reference number 512–220). The land is not privately owned or protected and no living material or species were sampled.

### Astartekløft, East Greenland

Over 3,000 fossil leaves were collected from Astartekløft, East Greenland (see [25] for details). Astartekløft consists of nine fossiliferous plant beds: beds 1, 1.5, 2, 3, 4 and 5 are crevasse splay deposits; bed 6 a poorly developed coal and beds 7 and 8 are channel deposits [25], [30]. The site is noted for hosting a diverse and well-preserved fossil flora [25], [31]. Bed 5 marks the latest Rhaetian [32] and contains the peak turnover in vegetation [25], [33]. Mander et al., [34] correlate the Astartekløft section to the St Audries Bay, UK, section and place the likely incidents of CAMP volcanism within the Rhaetian beds, particularly beds 2–4, which also show the onset of ecosystem instability in both leaf macrofossils [25] and sporomorphs [34], [35]. Of the collected macrofossil leaves, many were complete or near complete fossils that allowed a full physiognomic analysis. The taxa selected for analysis were *Anomozamites*, *Pterophyllum*, *Elatocladus*, *Podozamites*, *Ginkgoites* and *Baiera*. These taxa were selected because each taxon was an important ecological component of at least two beds at Astartekløft [25] and provided sufficiently well-preserved samples to generate statistically meaningful analyses.

### Digital leaf physiognomy

Approximately 20 leaves were randomly selected from each plant in each chamber treatment, and dried flat at 40°C to preserve leaf shape. For the cycads, the largest frond from each plant in each treatment was selected and pinnae from the fronds were measured. For *G. biloba*, and *N. nagi*, there were some plants that did not produce 20 new leaves within treatment conditions and in each case all produced leaves were analysed (see Tables S1, S2, S3, S4, and S5 for full list of samples and measurements). Each leaf was photographed against a white background using a 10.1 megapixel Canon 1000D digital single-lens reflective camera that produced high-quality images with 3888 x 2592 pixel resolution. The resulting digital images were analysed using ImageJ (1.39u – documentation and downloads at website http://rsbweb.nih.gov/ij/, National Institutes of Health, Bethesda, Maryland, USA) to determine leaf or leaflet area, perimeter, shape factor (4π × (leaf area/leaf perimeter^2^)) and compactness (leaf perimeter^2^/leaf area). The fossil leaves were photographed using cross-polarised light against a black backdrop and were analysed using the same protocol as the extant leaves in ImageJ (see Tables S6, S7, S8, S9 S10, and S11 for full list of samples and measurements). Statistical analysis was preformed in PAST (http://nhm2.uio.no/norlex/past/download.html). Shape factor and compactness were analysed as a means of simply and efficiently tracking changes in leaf shape. Shape factor in particular has been previously used to identify climate-related shape changes in extant floras (e.g [15], [17], [18], [36]).

## Results

### Simulated palaeoatmospheric treatments

**Figure 1:**
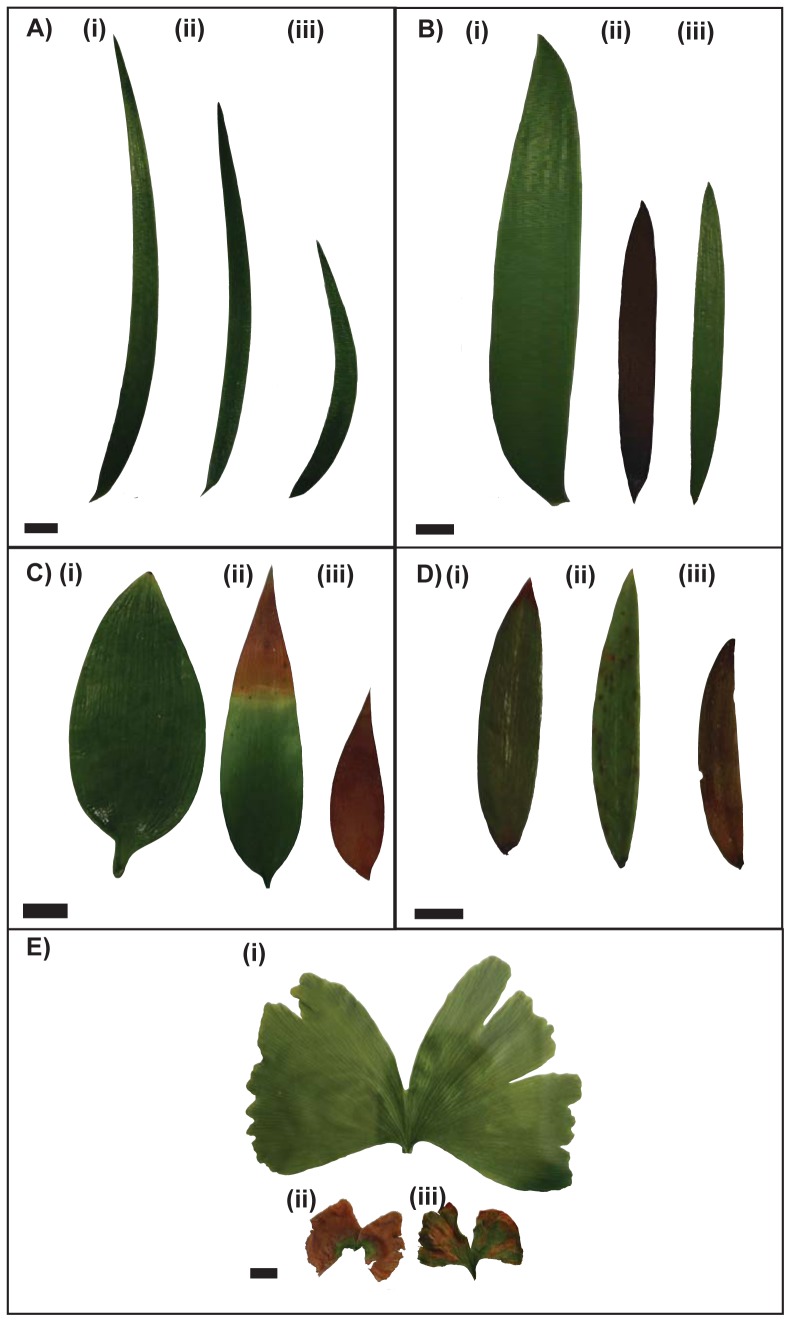
Examples of leaf physiognomy for each nearest living equivalent species in the study. *Lepidozamia peroffskyana* (A); *Lepidozamia hopei* (B); *Nageia nagi* (C); *Agathis australis* (D); *Ginkgo biloba* (E). Lower case Roman numerals indicate the simulated palaeoatmospheric treatment that the leaf grew in: (i) control; (ii) elevated SO_2_ and (iii) Tr–J type atmosphere. The scale bar in each image is 10 mm.

**Figure 2:**
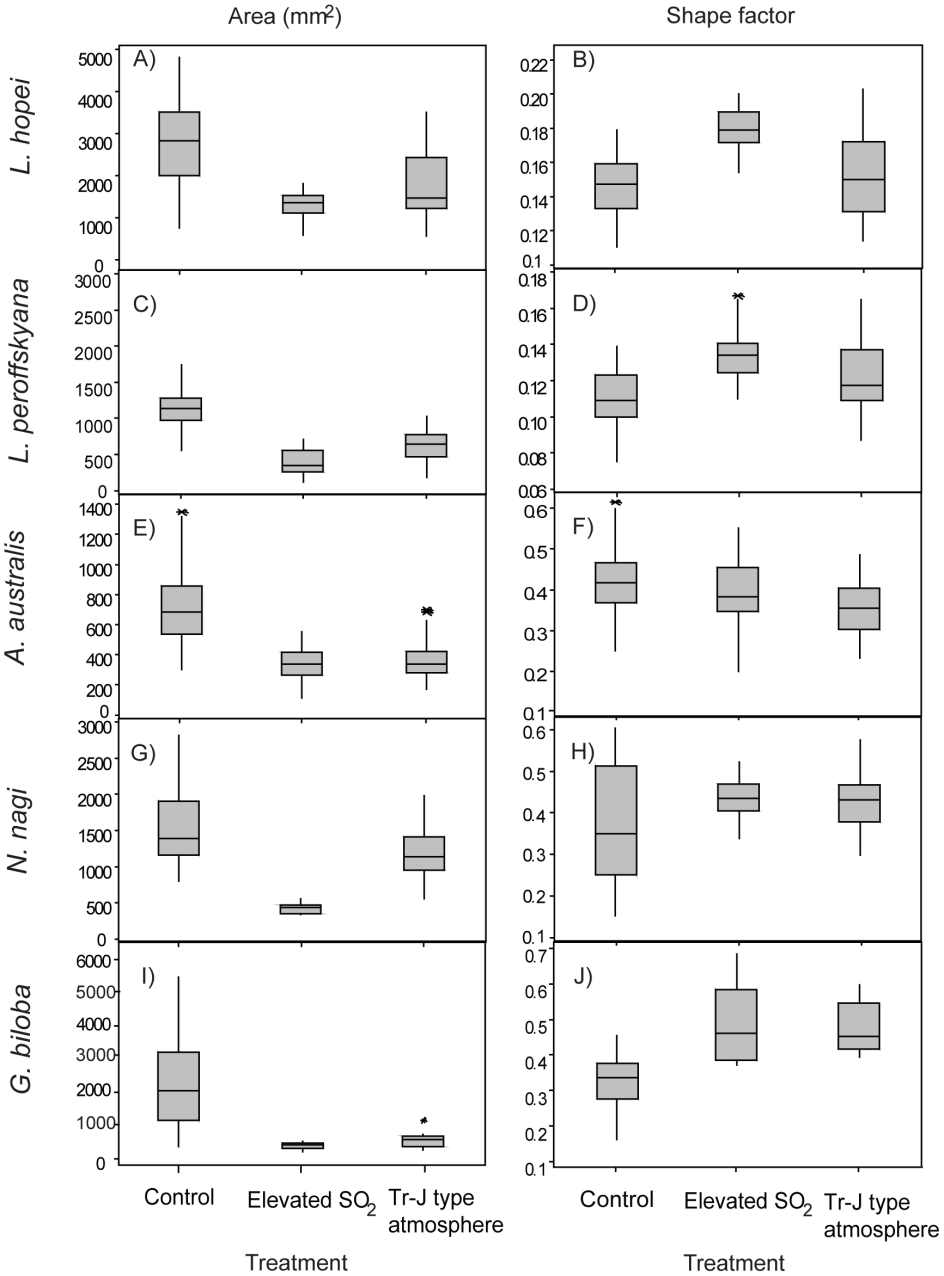
Box plots showing the range of values for area and shape factor for each nearest living equivalent species. The box represents the lower 25 percentile, the median value and the upper 25% percentile and the whiskers represent the range of the data. Stars represent outliers (values over twice the value of the median). *Lepidozamia hopei* (A area and B shape factor); L. *peroffskyana* (C area and D shape factor); *Agathis australis* (E area and F shape factor); *Nageia nagi* (G area and H shape factor); *Ginkgo biloba* (I area and J shape factor).

The simulated palaeoatmospheric treatments revealed that fumigation with SO_2_ significantly altered leaf shape in the NLE taxa. When analysed with a Kruskal-Wallis test, three out of five species showed a significant increase in shape factor (became rounder) in both the Tr–J and elevated SO_2_ palaeoatmospheric treatments (at least p<0.001) and one species also showed a tendency towards increased roundness in these treatments compared to the control (see Tables 1, 2, 3, 4, and 5 for details). All species showed a decrease in area in both SO_2_-containing palaeoatmospheric treatments (at least p<0.001). This decrease was slightly less apparent, though still significant, in the Tr–J palaeoatmosphere treatment when compared to the elevated SO_2_ treatment. The increase in roundness was also reduced in the Tr–J simulated palaeoatmospheric treatment compared to the elevated SO_2_ treatment. This suggests that the addition of high CO_2_ and/or sub-ambient O_2_ in the Tr–J treatment somehow mitigates the effects of SO_2_ on decreasing leaf area and increasing shape factor. This may be due to an increase in stomatal densities on exposure to higher atmospheric CO_2_ [37], [38] which would reduce stomatal conductance and therefore reduce the amount of SO_2_ entering the leaf in this treatment. Figure 1 shows examples of typical leaves grown in each treatment for each species. From this it is clear that leaves in the SO_2_-containing treatments have a far smaller area than those in the control in most cases. Leaf area and shape factor changes for all species within the different treatments are shown in Figure 2. Compactness showed the same response as shape factor and perimeter showed the same response as area, so neither is shown in the figure (See Tables S1, S2, S3, S4, and S5 for a full list of measured values).

**Table 1:** Kruskal Wallis and Mann-Whitney pair-wise comparisons for each physiognomic trait in *Lepidozamia peroffskyana* in the different simulated palaeoatmospheric treatments.

Area (mm^2^)			Perimeter (mm)			Shape factor			Compactness		
H = 145.6; p = 2.4^e−32^			H = 140; p = 3.927^e−31^			H = 66.63; p = 3.4^e−15^			H = 66.69; p = 3.292^e−15^		
	Elevated SO_2_	Tr-J		Elevated SO_2_	Tr-J		Elevated SO_2_	Tr-J		Elevated SO_2_	Tr-J
Control	7.784^e−23^	5.524^e−21^	Control	1.061^e−22^	6.711^e−19^	Control	1.257^e−16^	0.0001376	Control	1.144^e−16^	0.0001376
Elevated SO_2_		4.979^e−11^	Elevated SO_2_		3.256^e−11^	Elevated SO_2_	0	1.028^e−5^	Elevated SO_2_		1.069^e−5^

**Table 2:** Kruskal Wallis and Mann-Whitney pair-wise comparisons for each physiognomic trait in *Lepidozamia hopei* in the different simulated palaeoatmospheric treatments.

Area (mm^2^)			Perimeter (mm)			Shape factor			Compactness		
H = 145.6; p = 2.4^e−32^			H = 47.72; p = 4.339^e−11^			H = 41.11; p = 1.182^e−9^			H = 41.05; p = 1.219^e−9^		
	Elevated SO_2_	Tr-J		Elevated SO_2_	Tr-J		Elevated SO_2_	Tr-J		Elevated SO_2_	Tr-J
Control	1.77^e−9^	6.834^e−6^	Control	4.806^e−10^	3.832^e−5^	Control	8.482^e−10^	0.6503	Control	7.759^e−10^	0.6503
Elevated SO_2_		0.001587	Elevated SO_2_		3.077^e−5^	Elevated SO_2_		5.24^e−8^	Elevated SO_2_		6.1^e−8^

**Table 3:** Kruskal Wallis and Mann-Whitney pair-wise comparisons for each physiognomic trait in *Agathis australis* in the different simulated palaeoatmospheric treatments.

Area (mm^2^)			Perimeter (mm)			Shape factor			Compactness		
H = 83.28 p = 8.242^e−19^			H = 61.96; p = 3.551^e−14^			H = 16.2; p = 0.0003032			H = 16.51; p = 0.0002594		
	Elevated SO_2_	Tr-J		Elevated SO_2_	Tr-J		Elevated SO_2_	Tr-J		Elevated SO_2_	Tr-J
Control	1.036^e−15^	9.606^e−15^	Control	2.675^e−12^	2.034^e−10^	Control	0.1026	6.852^e−5^	Control	0.07825	5.874^e−5^
Elevated SO_2_		0.6919	Elevated SO_2_		0.05119	Elevated SO_2_		0.01802	Elevated SO_2_		0.01992

**Table 4:** Kruskal Wallis and Mann-Whitney pair-wise comparisons for each physiognomic trait in *Nageia nagi* in the different simulated palaeoatmospheric treatments.

Area (mm2)			Perimeter (mm)			Shape factor			Compactness		
H = 52.56, p = 4.494^e−12^			H = 64.68; p = 9.006^e−15^			H = 8.217; p = 0.01643			H = 8.251; p = 0.01616		
	Elevated SO_2_	Tr-J		Elevated SO_2_	Tr-J		Elevated SO_2_	Tr-J		Elevated SO_2_	Tr-J
Control	7.126^e−10^	0.0009584	Control	7.126^e−10^	1.723^e−07^	Control	0.1061	0.005454	Control	0.1061	0.005311
Elevated SO_2_		3.917^e−10^	Elevated SO_2_		3.917^e−10^	Elevated SO_2_	0	0.7635	Elevated SO_2_	0	0.7823

**Table 5:** Kruskal Wallis and Mann-Whitney pair-wise comparisons for each physiognomic trait in *Ginkgo biloba* in the different simulated palaeoatmospheric treatments.

Area (mm2)			Perimeter (mm)			Shape factor			Compactness		
H = 30.22; p = 2.746^e−7^			H = 33.5; p = 5.303^e−8^			H = 30.9; p = 1.95^e−7^			H = 30.54; p = 2.339^e−7^		
	Elevated SO_2_	Tr-J		Elevated SO_2_	Tr-J		Elevated SO_2_	Tr-J		Elevated SO_2_	Tr-J
Control	1.241^e−5^	0.0001136	Control	6.297^e−6^	2.711^e−5^	Control	5.657^e−5^	1.653^e−5^	Control	6.894^e−5^	1.587^e−5^
Elevated SO_2_		0.2164	Elevated SO_2_		0.4799	Elevated SO_2_		0.8598	Elevated SO_2_		0.8598

There is a highly significant difference in terms of area in most taxa (Figure 2) in the two SO_2_-containing simulated palaeoatmospheric treatments compared to the control, with the leaves in the control generally larger than those in either of the SO_2_-containing treatments. All measurements of area are significantly different compared to the control at the p<0.05 level and more usually at the p<0.0001 level. The shape response of the leaves is a little less conserved. Generally there is a highly significant (at least p<0.0001) increase in leaf roundness (higher shape factor value) in the SO_2_-containing treatments compared to the control, but the level of response varies between species. *Lepidozamia hopei*, *L. peroffskyana* and *G. biloba* all significantly increase leaf roundness (Figure 2 (B), (D) and (J), respectively) in the elevated SO_2_ treatment and *L. peroffskyana and G. biloba* both had significantly rounder leaves in the Tr–J treatment as well (Figure 2 (D) and (J)). *Nageia nagi* showed no significant increase in roundness in the elevated SO_2_ treatment, but there was a trend towards rounder leaves when compared to the control, with the lower quartile value of leaf roundness in the elevated SO_2_ and Tr–J treatments greater than the median value in the control treatment (Figure 2 H). *Agathis australis* showed no significant difference in leaf roundness in the elevated SO_2_ treatment and a slight decrease in roundness in the Tr–J treatment (Figure 2 F). Despite this limited response to fumigation with SO_2_, both *A. australis* and *N. nagi* showed shape factor range values at least as variable as the other species in the study, suggesting that there is no phenotypic restriction on shape change in these taxa. However, the overall trend of most species was towards smaller, rounder leaves in the SO_2_-containing palaeoatmospheric treatments (Figure 2 B, D, F, H, J). When the response of different species are considered together, a spectrum of response to SO_2_ can be identified based upon leaf physiognomy: the broad-leaved conifers have the greatest resistance to elevated atmospheric SO_2_, the cycads have moderate resistance to SO_2_ and *G. biloba* has the greatest response (both in terms of leaf size decrease and leaf roundness increase) to atmospheric SO_2_.

### Fossil leaf physiognomy

**Figure 3:**
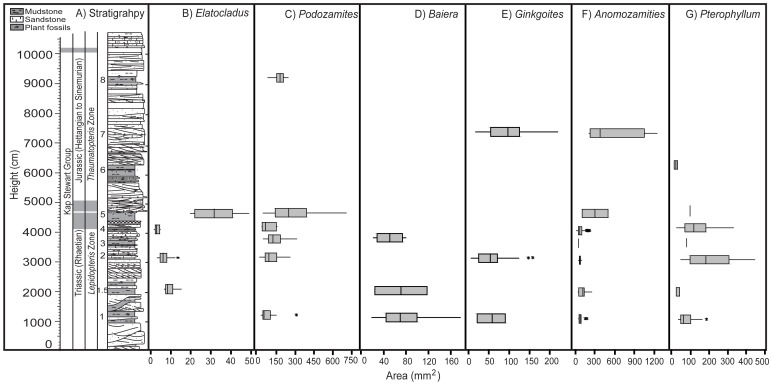
Astartekløft stratigraphic log (A) (after [5]; [25]) compared to area changes showed as box plots in the measured fossil taxa: *Elatocladus* (B); *Podozamites* (C); *Baiera* (D); *Ginkgoites* (E); *Anomozamites* (F); *Pterophyllum* (G).

**Figure 4:**
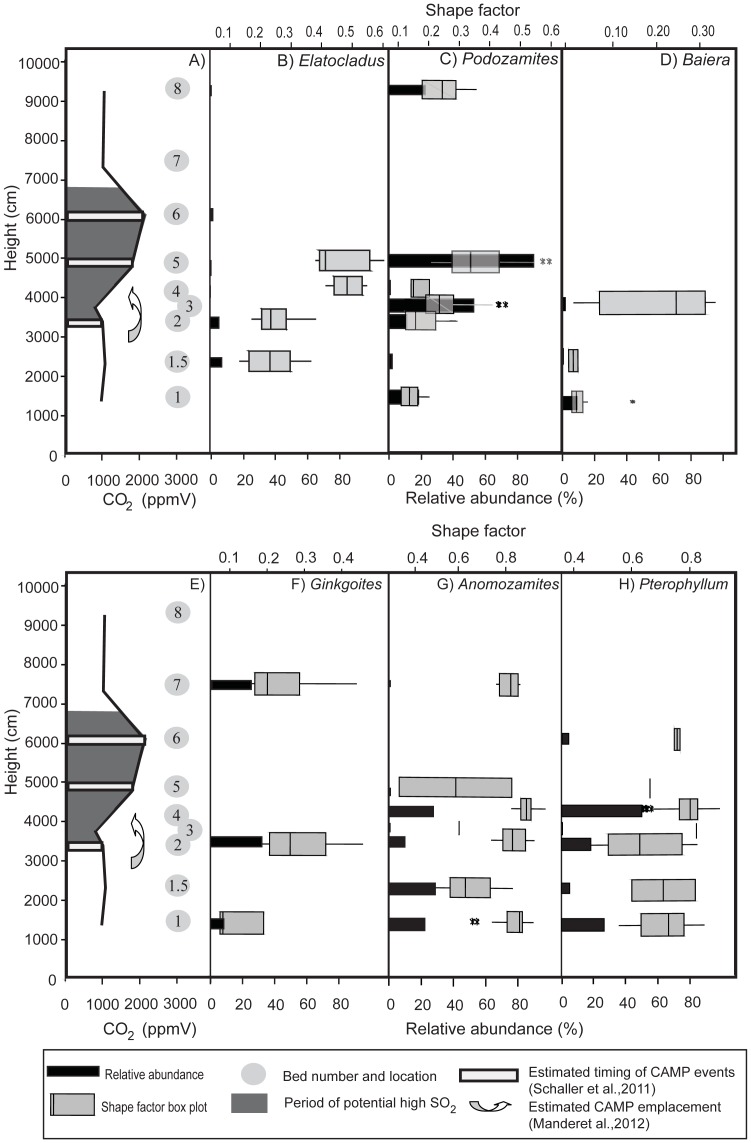
Comparison of atmospheric CO_2_ changes and timing of potential high SO_2_ with measured changes to fossil leaf relative abundance and shape factor at Astartekløft. Atmospheric CO_2_ changes at Astartekløft [4] with the time of suggested likely high SO_2_ [3]; [34] superimposed as grey (A) compared to shape factor changes as box plots and relative abundance changes as bars for each of the measured fossil taxa *Elatocladus* (B); *Podozamites* (C); *Baiera* (D); *Ginkgoites* (E); *Anomozamites* (F); *Pterophyllum* (G).

There are clear changes to leaf physiognomy in all taxa between the various beds; most apparent are increases in leaf area and leaf roundness in most taxa in different beds (Figure 3 and Figure 4; Tables S6, S7, S8, S9, S10, S11, S12, S13, S14, S15, S16, S17, S18, S19, S20, S21, S22, S23, S24, S25, S26, S27, S28, S29, S30, S31, S32, S33, S34, and S35). *Elatocladus* shows a significant increase in leaf area in bed 5 compared to the other beds (at least p<0.005 for beds 1 and 2 and p<0.05 for bed 4) (Figure 3 B). *Elatocladus* also shows a significant increase in shape factor in bed 4 (at least p<0.05) (Figure 4 B). Samples in bed 5 tend to be rounder than in beds 1.5 and 2 and are significantly rounder than the leaves in bed 2 (p = 0.058). *Podozamites* is present in large numbers in several beds and there is a clear change in leaf physiognomy between beds. There is a trend of increasing area and shape factor from bed 1 to bed 3, followed by a sudden decrease in both traits and then a sudden and highly significant increase in leaf area and shape factor in bed 5. The leaves in bed 8 are smaller and less round than those in bed 5 but larger and rounder than those from earlier Triassic beds (Figure 3 C and Figure 4 C). *Baiera*, similar to the other taxa in the study, also shows physiognomic variation between beds, with leaves in bed 3 being significantly rounder than those in bed 1 (p = 0.058) and bed 1.5 (p<0.005), although there is no significant difference in leaf area between any of these beds (Figure 3 D and Figure 4 D). *Ginkgoites* (Figure 3 E) have significantly larger leaves in bed 7 than in the two Triassic beds. Bed 2 has leaves that are significantly rounder than the leaves in bed 1 (p<0.05) and although the leaves in bed 2 are not significantly rounder than those of bed 7, they do tend towards being rounder, with higher median and maximum shape factor values (Figure 4 F). The bennettites also show significant variations in leaf physiognomy between beds. In particular, *Anomozamites* leaves in bed 4 are significantly rounder than the leaves in all other beds (at least p<0.05), except for bed 3 (p<0.1) (Figure 4 G). The leaves in beds 5 and 7 are also generally larger than those in the Triassic beds (Figure 3 F); however, there are only very few leaves in both beds, making statistical comparisons difficult. *Pterophyllum* has largest leaves in beds 2 and 4, and both are generally significantly (approximately p<0.05) larger than those in other beds with the leaves in bed 4 being larger than those in bed 2 (Figure 3 G). In terms of shape, the roundest *Pterophyllum* leaves are in bed 4 (Figure 4 H), and they are significantly larger than the leaves in beds 1 and 2 (p<0.005).

Overall, significant changes to leaf physiognomy occur throughout the Astartekløft section within each genus investigated. Figure 4 compares the changes in shape factor to variations in relative abundance of each taxon [25], and reveals a clear pattern of increasing shape factor, followed in the next bed by a decrease in relative abundance of the taxon. For example, *Ginkgoites* has its greatest leaf roundness values in bed 2 and a relative abundance of ∼20% [25]. This is followed by local absence from the macrofossil record until bed 7. *Anomozamites* and *Pterophyllum* account for ∼77% of the vegetation in bed 4, where maximum shape factor values are recorded for both taxa, and both have significantly reduced relative abundance (to approximately <1%) in the following beds. A similar pattern of increased shape factor followed by decreased relative abundance can be seen for *Podozamites*, *Elatocladus* and *Baiera* (Figure 4).

Figure 4 also compares the changes in shape factor and relative abundance with increasing atmospheric CO_2_ [4] and the most likely period of elevated atmospheric SO_2_ [3]. Mander et al., [34] suggest that beds 2–4 represent the times of likely CAMP emplacement and the continued pulses of CAMP activity suggested by Schaller et al., [3] infer that periods of fumigation with SO_2_ might be expected throughout deposition up to bed 6. The results highlight that the beds in which each taxon has greatest shape factor values correlates with a period of elevated CAMP activity and suggests an increasingly negative impact of elevated volcanic emissions as CAMP activity increases and more taxa respond to the atmospheric upheaval by first altering leaf shape and then decreasing relative abundance.

## Discussion

The increase in leaf area with increasing atmospheric CO_2_ is consistent with previous findings in free air carbon dioxide experiments [39]; however, the increase in leaf roundness was more unexpected given the suggestion that increased temperatures and associated increasing transpirative stress should lead to an evolutionary pressure of decreasing leaf area or increasing leaf dissection as atmospheric CO_2_ increased across the boundary [1]. Exposure to elevated levels of atmospheric SO_2_ has been known to induce a variety of responses in plants, including variation in stomatal numbers [35], stomatal, cuticle and bark damage [23], [24], water stress [21], and decreases in net photosynthesis [20]. Some species have shown evidence of adaptation to elevated levels of atmospheric SO_2_; for example, *Agrostis canina* growing at volcanic vents at Mefite di Ansanto, Italy [28], and various species of Hawaiian plants grow well in close proximity to volcanic gases [40], while others, such as *Ginkgo biloba*, have been shown to have different resistances to atmospheric SO_2_ depending on whether they are exposed to sulphur as a dry atmospheric pollutant or in acid rains [22].

The increase in leaf roundness in the SO_2_-containing simulated palaeoatmospheric treatments coupled to the fossil record of leaf changes at Astartekløft suggest that leaf physiognomy may serve as a marker for the presence of SO_2_ and other phytotoxic volcanic gases in fossil assemblages at times of suspected elevated SO_2_ due to volcanic activity. In the latest Triassic beds at Astartekløft the conifer leaves, most notably *Podozamites*, were seen to increase leaf roundness dramatically, in a similar manner to the increase in roundness observed in the elevated SO_2_ simulated palaeoatmospheric treatments. *Podozamites* leaves in bed 5 (the boundary bed) are significantly rounder than those in any of the other beds. *Elatocladus* leaves in bed 5 are also rounder than those of the older beds, although there are far fewer leaves available to measure for this taxon. When the individual patterns of shape change and relative abundance are considered for all fossil taxa, a further correspondence between SO_2_ and leaf physiognomy can be observed. For each of the fossil taxa investigated in this study a sudden increase in leaf roundness is followed by a major decline in the group's relative abundance in the following bed (Figure 4). Additionally, the order of response sensitivity, highlighted in Figure 4, shows that *Baiera* and *Ginkgoites* respond with increased roundness and decreased relative abundance before other taxa, then the bennettites respond and finally the conifers respond at peak CO_2_ and likely peak SO_2_ in beds 5 and 6. The timing of peak SO_2_ to beds 5 and 6 is further supported by an increase in the ratio of stomatal density to stomatal index in *Ginkgoites* fossil cuticle fragments in the same beds [37]. This order of sensitivity is similar to that observed in the simulated palaeoatmospheic treatments, where all of the NLE species included in the study responded negatively to exposure to SO_2_, but the magnitude of response differed between species. *Nageia nagi* showed the least marked response to SO_2_ exposure, producing leaves of a similar area and shape to that of the control in both SO_2_-containing treatments; however, in the SO_2_ treatment the leaves were a red/orange colour suggesting the onset of senescence and a reduced lifespan compared to the other two treatments (Figure 1 C). *Ginkgo biloba* was the most severely effected plant in the experiments (Figure 1 E), and the plants produced very few, poorly developed, small leaves. The other three species expressed responses between the two extremes of *N. nagi* and *G. biloba*. If both decrease in area and increase in shape factor are considered to indicate response plasticity within a taxon or species to elevated levels of atmospheric SO_2_, then the magnitude of response sensitivity can be ranked from least to most responsive as follows: *N. nagi*<*A. australis*<*L. hopei* and *L. peroffskyana*<*G. biloba*.

**Figure 5:**
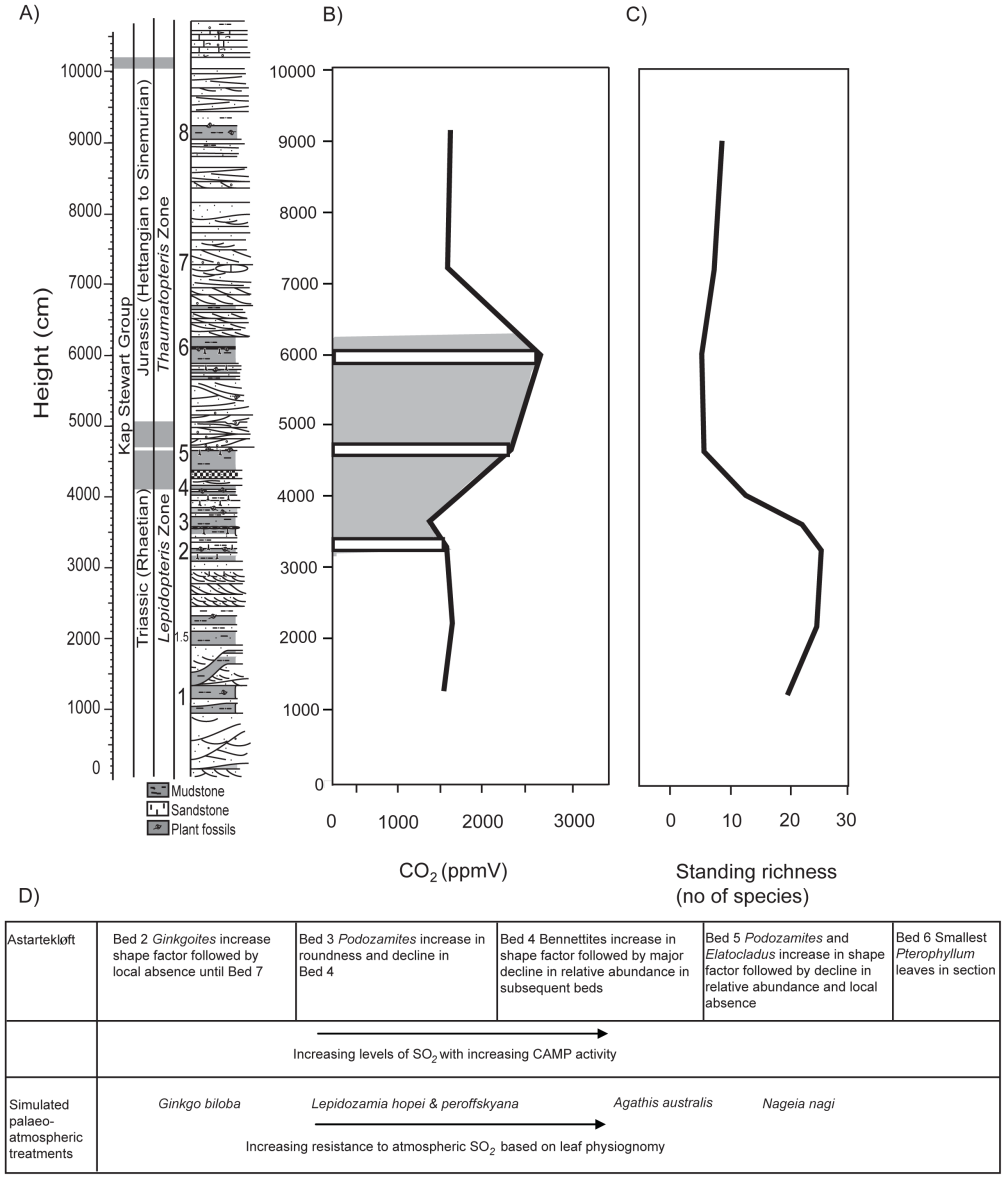
Summary of atmospheric changes compared to standing fossil richness recorded at Astartekløft and SO_2_ responsiveness of both fossil and NLE taxa. Astartekløft stratigraphic log (A) (after [5]; [25]) compared to atmospheric CO_2_ changes (B) [4] with timing of likely high SO_2_ [3]; [34] superimposed in grey, standing species richness (C) [25] and summarized responsiveness of both fossil and NLE taxa (D).

When this pattern of increased leaf shape sensitivity to SO_2_ among the NLEs is compared to both the physiognomic and relative abundance responses of the fossil taxa from Astartekløft across the Tr–J boundary, an interesting pattern emerges (Figure 4 and Figure 5). Each of the fossil taxa investigated shows an increase in shape factor that corresponds with one of the three periods of CAMP activity identified by Schaller et al., [3] and fall within the period of likely CAMP activity correlated to St Audries Bay by Mander et al., [34]. Maximum leaf roundness for each fossil taxon is either contemporaneous with or immediately followed by a sharp decline in relative abundance (Figure 4). *Ginkgoites* has rounder leaves in bed 2 than in bed 1 and then becomes locally absent from the macrofossil record until bed 7. *Baiera* has significantly increased shape factor in bed 3 compared to beds 1.5 and 2 and is not recorded upsection again at Astartekløft. These two taxa become rounder at the same time as or slightly after Schaller et al., [3] propose the first burst of CAMP and during the period highlighted by Mander et al., [34] for CAMP emplacement. Although none of the other fossil taxa show an increase in roundness at bed 2, the fact that fossil leaf physiognomic changes at Astartekløft are recorded first in *Ginkgoites* before any other taxa, is consistent with the rank order of response sensitivity observed in the simulated palaeoatmosphere treatments (*G. biloba* > *L. hopei* and *L. peroffskyana* > *N. nagi* and *A. australis*). Our predictions, based on this rank order are that *Anomozamites* and or *Pterophyllum* should respond next in the Astartekløft section followed by *Podozamites*. These predictions are borne out as *Anomozamites* and *Pterophyllum*, both show increased roundness for the first time in bed 4. Both become locally rare or absent for the rest of the section prior to maximum leaf roundness for *Podozamites* and *Elatocladus* in bed 5. *Podozamites* has maximum roundness in bed 5 and then becomes locally absent until bed 8. Bed 5 and bed 6 record the highest CO_2_ levels [4] and the lowest biodiversity [25], [33]–[35] of the section and both correspond to a large peak in emission of CO_2_ [3], [4]. The conifers, *Podozamites* and *Elatocladus*, both show increased roundness in bed 5 compared to all other beds in which these taxa occur. *Podozamites* then becomes locally absent until bed 8 and *Elatocladus*, although remaining present, produces extremely small and fragmented leaves in bed 6 that could not be measured accurately. Both *Elatocladus* and *Pterophyllum* produce very small leaves in bed 6, when CO_2_ reaches its peak and when SO_2_ would be expected to also peak due to increased volcanic activity [3], [34]. This agrees with the suggestion by Tanner et al., [12] and van der Schootbrugge et al., [13], [41] that repeated pulses of CAMP activity could have led to a build up of aerosols that may have negatively impacted upon plant growth.

Considered together with the palaeoatmospheric treatment results, the consistent increase in leaf roundness suggests that atmospheric SO_2_ increased at Astartekløft across the Tr–J boundary. The palaeoatmospheric treatments revealed that *Ginkgo biloba*, the nearest living equivalent and nearest living relative for Mesozoic *Ginkgoites*, developed leaves only rarely in the SO_2_-containing treatments, and those leaves that did grow were poorly developed, rounder and very small compared to control treatment leaves. This suggests that *Ginkgoites* leaves decreased their fossilization potential during periods of elevated atmospheric SO_2_ at Astartekløft. The increased roundness of the *Podozamites* leaves and the absence of *Ginkgoites* macrofossils but presence of *Ginkgoites* cuticles at the boundary [4], at a time likely to have suffered the cumulative effects of CAMP eruptions [3], [13], [34] suggest that SO_2_ may have had a significant role in leaf development at Astartekløft at this time. Additionally, Haworth et al., [37] identified a rise in the ratio of stomatal density (SD) to stomatal index (SI) in six species (including the five NLE taxa in this study) when leaves developed in simulated palaeoatmospheric treatments of elevated SO_2_. The same study identified an increase in the SD/SI ratio of fossil Ginkgoales across the Tr–J boundary at Astartekløft. Moreover, Mander et al, [42] found a possible increase in the chemical damage of pollen and spores in plant bed 5 at Astartekløft and van de Schootbrugge et al., [13] suggest that Trilete bed dark zones in pollen preservation in Germany across the Tr–J boundary were caused by acid rain due to CAMP activity. Both of these observations lend further support to the hypothesis that SO_2_ was elevated across the Tr–J boundary. One potential direction for further investigation might be to examine the cuticular micromorphology and stomata of the preserved fossil cuticles for characteristic damage [22], [24], as has recently been done by Bartiromo et al., [43], and compare this to changes in leaf shape and/or alteration to SD/SI ratio [37] by exposure to SO_2_.

## Conclusions

The results of the simulated palaeoatmospheric treatments suggest that exposure to elevated atmospheric SO_2_ leads to increased roundness in these gymnosperms, and that this can be tracked in a variety of different fossil taxa across the Tr–J boundary of Astartekløft, East Greenland, during the period that corresponds to likely increased emission of SO_2_. Although it is possible that leaf shape changes may be due to variations in light or transipiration, these findings are supported by indications of elevated SO_2_ in the SD/SI ratios of fossil *Ginkgoites* across the boundary at the same site [37] and it seems most likely that physiognomic changes recorded here are correlated to emissions of volcanic gases. Our findings indicate: 1) that the presence of elevated levels of atmospheric SO_2_ can likely be traced in the fossil record by tracking physiognomic changes in plant leaf fossils, although the observed change in leaf physiognomy could also have been promoted in part by other phytotoxic volcanic gases, and 2) that SO_2_ may be an important driver of biodiversity loss across the Tr–J boundary of Astartekløft, (e.g. [12], [13], [38]). The findings further highlight the importance of considering SO_2_ as a driver of changes in plant diversity and ecosystem stability across periods of global change that correlate to large igneous province volcanism and that variation in plant diversity should not be exclusively linked to increasing atmospheric CO_2_ and global temperature changes.

## Supporting Information

Table S1 All measured values for each leaf analysed from the simulated palaeoatmospheric treatments in the controlled environment chambers for *Agathis australis*.
(DOC)

Table S2 All measured values for each leaf analysed from the simulated palaeoatmospheric treatments in the controlled environment chambers for *Nageia nagi*.
(DOC)

Table S3 All measured values for each leaf analysed from the simulated palaeoatmospheric treatments in the controlled environment chambers for *Lepidozamia peroffskyana*.
(DOC)

Table S4 All measured values for each leaf analysed from the simulated palaeoatmospheric treatments in the controlled environment chambers for *Lepidozamia hopei*.
(DOC)

Table S5 All measured values for each leaf analysed from the simulated palaeoatmospheric treatments in the controlled environment chambers for *Ginkgo biloba*.
(DOC)

Table S6 All measured values for all fossil *Elatocladus* leaves measured in the analysis.
(DOC)

Table S7 All measured values for all fossil *Podozamites* leaves measured in the analysis.
(DOC)

Table S8 All measured values for all fossil *Baiera* leaves measured in the analysis.
(DOC)

Table S9 All measured values for all fossil *Ginkgoites* leaves measured in the analysis.
(DOC)

Table S10 All measured values for all fossil *Anomozamites* leaves measured in the analysis.
(DOC)

Table S11 All measured values for all fossil *Pterophyllum* leaves measured in the analysis.
(DOC)

Table S12 Kruskal Wallis and Mann-Whitney U pair-wise comparisons for area in *Anomozamites* in the different beds in which leaves are present at Astartekløft, East Greenland.
(DOC)

Table S13 Kruskal Wallis and Mann-Whitney U pair-wise comparisons for perimeter in *Anomozamites* in the different beds in which leaves are present at Astartekløft, East Greenland.
(DOC)

Table S14 Kruskal Wallis and Mann-Whitney U pair-wise comparisons for shape factor in *Anomozamites* in the different beds in which leaves are present at Astartekløft, East Greenland.
(DOC)

Table S15 Kruskal Wallis and Mann-Whitney U pair-wise comparisons for compactness in *Anomozamites* in the different beds in which leaves are present at Astartekløft, East Greenland.
(DOC)

Table S16 Kruskal Wallis and Mann-Whitney U pair-wise comparisons for area in *Pterophyllum* in the different beds in which leaves are present at Astartekløft, East Greenland.
(DOC)

Table S17 Kruskal Wallis and Mann-Whitney U pair-wise comparisons for perimeter in *Pterophyllum* in the different beds in which leaves are present at Astartekløft, East Greenland.
(DOC)

Table S18 Kruskal Wallis and Mann-Whitney U pair-wise comparisons for shape factor in *Pterophyllum* in the different beds in which leaves are present at Astartekløft, East Greenland.
(DOC)

Table S19 Kruskal Wallis and Mann-Whitney U pair-wise comparisons for compactness in *Pterophyllum* in the different beds in which leaves are present at Astartekløft, East Greenland.
(DOC)

Table S20 Kruskal Wallis and Mann-Whitney U pair-wise comparisons for area in *Elatocladus* in the different beds in which leaves are present at Astartekløft, East Greenland.
(DOC)

Table S21 Kruskal Wallis and Mann-Whitney U pair-wise comparisons for perimeter in *Elatocladus* in the different beds in which leaves are present at Astartekløft, East Greenland.
(DOC)

Table S22 Kruskal Wallis and Mann-Whitney U pair-wise comparisons for shape factor in *Elatocladus* in the different beds in which leaves are present at Astartekløft, East Greenland.
(DOC)

Table S23 Kruskal Wallis and Mann-Whitney U pair-wise comparisons for compactness in *Elatocladus* in the different beds in which leaves are present at Astartekløft, East Greenland.
(DOC)

Table S24 Kruskal Wallis and Mann-Whitney U pair-wise comparisons for area in *Podozamites* in the different beds in which leaves are present at Astartekløft, East Greenland.
(DOC)

Table S25 Kruskal Wallis and Mann-Whitney U pair-wise comparisons for perimeter in *Podozamites* in the different beds in which leaves are present at Astartekløft, East Greenland.
(DOC)

Table S26 Kruskal Wallis and Mann-Whitney U pair-wise comparisons for shape factor in *Podozamites* in the different beds in which leaves are present at Astartekløft, East Greenland.
(DOC)

Table S27 Kruskal Wallis and Mann-Whitney U pair-wise comparisons for compactness in *Podozamites* in the different beds in which leaves are present at Astartekløft, East Greenland.
(DOC)

Table S28 Kruskal Wallis and Mann-Whitney U pair-wise comparisons for area in *Baiera* in the different beds in which leaves are present at Astartekløft, East Greenland.
(DOC)

Table S29 Kruskal Wallis and Mann-Whitney U pair-wise comparisons for perimeter in *Baiera* in the different beds in which leaves are present at Astartekløft, East Greenland.
(DOC)

Table S30 Kruskal Wallis and Mann-Whitney U pair-wise comparisons for shape factor in *Baiera* in the different beds in which leaves are present at Astartekløft, East Greenland.
(DOC)

Table S31 Kruskal Wallis and Mann-Whitney U pair-wise comparisons for compactness in *Baiera* in the different beds in which leaves are present at Astartekløft, East Greenland.
(DOC)

Table S32 Kruskal Wallis and Mann-Whitney U pair-wise comparisons for area in *Ginkgoites* in the different beds in which leaves are present at Astartekløft, East Greenland.
(DOC)

Table S33 Kruskal Wallis and Mann-Whitney U pair-wise comparisons for perimeter in *Ginkgoites* in the different beds in which leaves are present at Astartekløft, East Greenland.
(DOC)

Table S34 Kruskal Wallis and Mann-Whitney U pair-wise comparisons for shape factor in *Ginkgoites* in the different beds in which leaves are present at Astartekløft, East Greenland.
(DOC)

Table S35 Kruskal Wallis and Mann-Whitney U pair-wise comparisons for compactness in *Ginkgoites* in the different beds in which leaves are present at Astartekløft, East Greenland.
(DOC)
