# Supplementary material for: Increased Atmospheric SO2 Detected from Changes in Leaf Physiognomy across the Triassic–Jurassic Boundary Interval of East Greenland
Source: PLoS One. 2013 Apr 10;8(4):e60614. doi: 10.1371/journal.pone.0060614 (PMC3622679; doi:10.1371/journal.pone.0060614)
Supplement: Table S1 — All measured values for each leaf analysed from the simulated palaeoatmospheric treatments in the controlled environment chambers for Agathis australis. (DOC) [file pone.0060614.s001.doc]

Table S1: All measured values for each leaf analysed from the simulated palaeoatmospheric treatments in the controlled environment chambers for *Agathis australis*. Gray shading indicated that the value was an outlier (over twice the standard deviation of the mean value) and was not included in analyses.

| **Plant** | **Sample No** | **Area (mm^2^)** | **Perimeter (mm)** | **Shape factor** | **Compactness** |
| --- | --- | --- | --- | --- | --- |
| Plant 1 Control | 1 | 379.7 | 114.36 | 0.365 | 34.443533 |
| Plant 1 Control | 2 | 380.8 | 112.31 | 0.379 | 33.123782 |
| Plant 1 Control | 3 | 376.1 | 100.68 | 0.466 | 26.951509 |
| Plant 1 Control | 4 | 378.6 | 114.31 | 0.364 | 34.513408 |
| Plant 1 Control | 5 | 402.4 | 123.87 | 0.33 | 38.130658 |
| Plant 1 Control | 6 | 344.4 | 95.32 | 0.476 | 26.38183 |
| Plant 1 Control | 7 | 301.1 | 98.47 | 0.39 | 32.203058 |
| Plant 1 Control | 8 | 340.6 | 119.1 | 0.302 | 41.646536 |
| Plant 1 Control | 9 | 519.2 | 122.62 | 0.434 | 28.959292 |
| Plant 1 Control | 10 | 685.2 | 139.02 | 0.446 | 28.205722 |
| Plant 1 Control | 11 | 527 | 129.07 | 0.398 | 31.611129 |
| Plant 1 Control | 12 | 509 | 119.17 | 0.45 | 27.900764 |
| Plant 1 Control | 13 | 572.2 | 120.83 | 0.492 | 25.51536 |
| Plant 1 Control | 14 | 609.9 | 135.19 | 0.419 | 29.966119 |
| Plant 1 Control | 15 | 802.2 | 161.6 | 0.386 | 32.553677 |
| Plant 1 Control | 16 | 745 | 151.16 | 0.41 | 30.670263 |
| Plant 1 Control | 17 | 656.2 | 147.83 | 0.377 | 33.303427 |
| Plant 1 Control | 18 | 568.6 | 134.39 | 0.396 | 31.763405 |
| Plant 1 Control | 19 | 482.7 | 137.71 | 0.32 | 39.287433 |
| Plant 1 Control | 20 | 505.3 | 139.42 | 0.327 | 38.468111 |
| Plant 2 Control | 1 | 747.3 | 159.81 | 0.368 | 34.175346 |
| Plant 2 Control | 2 | 625 | 146.12 | 0.368 | 34.161687 |
| Plant 2 Control | 3 | 709.3 | 145.37 | 0.422 | 29.793369 |
| Plant 2 Control | 4 | 1146.8 | 183.4 | 0.428 | 29.329927 |
| Plant 2 Control | 5 | 918.4 | 172.98 | 0.386 | 32.580662 |
| Plant 2 Control | 6 | 704.3 | 134.02 | 0.493 | 25.502429 |
| Plant 2 Control | 7 | 871.6 | 157.2 | 0.443 | 28.352272 |
| Plant 2 Control | 8 | 845.5 | 183.82 | 0.314 | 39.964273 |
| Plant 2 Control | 9 | 631.5 | 146.73 | 0.369 | 34.092942 |
| Plant 2 Control | 10 | 503.3 | 133.35 | 0.356 | 35.331259 |
| Plant 2 Control | 11 | 596 | 122.85 | 0.496 | 25.322353 |
| Plant 2 Control | 12 | 597.6 | 120.34 | 0.519 | 24.233125 |
| Plant 2 Control | 13 | 587.6 | 131.73 | 0.426 | 29.531642 |
| Plant 2 Control | 14 | 862 | 155.17 | 0.45 | 27.9324 |
| Plant 2 Control | 15 | 952.4 | 189.09 | 0.335 | 37.542029 |
| Plant 2 Control | 16 | 488.6 | 106.15 | 0.545 | 23.061446 |
| Plant 2 Control | 17 | 620.4 | 126.22 | 0.489 | 25.679382 |
| Plant 2 Control | 18 | 736.6 | 135.1 | 0.507 | 24.778727 |
| Plant 2 Control | 19 | 858.4 | 152.21 | 0.466 | 26.989613 |
| Plant 2 Control | 20 | 792.7 | 152.91 | 0.426 | 29.495986 |
| Plant 3 Control | 1 | 722.7 | 149.99 | 0.404 | 31.129099 |
| Plant 3 Control | 2 | 419.5 | 117.95 | 0.379 | 33.163772 |
| Plant 3 Control | 3 | 516.2 | 120 | 0.45 | 27.896164 |
| Plant 3 Control | 4 | 1280.8 | 178.33 | 0.506 | 24.829473 |
| Plant 3 Control | 5 | 1634.9 | 214.18 | 0.448 | 28.058641 |
| Plant 3 Control | 6 | 1045.1 | 175.05 | 0.429 | 29.320163 |
| Plant 3 Control | 7 | 948.6 | 141.24 | 0.598 | 21.029662 |
| Plant 3 Control | 8 | 652.2 | 115.54 | 0.614 | 20.468402 |
| Plant 3 Control | 9 | 808 | 159.53 | 0.399 | 31.497303 |
| Plant 3 Control | 10 | 666.2 | 182.46 | 0.251 | 49.972458 |
| Plant 3 Control | 11 | 877.5 | 154.04 | 0.465 | 27.040822 |
| Plant 3 Control | 12 | 1056 | 159.76 | 0.52 | 24.169752 |
| Plant 3 Control | 13 | 649.9 | 146.07 | 0.383 | 32.830351 |
| Plant 3 Control | 14 | 1350.8 | 226.16 | 0.332 | 37.865225 |
| Plant 3 Control | 15 | 1318.5 | 223.11 | 0.333 | 37.753562 |
| Plant 3 Control | 16 | 679.7 | 132.55 | 0.486 | 25.848908 |
| Plant 3 Control | 17 | 818.2 | 175.38 | 0.334 | 37.592452 |
| Plant 3 Control | 18 | 1202.3 | 186.82 | 0.433 | 29.029121 |
| Plant 3 Control | 19 | 717.2 | 172.14 | 0.304 | 41.31648 |
| Plant 3 Control | 20 | 807.6 | 156.59 | 0.414 | 30.362095 |
| Plant 1 Elevated SO_2_ | 1 | 383.3 | 114.54 | 0.367 | 34.227528 |
| Plant 1 Elevated SO_2_ | 2 | 466 | 130.64 | 0.343 | 36.624055 |
| Plant 1 Elevated SO_2_ | 3 | 444.7 | 136.24 | 0.301 | 41.73901 |
| Plant 1 Elevated SO_2_ | 4 | 420.5 | 119.88 | 0.368 | 34.176491 |
| Plant 1 Elevated SO_2_ | 5 | 473.9 | 172.4 | 0.2 | 62.717367 |
| Plant 1 Elevated SO_2_ | 6 | 400.9 | 129.12 | 0.302 | 41.586367 |
| Plant 1 Elevated SO_2_ | 7 | 284.3 | 97.7 | 0.374 | 33.57471 |
| Plant 1 Elevated SO_2_ | 8 | 300.7 | 111.37 | 0.305 | 41.248011 |
| Plant 1 Elevated SO_2_ | 9 | 403.6 | 114.25 | 0.389 | 32.341582 |
| Plant 1 Elevated SO_2_ | 10 | 437.3 | 141.84 | 0.273 | 46.00637 |
| Plant 1 Elevated SO_2_ | 11 | 415.1 | 116.1 | 0.387 | 32.472199 |
| Plant 1 Elevated SO_2_ | 12 | 263.3 | 91.51 | 0.395 | 31.80433 |
| Plant 1 Elevated SO_2_ | 13 | 292.1 | 97.91 | 0.383 | 32.818788 |
| Plant 1 Elevated SO2 | 14 | 283.7 | 103.97 | 0.33 | 38.102788 |
| Plant 1 Elevated SO2 | 15 | 287 | 100.62 | 0.356 | 35.276601 |
| Plant 1 Elevated SO_2_ | 16 | 341.6 | 107.22 | 0.373 | 33.653772 |
| Plant 1 Elevated SO_2_ | 17 | 181.6 | 94.9 | 0.253 | 49.592566 |
| Plant 1 Elevated SO_2_ | 18 | 172 | 76.15 | 0.373 | 33.714084 |
| Plant 1 Elevated SO_2_ | 19 | 408.6 | 115.05 | 0.388 | 32.394769 |
| Plant 1 Elevated SO_2_ | 20 | 266.4 | 97.3 | 0.354 | 35.537875 |
| Plant 2 Elevated SO_2_ | 1 | 539.5 | 121.74 | 0.457 | 27.471043 |
| Plant 2 Elevated SO_2_ | 2 | 496.4 | 114.52 | 0.476 | 26.419884 |
| Plant 2 Elevated SO_2_ | 3 | 510.3 | 146.17 | 0.3 | 41.86884 |
| Plant 2 Elevated SO_2_ | 4 | 509.9 | 131.14 | 0.373 | 33.727593 |
| Plant 2 Elevated SO_2_ | 5 | 396.9 | 127.82 | 0.305 | 41.163901 |
| Plant 2 Elevated SO_2_ | 6 | 387.2 | 119.61 | 0.34 | 36.94874 |
| Plant 2 Elevated SO_2_ | 7 | 515.5 | 126.03 | 0.408 | 30.811951 |
| Plant 2 Elevated SO_2_ | 8 | 535.6 | 133.94 | 0.375 | 33.495003 |
| Plant 2 Elevated SO_2_ | 9 | 377.6 | 94.21 | 0.535 | 23.505096 |
| Plant 2 Elevated SO_2_ | 10 | 313.6 | 84.79 | 0.548 | 22.925204 |
| Plant 2 Elevated SO_2_ | 11 | 445.7 | 113.37 | 0.436 | 28.837238 |
| Plant 2 Elevated SO_2_ | 12 | 389.5 | 100.6 | 0.484 | 25.982953 |
| Plant 2 Elevated SO_2_ | 13 | 436.8 | 118.72 | 0.389 | 32.267487 |
| Plant 2 Elevated SO_2_ | 14 | 551.4 | 135.69 | 0.376 | 33.390961 |
| Plant 2 Elevated SO_2_ | 15 | 416.7 | 122.78 | 0.347 | 36.176934 |
| Plant 2 Elevated SO_2_ | 16 | 339.1 | 110.42 | 0.349 | 35.955696 |
| Plant 2 Elevated SO_2_ | 17 | 336.4 | 107.19 | 0.368 | 34.154864 |
| Plant 2 Elevated SO_2_ | 18 | 367.5 | 125.06 | 0.295 | 42.557833 |
| Plant 2 Elevated SO_2_ | 19 | 414.5 | 128.11 | 0.317 | 39.595108 |
| Plant 2 Elevated SO_2_ | 20 | 441.4 | 144.95 | 0.264 | 47.599688 |
| Plant 3 Elevated SO_2_ | 1 | 365 | 91.23 | 0.551 | 22.802501 |
| Plant 3 Elevated SO_2_ | 2 | 288.4 | 84.24 | 0.511 | 24.606025 |
| Plant 3 Elevated SO_2_ | 3 | 316.4 | 90.24 | 0.488 | 25.737224 |
| Plant 3 Elevated SO_2_ | 4 | 257.3 | 84.06 | 0.458 | 27.462431 |
| Plant 3 Elevated SO_2_ | 5 | 269 | 86.32 | 0.454 | 27.699414 |
| Plant 3 Elevated SO_2_ | 6 | 365 | 91.23 | 0.551 | 22.802501 |
| Plant 3 Elevated SO_2_ | 7 | 288.4 | 84.24 | 0.511 | 24.606025 |
| Plant 3 Elevated SO_2_ | 8 | 316.4 | 90.24 | 0.488 | 25.737224 |
| Plant 3 Elevated SO_2_ | 9 | 257.3 | 84.06 | 0.458 | 27.462431 |
| Plant 3 Elevated SO_2_ | 10 | 269 | 86.32 | 0.454 | 27.699414 |
| Plant 3 Elevated SO_2_ | 11 | 145.1 | 68.96 | 0.383 | 32.773822 |
| Plant 3 Elevated SO_2_ | 12 | 165.8 | 74.47 | 0.376 | 33.448618 |
| Plant 3 Elevated SO_2_ | 13 | 231.7 | 89.87 | 0.361 | 34.858079 |
| Plant 3 Elevated SO_2_ | 14 | 161.7 | 72.86 | 0.383 | 32.829806 |
| Plant 3 Elevated SO_2_ | 15 | 168 | 75.14 | 0.374 | 33.60726 |
| Plant 3 Elevated SO_2_ | 16 | 191.5 | 76.08 | 0.416 | 30.225412 |
| Plant 3 Elevated SO_2_ | 17 | 213.7 | 79.41 | 0.426 | 29.508414 |
| Plant 3 Elevated SO_2_ | 18 | 112.9 | 53.69 | 0.492 | 25.532472 |
| Plant 3 Elevated SO_2_ | 19 | 126.2 | 60.09 | 0.439 | 28.611792 |
| Plant 3 Elevated SO_2_ | 20 | 132.6 | 63.25 | 0.416 | 30.170155 |
| Plant 1  Tr–J | 1 | 294.9 | 95.33 | 0.408 | 30.816578 |
| Plant 1  Tr–J | 2 | 271.6 | 92.31 | 0.4 | 31.373844 |
| Plant 1  Tr–J | 3 | 248.5 | 91.83 | 0.37 | 33.934603 |
| Plant 1  Tr–J | 4 | 326.7 | 106.17 | 0.364 | 34.502813 |
| Plant 1  Tr–J | 5 | 330.4 | 121.72 | 0.28 | 44.841884 |
| Plant 1  Tr–J | 6 | 229.3 | 100.94 | 0.283 | 44.43473 |
| Plant 1  Tr–J | 7 | 353.6 | 117.54 | 0.322 | 39.071413 |
| Plant 1  Tr–J | 8 | 274.9 | 102.53 | 0.329 | 38.240818 |
| Plant 1  Tr–J | 9 | 515.9 | 135.76 | 0.352 | 35.725485 |
| Plant 1  Tr–J | 10 | 624.2 | 135.7 | 0.426 | 29.500945 |
| Plant 1 Tr–J | 11 | 534.3 | 129.18 | 0.402 | 31.232402 |
| Plant 1  Tr–J | 12 | 611.6 | 140.65 | 0.389 | 32.345361 |
| Plant 1  Tr–J | 13 | 610.1 | 130.36 | 0.451 | 27.854007 |
| Plant 1  Tr–J | 14 | 682.4 | 152.13 | 0.37 | 33.914913 |
| Plant 1  Tr–J | 15 | 599.8 | 132.11 | 0.432 | 29.09812 |
| Plant 1  Tr–J | 16 | 577.5 | 130.56 | 0.426 | 29.516734 |
| Plant 1  Tr–J | 17 | 296 | 90.51 | 0.454 | 27.675879 |
| Plant 1  Tr–J | 18 | 697 | 147.22 | 0.404 | 31.095737 |
| Plant 1  Tr–J | 19 | 477.5 | 121.99 | 0.403 | 31.165571 |
| Plant 1  Tr–J | 20 | 375.2 | 110.19 | 0.388 | 32.36097 |
| Plant 2  Tr–J | 1 | 321.1 | 121.9 | 0.272 | 46.277203 |
| Plant 2  Tr–J | 2 | 240.8 | 96.07 | 0.328 | 38.32826 |
| Plant 2  Tr–J | 3 | 350.6 | 130.93 | 0.257 | 48.895222 |
| Plant 2  Tr–J | 4 | 293.2 | 118.63 | 0.262 | 47.998216 |
| Plant 2  Tr–J | 5 | 389.2 | 133.78 | 0.273 | 45.984297 |
| Plant 2  Tr–J | 6 | 211 | 102.49 | 0.252 | 49.782939 |
| Plant 2  Tr–J | 7 | 428.5 | 135.88 | 0.292 | 43.088388 |
| Plant 2  Tr–J | 8 | 488.7 | 179.95 | 0.19 | 66.261515 |
| Plant 2  Tr–J | 9 | 339.8 | 107.79 | 0.367 | 34.192714 |
| Plant 2  Tr–J | 10 | 445.9 | 128.59 | 0.339 | 37.083176 |
| Plant 2  Tr–J | 11 | 401.6 | 119.02 | 0.356 | 35.273308 |
| Plant 2  Tr–J | 12 | 335.2 | 111.46 | 0.339 | 37.062445 |
| Plant 2  Tr–J | 13 | 290 | 111.53 | 0.293 | 42.8929 |
| Plant 2  Tr–J | 14 | 523.8 | 133.1 | 0.372 | 33.821325 |
| Plant 2  Tr–J | 15 | 314.7 | 117.8 | 0.285 | 44.095456 |
| Plant 2  Tr–J | 16 | 367.3 | 118.07 | 0.331 | 37.954056 |
| Plant 2  Tr–J | 17 | 365.5 | 119.95 | 0.319 | 39.36526 |
| Plant 2  Tr–J | 18 | 370.8 | 115.81 | 0.347 | 36.170324 |
| Plant 2  Tr–J | 19 | 294.6 | 102.24 | 0.354 | 35.482069 |
| Plant 2  Tr–J | 20 | 454.4 | 129.26 | 0.342 | 36.769691 |
| Plant 3  Tr–J | 1 | 250.7 | 99.4 | 0.319 | 39.411089 |
| Plant 3  Tr–J | 2 | 223.6 | 95.25 | 0.31 | 40.574966 |
| Plant 3  Tr–J | 3 | 197.1 | 91.25 | 0.297 | 42.24537 |
| Plant 3  Tr–J | 4 | 384.2 | 112.15 | 0.384 | 32.737175 |
| Plant 3  Tr–J | 5 | 168.7 | 88.33 | 0.272 | 46.248897 |
| Plant 3  Tr–J | 6 | 335.1 | 95.73 | 0.46 | 27.347756 |
| Plant 3  Tr–J | 7 | 358.9 | 96.56 | 0.484 | 25.978918 |
| Plant 3  Tr–J | 8 | 281.5 | 93.01 | 0.409 | 30.731297 |
| Plant 3  Tr–J | 9 | 246.2 | 115.38 | 0.232 | 54.072073 |
| Plant 3  Tr–J | 10 | 276.2 | 89.36 | 0.435 | 28.910969 |
| Plant 3  Tr–J | 11 | 307.8 | 100.78 | 0.381 | 32.997428 |
| Plant 3  Tr–J | 12 | 209.3 | 83.57 | 0.377 | 33.368108 |
| Plant 3  Tr–J | 13 | 332.4 | 96.25 | 0.451 | 27.870224 |
| Plant 3  Tr–J | 14 | 241 | 86.23 | 0.407 | 30.853166 |
| Plant 3  Tr–J | 15 | 289.3 | 90.2 | 0.447 | 28.123194 |
| Plant 3  Tr–J | 16 | 190.7 | 71.22 | 0.473 | 26.598261 |
| Plant 3  Tr–J | 17 | 242.5 | 79.3 | 0.485 | 25.931918 |
| Plant 3  Tr–J | 18 | 324.3 | 99.43 | 0.412 | 30.485121 |
| Plant 3  Tr–J | 19 | 337 | 114.88 | 0.321 | 39.161467 |
| Plant 3  Tr–J | 20 | 363.2 | 123.57 | 0.299 | 42.041699 |
