# Supplementary material for: Increased Atmospheric SO2 Detected from Changes in Leaf Physiognomy across the Triassic–Jurassic Boundary Interval of East Greenland
Source: PLoS One. 2013 Apr 10;8(4):e60614. doi: 10.1371/journal.pone.0060614 (PMC3622679; doi:10.1371/journal.pone.0060614)
Supplement: Table S2 — All measured values for each leaf analysed from the simulated palaeoatmospheric treatments in the controlled environment chambers for Nageia nagi. (DOC) [file pone.0060614.s002.doc]

Table S2: All measured values for each leaf analysed from the simulated palaeoatmospheric treatments in the controlled environment chambers for *Nageia nagi*. Gray shading indicated that the value was an outlier (over twice the standard deviation of the mean value) and was not included in analyses.

| **Treatment** | **Sample No** | **Area (mm^2^)** | **Perimeter (mm)** | **Shape factor** | **Compactness** |
| --- | --- | --- | --- | --- | --- |
| Plant 1 Control | 1 | 1348.5 | 197.24 | 0.436 | 28.84955 |
| Plant 1 Control | 2 | 1338.4 | 229.55 | 0.319 | 39.37029 |
| Plant 1 Control | 3 | 1838.8 | 196.2 | 0.6 | 20.93454 |
| Plant 1 Control | 4 | 2652.3 | 254.79 | 0.513 | 24.47609 |
| Plant 1 Control | 5 | 2143.4 | 220.71 | 0.553 | 22.72693 |
| Plant 1 Control | 6 | 1708.9 | 222.96 | 0.432 | 29.08957 |
| Plant 1 Control | 7 | 992 | 192.27 | 0.337 | 37.26588 |
| Plant 1 Control | 8 | 958.8 | 185.6 | 0.35 | 35.92758 |
| Plant 1 Control | 9 | 1886 | 245.6 | 0.393 | 31.98269 |
| Plant 1 Control | 10 | 1389.2 | 227 | 0.339 | 37.09257 |
| Plant 1 Control | 11 | 2227.4 | 223.7 | 0.559 | 22.46641 |
| Plant 1 Control | 12 | 1871.5 | 211.08 | 0.528 | 23.80698 |
| Plant 1 Control | 13 | 1400.1 | 184.09 | 0.519 | 24.20479 |
| Plant 1 Control | 14 | 1930 | 215.33 | 0.523 | 24.02436 |
| Plant 1 Control | 15 | 1950.9 | 224.33 | 0.487 | 25.79525 |
| Plant 1 Control | 16 | 1285 | 173.09 | 0.539 | 23.31529 |
| Plant 1 Control | 17 | 1066.6 | 188.06 | 0.379 | 33.15823 |
| Plant 1 Control | 18 | 1257.9 | 201.28 | 0.39 | 32.20736 |
| Plant 1 Control | 19 | 1898.6 | 267.17 | 0.334 | 37.59602 |
| Plant 1 Control | 20 | 1538.8 | 240.28 | 0.335 | 37.51916 |
| Plant 2 Control | 1 | 1366.7 | 168.75 | 0.603 | 20.836 |
| Plant 2 Control | 2 | 1144.1 | 208.17 | 0.332 | 37.87671 |
| Plant 2 Control | 3 | 939.7 | 172.99 | 0.395 | 31.84584 |
| Plant 2 Control | 4 | 1491.7 | 219.41 | 0.389 | 32.27241 |
| Plant 2 Control | 5 | 1416 | 235.89 | 0.32 | 39.29668 |
| Plant 2 Control | 6 | 2363.8 | 240.6 | 0.513 | 24.48953 |
| Plant 2 Control | 7 | 2094 | 217.53 | 0.556 | 22.59756 |
| Plant 2 Control | 8 | 795.5 | 164.24 | 0.371 | 33.90921 |
| Plant 2 Control | 9 | 2094 | 217.53 | 0.556 | 22.59756 |
| Plant 2 Control | 10 | 2425.4 | 257.67 | 0.459 | 27.37438 |
| Plant 2 Control | 11 | 1199.6 | 230.54 | 0.284 | 44.30534 |
| Plant 2 Control | 12 | 958.4 | 151.31 | 0.526 | 23.88848 |
| Plant 2 Control | 13 | 870.7 | 144.5 | 0.524 | 23.98099 |
| Plant 2 Control | 14 | 2428.1 | 326.01 | 0.287 | 43.77189 |
| Plant 2 Control | 15 | 2685.6 | 326.46 | 0.317 | 39.68429 |
| Plant 2 Control | 17 | 913 | 151.36 | 0.501 | 25.09293 |
| Plant 3 Control | 1 | 1117.5 | 354.83 | 0.112 | 112.6661 |
| Plant 3 Control | 3 | 1099.8 | 286.78 | 0.168 | 74.77975 |
| Plant 3 Control | 4 | 881.8 | 270.93 | 0.151 | 83.24231 |
| Plant 3 Control | 5 | 2658.8 | 419.19 | 0.19 | 66.09006 |
| Plant 3 Control | 6 | 1442.8 | 280.27 | 0.231 | 54.44363 |
| Plant 3 Control | 7 | 1372 | 264.82 | 0.246 | 51.11489 |
| Plant 3 Control | 8 | 1347.7 | 270.02 | 0.232 | 54.10017 |
| Plant 3 Control | 9 | 1159.7 | 294.9 | 0.168 | 74.99009 |
| Plant 3 Control | 10 | 1175 | 281.47 | 0.186 | 67.42584 |
| Plant 3 Control | 11 | 2823.3 | 387.41 | 0.236 | 53.15996 |
| Plant 3 Control | 14 | 1904.7 | 335.72 | 0.212 | 59.17358 |
| Plant 3 Control | 15 | 1164.5 | 241.55 | 0.251 | 50.10425 |
| Plant 3 Control | 16 | 1337 | 253.48 | 0.261 | 48.05693 |
| Plant 3 Control | 17 | 1574.3 | 272.73 | 0.266 | 47.24745 |
| Plant 3 Control | 18 | 1325.3 | 260.26 | 0.246 | 51.10938 |
| Plant 3 Control | 19 | 1128 | 258.08 | 0.213 | 59.04724 |
| Plant 3 Control | 20 | 1617.2 | 299.1 | 0.227 | 55.31833 |
| Plant 1 Elevated SO_2_ | 1 | 467.1 | 119.79 | 0.409 | 30.72071 |
| Plant 1 Elevated SO_2_ | 2 | 448.2 | 118.36 | 0.402 | 31.25634 |
| Plant 1 Elevated SO_2_ | 3 | 453.2 | 112.1 | 0.453 | 27.72818 |
| Plant 1 Elevated SO_2_ | 4 | 346.8 | 108.06 | 0.373 | 33.6706 |
| Plant 1 Elevated SO_2_ | 5 | 332 | 98.33 | 0.431 | 29.12286 |
| Plant 1 Elevated SO_2_ | 7 | 329.3 | 98.97 | 0.423 | 29.7451 |
| Plant 1 Elevated SO_2_ | 8 | 340 | 102.15 | 0.409 | 30.69007 |
| Plant 1 Elevated SO_2_ | 9 | 434.3 | 111.35 | 0.44 | 28.54898 |
| Plant 1 Elevated SO_2_ | 10 | 454.6 | 113.68 | 0.442 | 28.4275 |
| Plant 2 Elevated SO_2_ | 1 | 340.3 | 90.53 | 0.522 | 24.08369 |
| Plant 2 Elevated SO_2_ | 2 | 429.1 | 106.75 | 0.473 | 26.55689 |
| Plant 2 Elevated SO_2_ | 3 | 485.4 | 113.08 | 0.477 | 26.3434 |
| Plant 2 Elevated SO_2_ | 4 | 341.7 | 96.97 | 0.457 | 27.51882 |
| Plant 2 Elevated SO_2_ | 5 | 555.3 | 120.29 | 0.482 | 26.05742 |
| Plant 3 Elevated SO_2_ | 1 | 336.1 | 123.26 | 0.278 | 45.20389 |
| Plant 3 Elevated SO_2_ | 3 | 362.2 | 116.11 | 0.338 | 37.22124 |
| Plant 3 Elevated SO_2_ | 6 | 470.2 | 124.77 | 0.38 | 33.10836 |
| Plant 1 Tr–J | 1 | 851.1 | 146.82 | 0.496 | 25.32736 |
| Plant 1 Tr–J | 2 | 835 | 172.82 | 0.351 | 35.76857 |
| Plant 1 Tr–J | 3 | 1051.9 | 166.97 | 0.474 | 26.50345 |
| Plant 1 Tr–J | 4 | 1003.8 | 136.67 | 0.675 | 18.60798 |
| Plant 1 Tr–J | 5 | 953.2 | 159.37 | 0.472 | 26.64582 |
| Plant 1 Tr–J | 6 | 851.8 | 149.73 | 0.477 | 26.31964 |
| Plant 1 Tr–J | 7 | 627.3 | 145.49 | 0.372 | 33.74357 |
| Plant 1 Tr–J | 8 | 1012.4 | 176.62 | 0.408 | 30.81255 |
| Plant 1 Tr–J | 9 | 1383.4 | 201.06 | 0.43 | 29.22157 |
| Plant 1 Tr–J | 10 | 1431.4 | 197.53 | 0.461 | 27.2587 |
| Plant 1 Tr–J | 11 | 1099.3 | 154.82 | 0.576 | 21.80409 |
| Plant 1 Tr–J | 12 | 865.6 | 128.12 | 0.663 | 18.96342 |
| Plant 1 Tr–J | 13 | 872.5 | 159.55 | 0.431 | 29.17616 |
| Plant 1 Tr–J | 14 | 999.8 | 151.88 | 0.545 | 23.07215 |
| Plant 1 Tr–J | 15 | 665.6 | 149.11 | 0.376 | 33.40413 |
| Plant 1 Tr–J | 16 | 649.9 | 136.22 | 0.44 | 28.55191 |
| Plant 1 Tr–J | 17 | 621.8 | 129.45 | 0.466 | 26.94967 |
| Plant 1 Tr–J | 18 | 833 | 144.43 | 0.502 | 25.04205 |
| Plant 1 Tr–J | 19 | 1038.9 | 161.76 | 0.499 | 25.18654 |
| Plant 1 Tr–J | 20 | 557 | 126.06 | 0.44 | 28.52984 |
| Plant 2 Tr–J | 1 | 1306.9 | 196.88 | 0.424 | 29.6593 |
| Plant 2 Tr–J | 2 | 1198.2 | 186.68 | 0.432 | 29.08481 |
| Plant 2 Tr–J | 3 | 1166.6 | 186.1 | 0.423 | 29.6873 |
| Plant 2 Tr–J | 4 | 1130.3 | 195.71 | 0.371 | 33.88694 |
| Plant 2 Tr–J | 5 | 1088.8 | 167.51 | 0.488 | 25.77112 |
| Plant 2 Tr–J | 7 | 1149.9 | 188.05 | 0.409 | 30.75294 |
| Plant 2 Tr–J | 8 | 1971.7 | 209 | 0.567 | 22.15398 |
| Plant 2 Tr–J | 9 | 1754.6 | 210.23 | 0.499 | 25.18902 |
| Plant 2 Tr–J | 10 | 1646.5 | 206.36 | 0.486 | 25.86362 |
| Plant 2 Tr–J | 11 | 1032.8 | 170.13 | 0.448 | 28.025 |
| Plant 2 Tr–J | 12 | 1973.8 | 225.23 | 0.489 | 25.70096 |
| Plant 2 Tr–J | 13 | 1406.6 | 206.34 | 0.415 | 30.26887 |
| Plant 2 Tr–J | 14 | 1786.7 | 224.23 | 0.447 | 28.14076 |
| Plant 2 Tr–J | 15 | 1759.4 | 217.45 | 0.468 | 26.87536 |
| Plant 2 Tr–J | 16 | 1396.8 | 205.06 | 0.417 | 30.10424 |
| Plant 2 Tr–J | 17 | 1525.1 | 233.2 | 0.352 | 35.65815 |
| Plant 2 Tr–J | 18 | 1276 | 212.44 | 0.355 | 35.36893 |
| Plant 2 Tr–J | 19 | 1088.8 | 173.31 | 0.456 | 27.58666 |
| Plant 2 Tr–J | 20 | 1236.9 | 206.69 | 0.364 | 34.53857 |
| Plant 2 Tr–J | 21 | 1324.6 | 208.37 | 0.383 | 32.77824 |
| Plant 3 Tr–J | 1 | 647 | 154.3 | 0.342 | 36.79828 |
| Plant 3 Tr–J | 2 | 1759.1 | 223.94 | 0.441 | 28.5084 |
| Plant 3 Tr–J | 3 | 1418.3 | 200.66 | 0.443 | 28.38922 |
| Plant 3 Tr–J | 4 | 1707.4 | 220.59 | 0.441 | 28.49944 |
| Plant 3 Tr–J | 5 | 1918.6 | 238.38 | 0.424 | 29.61796 |
| Plant 3 Tr–J | 6 | 1366.9 | 221.6 | 0.35 | 35.9255 |
| Plant 3 Tr–J | 7 | 947.4 | 171.21 | 0.406 | 30.94033 |
| Plant 3 Tr–J | 8 | 2226.2 | 255.27 | 0.429 | 29.27085 |
| Plant 3 Tr–J | 9 | 2441.8 | 270.24 | 0.42 | 29.90812 |
| Plant 3 Tr–J | 10 | 1982.1 | 257.34 | 0.376 | 33.41097 |
| Plant 3 Tr–J | 11 | 1085.6 | 173.78 | 0.452 | 27.81825 |
| Plant 3 Tr–J | 12 | 1079.9 | 177.4 | 0.431 | 29.14229 |
| Plant 3 Tr–J | 13 | 815.1 | 182.67 | 0.307 | 40.93771 |
| Plant 3 Tr–J | 14 | 1336.6 | 237.16 | 0.299 | 42.08055 |
| Plant 3 Tr–J | 15 | 951.1 | 200.36 | 0.298 | 42.20811 |
| Plant 3 Tr–J | 16 | 1314.4 | 192.54 | 0.446 | 28.20424 |
| Plant 3 Tr–J | 17 | 1366.1 | 199.93 | 0.429 | 29.25994 |
| Plant 3 Tr–J | 18 | 1038.2 | 183.3 | 0.388 | 32.36264 |
| Plant 3 Tr–J | 19 | 881.8 | 178.64 | 0.347 | 36.1899 |
| Plant 3 Tr–J | 20 | 1679.7 | 227.31 | 0.409 | 30.76135 |
