# Supplementary material for: Increased Atmospheric SO2 Detected from Changes in Leaf Physiognomy across the Triassic–Jurassic Boundary Interval of East Greenland
Source: PLoS One. 2013 Apr 10;8(4):e60614. doi: 10.1371/journal.pone.0060614 (PMC3622679; doi:10.1371/journal.pone.0060614)
Supplement: Table S3 — All measured values for each leaf analysed from the simulated palaeoatmospheric treatments in the controlled environment chambers for Lepidozamia peroffskyana. (DOC) [file pone.0060614.s003.doc]

Table S3: All measured values for each leaf analysed from the simulated palaeoatmospheric treatments in the controlled environment chambers for *Lepidozamia peroffskyana*. Gray shading indicated that the value was an outlier (over twice the standard deviation of the mean value) and was not included in analyses.

| **Treatment** | **Sample No** | **Area (mm^2^)** | **Perimeter (mm)** | **Shape factor** | **Compactness** |
| --- | --- | --- | --- | --- | --- |
| Plant 1 Control | 1 | 8.799 | 36.686 | 0.082 | 152.9563 |
| Plant 1 Control | 2 | 10.865 | 33.183 | 0.124 | 101.3448 |
| Plant 1 Control | 3 | 12.75 | 35.91 | 0.124 | 101.1395 |
| Plant 1 Control | 4 | 12.808 | 35.794 | 0.126 | 100.032 |
| Plant 1 Control | 5 | 13.268 | 37.296 | 0.12 | 104.8381 |
| Plant 1 Control | 6 | 12.413 | 39.056 | 0.102 | 122.885 |
| Plant 1 Control | 7 | 11.91 | 37.488 | 0.107 | 117.9975 |
| Plant 1 Control | 8 | 11.45 | 34.845 | 0.119 | 106.0414 |
| Plant 1 Control | 9 | 11.15 | 33.793 | 0.123 | 102.4186 |
| Plant 1 Control | 10 | 10.544 | 33.092 | 0.121 | 103.8582 |
| Plant 1 Control | 11 | 11.361 | 37.313 | 0.103 | 122.5473 |
| Plant 1 Control | 12 | 7.79 | 30.364 | 0.106 | 118.3533 |
| Plant 1 Control | 13 | 7.901 | 28.38 | 0.123 | 101.9396 |
| Plant 1 Control | 14 | 10.345 | 32.545 | 0.123 | 102.3854 |
| Plant 1 Control | 15 | 10.56 | 34.624 | 0.111 | 113.5248 |
| Plant 1 Control | 16 | 11.344 | 36.388 | 0.108 | 116.7213 |
| Plant 1 Control | 17 | 11.831 | 35.792 | 0.116 | 108.2806 |
| Plant 1 Control | 18 | 11.996 | 35.789 | 0.118 | 106.7733 |
| Plant 1 Control | 19 | 11.555 | 35.214 | 0.117 | 107.3151 |
| Plant 1 Control | 20 | 11.377 | 34.084 | 0.123 | 102.1112 |
| Plant 1 Control | 21 | 11.113 | 36.127 | 0.107 | 117.4444 |
| Plant 1 Control | 22 | 10.207 | 33.689 | 0.113 | 111.1932 |
| Plant 1 Control | 23 | 9.167 | 30.735 | 0.122 | 103.0479 |
| Plant 1 Control | 24 | 7.702 | 27.867 | 0.125 | 100.827 |
| Plant 2 Control | 1 | 10.25 | 39.448 | 0.083 | 151.819 |
| Plant 2 Control | 2 | 11.415 | 36.225 | 0.109 | 114.9584 |
| Plant 2 Control | 3 | 14.921 | 42.449 | 0.104 | 120.7639 |
| Plant 2 Control | 4 | 15.307 | 44.093 | 0.099 | 127.0133 |
| Plant 2 Control | 5 | 15.512 | 44.103 | 0.1 | 125.3916 |
| Plant 2 Control | 6 | 15.728 | 49.212 | 0.082 | 153.9815 |
| Plant 2 Control | 7 | 14.758 | 44.256 | 0.095 | 132.714 |
| Plant 2 Control | 8 | 14.18 | 41.795 | 0.102 | 123.1891 |
| Plant 2 Control | 9 | 13.109 | 39.633 | 0.105 | 119.8241 |
| Plant 2 Control | 10 | 12.157 | 38.254 | 0.104 | 120.3725 |
| Plant 2 Control | 11 | 11.427 | 39.344 | 0.093 | 135.4643 |
| Plant 2 Control | 12 | 7.563 | 29.719 | 0.108 | 116.7816 |
| Plant 2 Control | 13 | 7.693 | 29.906 | 0.108 | 116.2575 |
| Plant 2 Control | 14 | 12.499 | 40.61 | 0.095 | 131.9443 |
| Plant 2 Control | 15 | 13.977 | 43.006 | 0.095 | 132.3257 |
| Plant 2 Control | 16 | 14.714 | 49.679 | 0.075 | 167.7316 |
| Plant 2 Control | 17 | 15.048 | 44.115 | 0.097 | 129.3284 |
| Plant 2 Control | 18 | 15.837 | 44.51 | 0.1 | 125.0957 |
| Plant 2 Control | 19 | 16.633 | 45.843 | 0.099 | 126.3501 |
| Plant 2 Control | 20 | 17.301 | 46.582 | 0.1 | 125.4195 |
| Plant 2 Control | 21 | 17.362 | 47.107 | 0.098 | 127.8119 |
| Plant 2 Control | 22 | 15.745 | 45.16 | 0.097 | 129.5285 |
| Plant 2 Control | 23 | 14.541 | 43.021 | 0.099 | 127.2819 |
| Plant 2 Control | 24 | 11.534 | 39.71 | 0.092 | 136.7162 |
| Plant 3 Control | 1 | 8.136 | 29.389 | 0.118 | 106.1595 |
| Plant 3 Control | 2 | 11.127 | 33.51 | 0.125 | 100.9185 |
| Plant 3 Control | 3 | 11.432 | 34.175 | 0.123 | 102.1633 |
| Plant 3 Control | 4 | 12.527 | 35.007 | 0.128 | 97.8279 |
| Plant 3 Control | 5 | 12.529 | 34.986 | 0.129 | 97.69496 |
| Plant 3 Control | 6 | 10.161 | 33.986 | 0.111 | 113.6747 |
| Plant 3 Control | 7 | 11.004 | 34.917 | 0.113 | 110.7958 |
| Plant 3 Control | 8 | 10.587 | 34.519 | 0.112 | 112.5495 |
| Plant 3 Control | 9 | 10.785 | 33.562 | 0.12 | 104.4421 |
| Plant 3 Control | 10 | 10.088 | 31.658 | 0.126 | 99.34863 |
| Plant 3 Control | 11 | 8.731 | 31.935 | 0.108 | 116.8073 |
| Plant 3 Control | 12 | 7.762 | 29.401 | 0.113 | 111.3655 |
| Plant 3 Control | 13 | 5.491 | 23.314 | 0.127 | 98.98791 |
| Plant 3 Control | 14 | 5.793 | 24.165 | 0.125 | 100.8022 |
| Plant 3 Control | 15 | 8.588 | 33.474 | 0.096 | 130.4738 |
| Plant 3 Control | 16 | 9.664 | 34.745 | 0.101 | 124.9188 |
| Plant 3 Control | 17 | 10.109 | 34.158 | 0.109 | 115.4188 |
| Plant 3 Control | 18 | 9.497 | 32.515 | 0.113 | 111.322 |
| Plant 3 Control | 19 | 9.608 | 33.486 | 0.108 | 116.7061 |
| Plant 3 Control | 20 | 10.921 | 33.304 | 0.124 | 101.5618 |
| Plant 3 Control | 21 | 12.285 | 37.917 | 0.107 | 117.0288 |
| Plant 3 Control | 22 | 8.582 | 29.103 | 0.127 | 98.69315 |
| Plant 3 Control | 23 | 8.891 | 28.882 | 0.134 | 93.82183 |
| Plant 3 Control | 24 | 6.97 | 25.099 | 0.139 | 90.38161 |
| Plant 1 Elevated SO_2_ | 1 | 2.457 | 15.45 | 0.129 | 97.15201 |
| Plant 1 Elevated SO_2_ | 2 | 2.634 | 15.444 | 0.139 | 90.5532 |
| Plant 1 Elevated SO_2_ | 3 | 2.92 | 16.436 | 0.136 | 92.51442 |
| Plant 1 Elevated SO_2_ | 4 | 3.519 | 17.717 | 0.141 | 89.19923 |
| Plant 1 Elevated SO_2_ | 5 | 3.64 | 18.327 | 0.136 | 92.27443 |
| Plant 1 Elevated SO2 | 6 | 3.544 | 17.793 | 0.141 | 89.3315 |
| Plant 1 Elevated SO_2_ | 7 | 3.259 | 17.131 | 0.14 | 90.04945 |
| Plant 1 Elevated SO_2_ | 8 | 2.877 | 16.525 | 0.132 | 94.9168 |
| Plant 1 Elevated SO_2_ | 9 | 2.551 | 16.148 | 0.123 | 102.2179 |
| Plant 1 Elevated SO_2_ | 10 | 1.737 | 12.178 | 0.147 | 85.37921 |
| Plant 1 Elevated SO_2_ | 11 | 1.506 | 11.399 | 0.146 | 86.27968 |
| Plant 1 Elevated SO_2_ | 12 | 2.456 | 15.043 | 0.136 | 92.13838 |
| Plant 1 Elevated SO_2_ | 13 | 2.884 | 16.443 | 0.134 | 93.74905 |
| Plant 1 Elevated SO_2_ | 14 | 3.195 | 17.778 | 0.127 | 98.92247 |
| Plant 1 Elevated SO_2_ | 15 | 3.444 | 19.465 | 0.114 | 110.0134 |
| Plant 1 Elevated SO_2_ | 16 | 3.419 | 18.084 | 0.131 | 95.65108 |
| Plant 1 Elevated SO_2_ | 17 | 3.648 | 18.307 | 0.137 | 91.87123 |
| Plant 1 Elevated SO_2_ | 18 | 3.089 | 17.082 | 0.133 | 94.46252 |
| Plant 1 Elevated SO_2_ | 19 | 2.501 | 15.024 | 0.139 | 90.25213 |
| Plant 1 Elevated SO_2_ | 20 | 1.567 | 12.659 | 0.123 | 102.2657 |
| Plant 1 Elevated SO_2_ | 21 | 1.457 | 10.785 | 0.157 | 79.83269 |
| Plant 2 Elevated SO_2_ | 1 | 5.539 | 22.966 | 0.132 | 95.22245 |
| Plant 2 Elevated SO2 | 2 | 5.553 | 23.38 | 0.128 | 98.43767 |
| Plant 2 Elevated SO2 | 3 | 6.74 | 25.091 | 0.135 | 93.40627 |
| Plant 2 Elevated SO2 | 4 | 6.799 | 25.628 | 0.13 | 96.60162 |
| Plant 2 Elevated SO_2_ | 5 | 6.964 | 26.484 | 0.125 | 100.7183 |
| Plant 2 Elevated SO_2_ | 6 | 6.986 | 26.715 | 0.123 | 102.1602 |
| Plant 2 Elevated SO_2_ | 7 | 6.721 | 26.021 | 0.125 | 100.7428 |
| Plant 2 Elevated SO_2_ | 8 | 5.826 | 25.782 | 0.11 | 114.094 |
| Plant 2 Elevated SO_2_ | 9 | 5.511 | 24.518 | 0.115 | 109.0786 |
| Plant 2 Elevated SO_2_ | 10 | 5.336 | 23.211 | 0.124 | 100.9652 |
| Plant 2 Elevated SO_2_ | 11 | 4.346 | 20.21 | 0.134 | 93.98162 |
| Plant 2 Elevated SO_2_ | 12 | 2.194 | 14.421 | 0.133 | 94.78817 |
| Plant 2 Elevated SO_2_ | 13 | 2.503 | 14.522 | 0.149 | 84.25429 |
| Plant 2 Elevated SO_2_ | 14 | 4.169 | 19.448 | 0.139 | 90.72312 |
| Plant 2 Elevated SO_2_ | 15 | 5.129 | 22.247 | 0.13 | 96.4962 |
| Plant 2 Elevated SO_2_ | 16 | 5.401 | 23.89 | 0.119 | 105.6716 |
| Plant 2 Elevated SO_2_ | 17 | 5.613 | 24.303 | 0.119 | 105.2264 |
| Plant 2 Elevated SO_2_ | 18 | 5.829 | 24.348 | 0.124 | 101.7027 |
| Plant 2 Elevated SO_2_ | 19 | 6.156 | 25.202 | 0.122 | 103.1743 |
| Plant 2 Elevated SO_2_ | 20 | 6.703 | 25.642 | 0.128 | 98.09222 |
| Plant 2 Elevated SO_2_ | 21 | 6.388 | 23.809 | 0.142 | 88.73959 |
| Plant 2 Elevated SO_2_ | 22 | 5.859 | 24.323 | 0.124 | 100.9743 |
| Plant 3 Elevated SO_2_ | 1 | 1.979 | 14.42 | 0.12 | 105.0715 |
| Plant 3 Elevated SO_2_ | 2 | 2.371 | 15.887 | 0.118 | 106.4516 |
| Plant 3 Elevated SO_2_ | 3 | 3.091 | 17.077 | 0.133 | 94.34614 |
| Plant 3 Elevated SO_2_ | 4 | 3.434 | 17.972 | 0.134 | 94.0573 |
| Plant 3 Elevated SO_2_ | 5 | 3.489 | 17.306 | 0.146 | 85.84054 |
| Plant 3 Elevated SO_2_ | 6 | 2.852 | 15.614 | 0.147 | 85.48282 |
| Plant 3 Elevated SO_2_ | 7 | 2.525 | 14.507 | 0.151 | 83.34774 |
| Plant 3 Elevated SO_2_ | 8 | 2.259 | 14.433 | 0.136 | 92.21403 |
| Plant 3 Elevated SO_2_ | 9 | 1.124 | 9.211 | 0.167 | 75.48267 |
| Plant 3 Elevated SO_2_ | 10 | 0.893 | 7.442 | 0.203 | 62.01944 |
| Plant 3 Elevated SO_2_ | 11 | 1.815 | 11.744 | 0.165 | 75.98983 |
| Plant 3 Elevated SO_2_ | 12 | 2.634 | 14.966 | 0.148 | 85.03461 |
| Plant 3 Elevated SO_2_ | 13 | 3.115 | 16.567 | 0.143 | 88.11091 |
| Plant 3 Elevated SO_2_ | 14 | 3.719 | 19.636 | 0.121 | 103.6764 |
| Plant 3 Elevated SO_2_ | 15 | 3.903 | 18.888 | 0.137 | 91.40572 |
| Plant 3 Elevated SO_2_ | 16 | 3.921 | 19.122 | 0.135 | 93.2545 |
| Plant 3 Elevated SO_2_ | 17 | 3.44 | 17.479 | 0.142 | 88.81263 |
| Plant 3 Elevated SO_2_ | 18 | 2.959 | 15.987 | 0.145 | 86.37518 |
| Plant 3 Elevated SO_2_ | 19 | 2.197 | 14.232 | 0.136 | 92.19382 |
| Plant 1 Tr–J | 1 | z | 21.385 | 0.105 | 119.1864 |
| Plant 1 Tr–J | 2 | 4.536 | 21.707 | 0.121 | 103.8787 |
| Plant 1 TR–J | 3 | 5.795 | 24.371 | 0.123 | 102.4928 |
| Plant 1 TR–J | 4 | 6.931 | 26.727 | 0.122 | 103.0634 |
| Plant 1 TR–J | 5 | 7.597 | 29.065 | 0.113 | 111.1984 |
| Plant 1 TR–J | 6 | 8.326 | 30.438 | 0.113 | 111.2745 |
| Plant 1 TR–J | 7 | 9.208 | 32.099 | 0.112 | 111.8968 |
| Plant 1 TR–J | 8 | 9.338 | 33.041 | 0.107 | 116.9102 |
| Plant 1 TR–J | 9 | 8.214 | 30.797 | 0.109 | 115.4681 |
| Plant 1 TR–J | 10 | 6.507 | 26.286 | 0.118 | 106.1862 |
| Plant 1 TR–J | 11 | 4.284 | 21.214 | 0.12 | 105.0499 |
| Plant 1 TR–J | 12 | 4.214 | 21.085 | 0.119 | 105.5001 |
| Plant 1 TR–J | 13 | 7.568 | 27.949 | 0.122 | 103.217 |
| Plant 1 TR–J | 14 | 8.834 | 31.142 | 0.114 | 109.7831 |
| Plant 1 TR–J | 15 | 10.232 | 34.763 | 0.106 | 118.1065 |
| Plant 1 TR–J | 16 | 10.097 | 35.319 | 0.102 | 123.5448 |
| Plant 1 TR–J | 17 | 9.984 | 33.992 | 0.109 | 115.7308 |
| Plant 1 TR–J | 18 | 9.114 | 31.272 | 0.117 | 107.3006 |
| Plant 1 TR–J | 19 | 8.043 | 27.571 | 0.133 | 94.512 |
| Plant 1 TR–J | 20 | 6.739 | 24.481 | 0.141 | 88.93298 |
| Plant 1 TR–J | 21 | 5.606 | 21.644 | 0.15 | 83.56453 |
| Plant 2 TR–J | 1 | 5.348 | 23.341 | 0.123 | 101.8703 |
| Plant 2 TR–J | 2 | 6.392 | 25.385 | 0.125 | 100.8132 |
| Plant 2 TR–J | 3 | 6.887 | 26.798 | 0.121 | 104.2737 |
| Plant 2 TR–J | 4 | 7.075 | 27.745 | 0.115 | 108.8035 |
| Plant 2 TR–J | 5 | 7.569 | 29.273 | 0.111 | 113.2129 |
| Plant 2 TR–J | 6 | 7.805 | 30.833 | 0.103 | 121.8032 |
| Plant 2 TR–J | 7 | 7.724 | 30.813 | 0.102 | 122.9209 |
| Plant 2 TR–J | 8 | 9.006 | 36.048 | 0.087 | 144.2881 |
| Plant 2 TR–J | 9 | 7.89 | 30.51 | 0.107 | 117.9797 |
| Plant 2 TR–J | 10 | 7.483 | 30.025 | 0.104 | 120.4732 |
| Plant 2 TR–J | 11 | 7.023 | 28.263 | 0.11 | 113.7402 |
| Plant 2 TR–J | 12 | 7.07 | 28.136 | 0.112 | 111.9709 |
| Plant 2 TR–J | 13 | 6.611 | 27.63 | 0.109 | 115.4768 |
| Plant 2 TR–J | 14 | 6.259 | 26.23 | 0.114 | 109.9238 |
| Plant 2 TR–J | 15 | 5.495 | 25.824 | 0.104 | 121.3611 |
| Plant 2 TR–J | 16 | 4.635 | 22.826 | 0.112 | 112.4113 |
| Plant 2 TR–J | 17 | 3.998 | 21.019 | 0.114 | 110.5048 |
| Plant 2 TR–J | 18 | 3.302 | 19.111 | 0.114 | 110.6088 |
| Plant 2 TR–J | 19 | 2.707 | 16.815 | 0.12 | 104.4493 |
| Plant 2 TR–J | 20 | 4.634 | 22.773 | 0.112 | 111.914 |
| Plant 2 TR–J | 21 | 5.367 | 24.278 | 0.114 | 109.8232 |
| Plant 2 TR–J | 22 | 6.397 | 28.472 | 0.099 | 126.7242 |
| Plant 2 TR–J | 23 | 6.862 | 29.082 | 0.102 | 123.2531 |
| Plant 2 TR–J | 24 | 7.077 | 29.261 | 0.104 | 120.9843 |
| Plant 2 TR–J | 25 | 6.961 | 29.142 | 0.103 | 122.002 |
| Plant 2 TR–J | 26 | 7.909 | 30.615 | 0.106 | 118.5078 |
| Plant 2 TR–J | 27 | 8.446 | 30.677 | 0.113 | 111.423 |
| Plant 2 TR–J | 28 | 8.782 | 32.768 | 0.103 | 122.2662 |
| Plant 2 TR–J | 29 | 8.54 | 32.313 | 0.103 | 122.2635 |
| Plant 2 TR–J | 30 | 7.994 | 30.876 | 0.105 | 119.2554 |
| Plant 2 TR–J | 31 | 7.819 | 30.035 | 0.109 | 115.373 |
| Plant 2 TR–J | 32 | 7.086 | 28.039 | 0.113 | 110.9491 |
| Plant 2 TR–J | 33 | 6.789 | 26.12 | 0.125 | 100.4941 |
| Plant 2 TR–J | 34 | 6.171 | 24.653 | 0.128 | 98.48816 |
| Plant 2 TR–J | 35 | 5.151 | 22.11 | 0.132 | 94.90431 |
| Plant 3 TR–J | 1 | 3.436 | 18.475 | 0.126 | 99.33807 |
| Plant 3 TR–J | 2 | 3.99 | 18.437 | 0.148 | 85.19373 |
| Plant 3 TR–J | 3 | 4.136 | 19.251 | 0.14 | 89.60372 |
| Plant 3 TR–J | 4 | 4.752 | 20.119 | 0.148 | 85.17975 |
| Plant 3 TR–J | 5 | 4.612 | 20.574 | 0.137 | 91.78003 |
| Plant 3 TR–J | 6 | 4.721 | 20.368 | 0.143 | 87.87448 |
| Plant 3 TR–J | 7 | 4.352 | 19.723 | 0.141 | 89.38344 |
| Plant 3 TR–J | 8 | 5.238 | 22.076 | 0.135 | 93.04119 |
| Plant 3 TR–J | 9 | 4.353 | 19.902 | 0.138 | 90.99233 |
| Plant 3 TR–J | 10 | 2.975 | 16.044 | 0.145 | 86.52435 |
| Plant 3 TR–J | 11 | 1.74 | 11.499 | 0.165 | 75.99253 |
| Plant 3 TR–J | 12 | 1.952 | 12.403 | 0.159 | 78.80861 |
| Plant 3 TR–J | 13 | 3.476 | 16.914 | 0.153 | 82.30247 |
| Plant 3 TR–J | 14 | 4.827 | 20.074 | 0.151 | 83.48156 |
| Plant 3 TR–J | 15 | 5.624 | 21.709 | 0.15 | 83.79813 |
| Plant 3 TR–J | 16 | 6.318 | 24.524 | 0.132 | 95.19256 |
| Plant 3 TR–J | 17 | 5.358 | 22.135 | 0.137 | 91.44424 |
| Plant 3 TR–J | 18 | 5.444 | 21.935 | 0.142 | 88.38064 |
| Plant 3 TR–J | 19 | 4.996 | 20.501 | 0.149 | 84.1255 |
| Plant 3 TR–J | 20 | 4.97 | 20.616 | 0.147 | 85.51699 |
| Plant 3 TR–J | 21 | 4.165 | 18.604 | 0.151 | 83.09936 |
| Plant 3 TR–J | 22 | 3.502 | 16.797 | 0.156 | 80.56517 |
