# Supplementary material for: Increased Atmospheric SO2 Detected from Changes in Leaf Physiognomy across the Triassic–Jurassic Boundary Interval of East Greenland
Source: PLoS One. 2013 Apr 10;8(4):e60614. doi: 10.1371/journal.pone.0060614 (PMC3622679; doi:10.1371/journal.pone.0060614)
Supplement: Table S4 — All measured values for each leaf analysed from the simulated palaeoatmospheric treatments in the controlled environment chambers for Lepidozamia hopei. (DOC) [file pone.0060614.s004.doc]

Table S4: All measured values for each leaf analysed from the simulated palaeoatmospheric treatments in the controlled environment chambers for *Lepidozamia hopei*. Gray shading indicated that the value was an outlier (over twice the standard deviation of the mean value) and was not included in analyses.

| **Treatment** | **Sample No** | **Area (mm^2^)** | **Perimeter (mm)** | **Shape factor** | **Compactness** |
| --- | --- | --- | --- | --- | --- |
| Plant 1 Control | 1 | 29.546 | 54.215 | 0.126 | 99.48102 |
| Plant 1 Control | 2 | 31.525 | 57.131 | 0.121 | 103.5353 |
| Plant 1 Control | 3 | 36.465 | 58.76 | 0.133 | 94.68635 |
| Plant 1 Control | 4 | 37.352 | 60.323 | 0.129 | 97.42087 |
| Plant 1 Control | 5 | 29.02 | 56.169 | 0.116 | 108.7166 |
| Plant 1 Control | 6 | 33.54 | 60.939 | 0.113 | 110.7204 |
| Plant 1 Control | 7 | 34.381 | 62.636 | 0.11 | 114.1115 |
| Plant 1 Control | 8 | 36.076 | 59.968 | 0.126 | 99.68292 |
| Plant 1 Control | 10 | 26.578 | 49.636 | 0.136 | 92.69819 |
| Plant 1 Control | 11 | 27.029 | 47.809 | 0.149 | 84.56474 |
| Plant 1 Control | 12 | 22.695 | 40.626 | 0.173 | 72.72403 |
| Plant 1 Control | 13 | 17.315 | 40.307 | 0.134 | 93.8293 |
| Plant 1 Control | 14 | 8.871 | 26.156 | 0.163 | 77.12054 |
| Plant 1 Control | 15 | 7.723 | 25.187 | 0.153 | 82.1423 |
| Plant 1 Control | 16 | 12.639 | 31.52 | 0.16 | 78.60673 |
| Plant 1 Control | 17 | 19.262 | 37.533 | 0.172 | 73.13499 |
| Plant 1 Control | 18 | 22.943 | 42.345 | 0.161 | 78.15451 |
| Plant 1 Control | 19 | 25.684 | 47.407 | 0.144 | 87.50287 |
| Plant 1 Control | 20 | 28.998 | 50.774 | 0.141 | 88.90265 |
| Plant 1 Control | 21 | 35.018 | 57.573 | 0.133 | 94.65562 |
| Plant 1 Control | 22 | 36.717 | 59.385 | 0.131 | 96.04756 |
| Plant 1 Control | 23 | 29.598 | 53.756 | 0.129 | 97.63185 |
| Plant 1 Control | 24 | 33.973 | 58.798 | 0.123 | 101.7633 |
| Plant 1 Control | 25 | 38.46 | 60.894 | 0.13 | 96.41392 |
| Plant 1 Control | 26 | 40.202 | 60.616 | 0.137 | 91.39594 |
| Plant 1 Control | 27 | 31.063 | 54.102 | 0.133 | 94.22871 |
| Plant 1 Control | 28 | 33.547 | 56.53 | 0.132 | 95.25862 |
| Plant 1 Control | 29 | 35.184 | 57.473 | 0.134 | 93.88204 |
| Plant 2 Control | 1 | 29.446 | 49.924 | 0.148 | 84.64327 |
| Plant 2 Control | 2 | 39.039 | 55.918 | 0.157 | 80.09485 |
| Plant 2 Control | 3 | 42.101 | 59.036 | 0.152 | 82.78305 |
| Plant 2 Control | 4 | 48.074 | 61.653 | 0.159 | 79.06753 |
| Plant 2 Control | 5 | 31.37 | 52.009 | 0.146 | 86.22684 |
| Plant 2 Control | 6 | 27.542 | 47.435 | 0.154 | 81.69629 |
| Plant 2 Control | 7 | 16.557 | 34.464 | 0.175 | 71.73807 |
| Plant 2 Control | 8 | 21.191 | 38.619 | 0.179 | 70.38022 |
| Plant 2 Control | 9 | 26.667 | 48.32 | 0.144 | 87.55475 |
| Plant 2 Control | 10 | 37.795 | 56.682 | 0.148 | 85.00725 |
| Plant 2 Control | 12 | 34.502 | 53.45 | 0.152 | 82.80397 |
| Plant 2 Control | 13 | 37.387 | 55.907 | 0.15 | 83.60106 |
| Plant 2 Control | 14 | 40.438 | 54.856 | 0.169 | 74.41468 |
| Plant 2 Control | 15 | 37.612 | 53.267 | 0.167 | 75.43798 |
| Plant 3 Control | 2 | 20.707 | 40.303 | 0.16 | 78.44361 |
| Plant 3 Control | 3 | 16.358 | 34.335 | 0.174 | 72.06824 |
| Plant 3 Control | 4 | 22.129 | 43.199 | 0.149 | 84.33068 |
| Plant 3 Control | 5 | 19.707 | 39.614 | 0.158 | 79.63003 |
| Plant 3 Control | 6 | 18.487 | 38.359 | 0.158 | 79.59176 |
| Plant 3 Control | 7 | 21.33 | 44.08 | 0.138 | 91.09453 |
| Plant 3 Control | 8 | 15.449 | 36.497 | 0.146 | 86.22118 |
| Plant 3 Control | 9 | 2.693 | 11.588 | 0.252 | 49.86325 |
| Plant 3 Control | 10 | 7.603 | 25.008 | 0.153 | 82.25701 |
| Plant 3 Control | 11 | 12.179 | 31.045 | 0.159 | 79.13556 |
| Plant 3 Control | 12 | 22.515 | 46.053 | 0.133 | 94.19848 |
| Plant 3 Control | 13 | 24.675 | 47.073 | 0.14 | 89.80212 |
| Plant 3 Control | 14 | 18.687 | 42.978 | 0.127 | 98.84457 |
| Plant 3 Control | 15 | 21.079 | 39.025 | 0.174 | 72.24966 |
| Plant 3 Control | 16 | 17.389 | 33.013 | 0.201 | 62.67515 |
| Plant 3 Control | 17 | 15.358 | 33.279 | 0.174 | 72.11172 |
| Plant 1 Elevated SO_2_ | 1 | 7.581 | 23.099 | 0.179 | 70.38172 |
| Plant 1 Elevated SO_2_ | 2 | 11.625 | 28.585 | 0.179 | 70.28836 |
| Plant 1 Elevated SO_2_ | 3 | 12.263 | 29.703 | 0.175 | 71.94554 |
| Plant 1 Elevated SO_2_ | 4 | 11.101 | 28.924 | 0.167 | 75.36238 |
| Plant 1 Elevated SO_2_ | 5 | 10.038 | 28.591 | 0.154 | 81.43507 |
| Plant 1 Elevated SO_2_ | 6 | 5.891 | 20.59 | 0.175 | 71.96539 |
| Plant 1 Elevated SO_2_ | 7 | 7.437 | 22.078 | 0.192 | 65.5423 |
| Plant 1 Elevated SO_2_ | 9 | 13.606 | 31.755 | 0.17 | 74.11289 |
| Plant 1 Elevated SO_2_ | 10 | 13.734 | 30.882 | 0.181 | 69.44065 |
| Plant 2 Elevated SO_2_ | 1 | 12.478 | 30.835 | 0.165 | 76.19789 |
| Plant 2 Elevated SO_2_ | 2 | 12.151 | 29.19 | 0.179 | 70.1223 |
| Plant 2 Elevated SO_2_ | 3 | 11.106 | 27.276 | 0.188 | 66.98903 |
| Plant 2 Elevated SO_2_ | 4 | 7.054 | 19.828 | 0.225 | 55.73428 |
| Plant 2 Elevated SO_2_ | 5 | 9.656 | 22.276 | 0.245 | 51.38983 |
| Plant 2 Elevated SO_2_ | 6 | 13.781 | 29.401 | 0.2 | 62.7254 |
| Plant 2 Elevated SO_2_ | 7 | 15.179 | 32.078 | 0.185 | 67.7909 |
| Plant 2 Elevated SO_2_ | 8 | 13.708 | 30.416 | 0.186 | 67.48855 |
| Plant 2 Elevated SO_2_ | 1 | 15.365 | 33.381 | 0.173 | 72.52139 |
| Plant 3 Elevated SO_2_ | 2 | 16.755 | 33.204 | 0.191 | 65.80159 |
| Plant 3 Elevated SO_2_ | 3 | 16.269 | 34.771 | 0.169 | 74.31449 |
| Plant 3 Elevated SO_2_ | 4 | 14.242 | 30.938 | 0.187 | 67.20684 |
| Plant 3 Elevated SO_2_ | 5 | 7.94 | 23.559 | 0.18 | 69.90258 |
| Plant 3 Elevated SO_2_ | 6 | 12.474 | 31.854 | 0.154 | 81.34338 |
| Plant 3 Elevated SO_2_ | 7 | 16.302 | 34.201 | 0.175 | 71.75245 |
| Plant 3 Elevated SO_2_ | 8 | 18.109 | 34.51 | 0.191 | 65.76509 |
| Plant 3 Elevated SO_2_ | 9 | 17.719 | 33.467 | 0.199 | 63.21125 |
| Plant 3 Elevated SO_2_ | 10 | 13.901 | 29.757 | 0.197 | 63.69895 |
| Plant 1 Tr–J | 1 | 13.127 | 35.764 | 0.129 | 97.43762 |
| Plant 1 TR–J | 2 | 18.145 | 42.533 | 0.126 | 99.69998 |
| Plant 1 TR–J | 3 | 22.233 | 46.366 | 0.13 | 96.69437 |
| Plant 1 TR–J | 4 | 26.63 | 49.397 | 0.137 | 91.62837 |
| Plant 1 TR–J | 5 | 24.986 | 47.737 | 0.138 | 91.20392 |
| Plant 1 TR–J | 6 | 26.235 | 47.889 | 0.144 | 87.41591 |
| Plant 1 TR–J | 7 | 27.827 | 47.9 | 0.152 | 82.45265 |
| Plant 1 TR–J | 8 | 26.039 | 47.458 | 0.145 | 86.49571 |
| Plant 1 TR–J | 9 | 19.235 | 40.159 | 0.15 | 83.84431 |
| Plant 1 TR–J | 10 | 15.344 | 33.415 | 0.173 | 72.76865 |
| Plant 1 TR–J | 11 | 11.871 | 29.449 | 0.172 | 73.05565 |
| Plant 1 TR–J | 12 | 21.458 | 39.558 | 0.172 | 72.9255 |
| Plant 1 TR–J | 13 | 27.323 | 44.768 | 0.171 | 73.35116 |
| Plant 1 TR–J | 14 | 31.38 | 49.279 | 0.162 | 77.3875 |
| Plant 1 TR–J | 15 | 35.74 | 53.555 | 0.157 | 80.25008 |
| Plant 1 TR–J | 16 | 30.372 | 51.612 | 0.143 | 87.70573 |
| Plant 1 TR–J | 18 | 35.228 | 57.111 | 0.136 | 92.58733 |
| Plant 1 TR–J | 19 | 28.683 | 51.31 | 0.137 | 91.78664 |
| Plant 1 TR–J | 20 | 24.91 | 47.546 | 0.138 | 90.75159 |
| Plant 1 TR–J | 21 | 14.314 | 37.035 | 0.131 | 95.82166 |
| Plant 2 TR–J | 1 | 11.473 | 33.244 | 0.13 | 96.32734 |
| Plant 2 TR–J | 4 | 22.153 | 49.408 | 0.114 | 110.195 |
| Plant 2 TR–J | 5 | 20.178 | 45.922 | 0.12 | 104.5114 |
| Plant 2 TR–J | 6 | 17.048 | 41.446 | 0.125 | 100.7608 |
| Plant 2 TR–J | 7 | 14.239 | 35.713 | 0.14 | 89.57219 |
| Plant 2 TR–J | 8 | 13.069 | 32.67 | 0.154 | 81.66875 |
| Plant 2 TR–J | 9 | 18.627 | 41.935 | 0.133 | 94.40834 |
| Plant 2 TR–J | 10 | 22.661 | 49.198 | 0.118 | 106.811 |
| Plant 2 TR–J | 11 | 20.731 | 47.148 | 0.117 | 107.2275 |
| Plant 2 TR–J | 12 | 21.682 | 46.868 | 0.124 | 101.3103 |
| Plant 2 TR–J | 13 | 19.191 | 43.165 | 0.129 | 97.08807 |
| Plant 2 TR–J | 1 | 6.206 | 24.907 | 0.126 | 99.96111 |
| Plant 2 TR–J | 2 | 10.267 | 27.359 | 0.172 | 72.90493 |
| Plant 2 TR–J | 3 | 11.783 | 28.812 | 0.178 | 70.45161 |
| Plant 2 TR–J | 4 | 12.595 | 29.905 | 0.177 | 71.00508 |
| Plant 2 TR–J | 5 | 12.837 | 30.333 | 0.175 | 71.67492 |
| Plant 2 TR–J | 6 | 11.576 | 29.392 | 0.168 | 74.62765 |
| Plant 2 TR–J | 7 | 14.157 | 37.192 | 0.129 | 97.70748 |
| Plant 2 TR–J | 8 | 13.714 | 32.957 | 0.159 | 79.2011 |
| Plant 2 TR–J | 9 | 10.647 | 28.019 | 0.17 | 73.73573 |
| Plant 2 TR–J | 10 | 6.512 | 21.624 | 0.175 | 71.80549 |
| Plant 2 TR–J | 11 | 7.664 | 21.762 | 0.203 | 61.7934 |
| Plant 2 TR–J | 12 | 11.282 | 27.531 | 0.187 | 67.18277 |
| Plant 2 TR–J | 13 | 12.951 | 33.818 | 0.142 | 88.30647 |
| Plant 2 TR–J | 14 | 13.119 | 31.86 | 0.162 | 77.37324 |
| Plant 2 TR–J | 15 | 13.142 | 31.589 | 0.166 | 75.92946 |
| Plant 2 TR–J | 16 | 13.219 | 31.023 | 0.173 | 72.8063 |
| Plant 2 TR–J | 17 | 14.098 | 31.424 | 0.179 | 70.04311 |
| Plant 2 TR–J | 18 | 12.91 | 30.37 | 0.176 | 71.4436 |
