# Supplementary material for: Increased Atmospheric SO2 Detected from Changes in Leaf Physiognomy across the Triassic–Jurassic Boundary Interval of East Greenland
Source: PLoS One. 2013 Apr 10;8(4):e60614. doi: 10.1371/journal.pone.0060614 (PMC3622679; doi:10.1371/journal.pone.0060614)
Supplement: Table S5 — All measured values for each leaf analysed from the simulated palaeoatmospheric treatments in the controlled environment chambers for Ginkgo biloba. (DOC) [file pone.0060614.s005.doc]

Table S5: All measured values for each leaf analysed from the simulated palaeoatmospheric treatments in the controlled environment chambers for *Ginkgo biloba*. Gray shading indicated that the value was an outlier (over twice the standard deviation of the mean value) and was not included in analyses.

| **Treatment** | **Sample No** | **Area (mm^2^)** | **Perimeter (mm)** | **Shape Factor** | **Compactness** |
| --- | --- | --- | --- | --- | --- |
| Plant 1 Control | S 1 | 2753.2 | 350.79 | 0.281 | 44.69476 |
| Plant 1 Control | S 2 | 3312.7 | 345.58 | 0.349 | 36.05082 |
| Plant 1 Control | S 3 | 11264.8 | 778.38 | 0.234 | 53.78484 |
| Plant 1 Control | S 4 | 8314.7 | 656.47 | 0.242 | 51.83024 |
| Plant 1 Control | S 5 | 3994.4 | 537.93 | 0.173 | 72.44359 |
| Plant 1 Control | R 21 | 8048.6 | 737.6 | 0.186 | 67.59607 |
| Plant 1 Control | R 17 | 14454.1 | 793.9 | 0.288 | 43.60543 |
| Plant 1 Control | R 7 | 3721.9 | 419.61 | 0.266 | 47.30717 |
| Plant 1 Control | R 9 | 3487.5 | 390.86 | 0.287 | 43.80546 |
| Plant 1 Control | R 8 | 2001.9 | 326.59 | 0.236 | 53.2799 |
| Plant 1 Control | R 19 | 365.6 | 113.72 | 0.355 | 35.37264 |
| Plant 1 Control | R 11 | 1393.8 | 258.86 | 0.261 | 48.07612 |
| Plant 1 Control | R 44 | 1460.6 | 323.79 | 0.175 | 71.7787 |
| Plant 1 Control | R 42 | 2005.5 | 393.21 | 0.163 | 77.09504 |
| Plant 1 Control | R 24 | 554.5 | 126.9 | 0.433 | 29.04168 |
| Plant 1 Control | R 22 | 423.5 | 117.82 | 0.383 | 32.77816 |
| Plant 1 Control | R 35 | 702 | 178.58 | 0.277 | 45.42851 |
| Plant 1 Control | R 23 | 345.5 | 111.59 | 0.349 | 36.04147 |
| Plant 1 Control | R 30 | 479.7 | 126.62 | 0.376 | 33.42219 |
| Plant 1 Control | R 33 | 476.1 | 147.14 | 0.276 | 45.47402 |
| Plant 2 Control | S 1 | 788.8 | 162.92 | 0.373 | 33.64975 |
| Plant 2 Control | S 2 | 1386.3 | 219.27 | 0.362 | 34.68177 |
| Plant 2 Control | S 3 | 1208.2 | 196.09 | 0.395 | 31.82527 |
| Plant 2 Control | S 4 | 2221.6 | 307.03 | 0.296 | 42.43222 |
| Plant 2 Control | S 5 | 2174.6 | 293.78 | 0.317 | 39.68854 |
| Plant 2 Control | R 51 | 3773.1 | 392.76 | 0.307 | 40.88426 |
| Plant 2 Control | R 60 | 1367 | 249.97 | 0.275 | 45.70958 |
| Plant 2 Control | R 57 | 2102.6 | 292.43 | 0.309 | 40.67122 |
| Plant 2 Control | R 88 | 5551.2 | 707.63 | 0.139 | 90.20396 |
| Plant 2 Control | R 50 | 1081.8 | 209 | 0.311 | 40.37807 |
| Plant 2 Control | R 13 | 573 | 136.86 | 0.384 | 32.68876 |
| Plant 2 Control | R 84 | 10732.2 | 926.29 | 0.157 | 79.94756 |
| Plant 2 Control | R 83 | 3042.3 | 392.13 | 0.249 | 50.54266 |
| Plant 2 Control | R 31 | 1311.5 | 246.09 | 0.272 | 46.17635 |
| Plant 2 Control | R 75 | 2018.5 | 281.61 | 0.32 | 39.28868 |
| Plant 2 Control | R 76 | 1498.1 | 233.14 | 0.346 | 36.28213 |
| Plant 2 Control | R 47 | 1818.6 | 287.33 | 0.277 | 45.39675 |
| Plant 2 Control | R 3 | 401.3 | 110.02 | 0.417 | 30.16297 |
| Plant 2 Control | R 43 | 4001.1 | 374.08 | 0.359 | 34.97434 |
| Plant 3 Control | S 1 | 1291.7 | 191.98 | 0.44 | 28.53319 |
| Plant 3 Control | S 2 | 2050.5 | 277.97 | 0.333 | 37.68219 |
| Plant 3 Control | S 3 | 2481.8 | 262.05 | 0.454 | 27.66952 |
| Plant 3 Control | S 4 | 5466 | 504.64 | 0.27 | 46.59011 |
| Plant 3 Control | S 5 | 4613.6 | 397.19 | 0.367 | 34.19453 |
| Plant 3 Control | R 59 | 4992.6 | 407.67 | 0.378 | 33.28823 |
| Plant 3 Control | R 68 | 1418.7 | 266.01 | 0.252 | 49.87758 |
| Plant 3 Control | R 26 | 2629.9 | 325.44 | 0.31188 | 40.27195 |
| Plant 3 Control | R 20 | 3113.5 | 307.75 | 0.413 | 30.41916 |
| Plant 3 Control | R 21 | 2427 | 274.75 | 0.404 | 31.10324 |
| Plant 3 Control | R 33 | 675.7 | 153.44 | 0.361 | 34.84362 |
| Plant 3 Control | R 43 | 3573.2 | 364.41 | 0.338 | 37.16407 |
| Plant 3 Control | R 56 | 4343.6 | 448.07 | 0.272 | 46.22127 |
| Plant 3 Control | R 35 | 2568.2 | 299.47 | 0.36 | 34.92029 |
| Plant 3 Control | R 10 | 3972.5 | 382.42 | 0.341 | 36.81436 |
| Plant 3 Control | R 7 | 1899.3 | 237.89 | 0.422 | 29.79606 |
| Plant 3 Control | R 38 | 3180.8 | 321.28 | 0.387 | 32.45122 |
| Plant 3 Control | R 2 | 1063.7 | 169.86 | 0.463 | 27.12458 |
| Plant 3 Control | R 40 | 1667.6 | 237.16 | 0.373 | 33.72803 |
| Plant 3 Control | R 23 | 946.3 | 202.79 | 0.289 | 43.45745 |
| Plant 1 Elevated SO_2_ | 1 | 421.6 | 107.22 | 0.461 | 27.26786 |
| Plant 1 Elevated SO_2_ | 2 | 236.7 | 66.01 | 0.683 | 18.40862 |
| Plant 1 Elevated SO_2_ | 3 | 200.2 | 66.79 | 0.564 | 22.28224 |
| Plant 1 Elevated SO_2_ | 4 | 399.8 | 114.19 | 0.385 | 32.6147 |
| Plant 1 Elevated SO_2_ | 5 | 318 | 81.53 | 0.601 | 20.90296 |
| Plant 2 Elevated SO_2_ | 1 | 440.3 | 108.05 | 0.474 | 26.51556 |
| Plant 2 Elevated SO_2_ | 2 | 497.5 | 121.42 | 0.424 | 29.6338 |
| Plant 2 Elevated SO_2_ | 3 | 404.7 | 116.98 | 0.372 | 33.81349 |
| Plant 2 Elevated SO_2_ | 4 | 385.6 | 112.52 | 0.383 | 32.8339 |
| Plant 1 TR–J | 1 | 712.7 | 128.13 | 0.545249 | 23.03535 |
| Plant 1 TR–J | 2 | 517.6 | 128.31 | 0.394878 | 31.8073 |
| Plant 1 TR–J | 3 | 522.1 | 104.88 | 0.596153 | 21.06841 |
| Plant 2 TR–J | 3 | 1114.8 | 187.22 | 0.399468 | 31.44181 |
| Plant 2 TR–J | 4 | 354.2 | 90.42 | 0.544138 | 23.08237 |
| Plant 2 TR–J | 5 | 549.8 | 119.05 | 0.487232 | 25.77829 |
| Plant 2 TR–J | 6 | 383.3 | 103.12 | 0.452734 | 27.74259 |
| Plant 2 TR–J | 7 | 361.3 | 102.43 | 0.432517 | 29.03932 |
| Plant 2 TR–J | 8 | 220.7 | 80.24 | 0.430537 | 29.17289 |
