# Supplementary material for: Increased Atmospheric SO2 Detected from Changes in Leaf Physiognomy across the Triassic–Jurassic Boundary Interval of East Greenland
Source: PLoS One. 2013 Apr 10;8(4):e60614. doi: 10.1371/journal.pone.0060614 (PMC3622679; doi:10.1371/journal.pone.0060614)
Supplement: Table S6 — All measured values for all fossil Elatocladus leaves measured in the analysis. (DOC) [file pone.0060614.s006.doc]

Table S6: All measured values for all fossil *Elatocladus* leaves measured in the analysis. Gray shading indicated that the value was an outlier (over twice the standard deviation of the mean value) and was not included in analyses. Samples are held in the Field Museum of Natural History, Chicago, Illinois, USA.

| Bed | Sample number | Height (cm) | Area (mm^2^) | Perimeter (mm) | Shape Factor | Compactness |
| --- | --- | --- | --- | --- | --- | --- |
| 1.5 | 48883 | 2301-2304 | 17.616 | 25.35 | 0.345 | 36.471 |
| 1.5 | 48883 | 2301-2304 | 10.467 | 22.30 | 0.264 | 47.515 |
| 1.5 | 48883 | 2301-2304 | 15.122 | 35.15 | 0.154 | 81.713 |
| 1.5 | 48883 | 2301-2304 | 8.17 | 31.69 | 0.102 | 122.951 |
| 1.5 | 48883 | 2301-2304 | 12.362 | 40.08 | 0.097 | 129.934 |
| 1.5 | 46850 | 2304 | 8.018 | 15.15 | 0.439 | 28.626 |
| 1.5 | 46850 | 2304 | 6.701 | 14.47 | 0.402 | 31.259 |
| 1.5 | 46850 | 2304 | 8.616 | 19.47 | 0.286 | 44.006 |
| 1.5 | 46850 | 2304 | 7.079 | 20.11 | 0.220 | 57.151 |
| 1.5 | 46850 | 2304 | 8.628 | 18.41 | 0.320 | 39.270 |
| 1.5 | 46850 | 2304 | 7.617 | 20.76 | 0.222 | 56.554 |
| 2 | 47069 | 3361 | 8.686 | 21.25 | 0.242 | 51.973 |
| 2 | 47069 | 3361 | 5.524 | 18.16 | 0.211 | 59.694 |
| 2 | 47069 | 3361 | 5.204 | 20.24 | 0.160 | 78.696 |
| 2 | 47106 | 3361 | 4.506 | 15.08 | 0.249 | 50.454 |
| 2 | 47106 | 3361 | 3.786 | 13.76 | 0.251 | 50.039 |
| 2 | 47106 | 3361 | 3.465 | 14.60 | 0.204 | 61.501 |
| 2 | 47106 | 3361 | 4.212 | 14.88 | 0.239 | 52.546 |
| 2 | 47106 | 3361 | 5.979 | 16.67 | 0.271 | 46.450 |
| 2 | 47071 | 3363 | 10.04 | 24.90 | 0.203 | 61.769 |
| 2 | 47071 | 3363 | 6.565 | 15.97 | 0.323 | 38.858 |
| 2 | 47071 | 3363 | 6.724 | 20.81 | 0.195 | 64.392 |
| 2 | 47055 | 3361 | 3.07 | 15.98 | 0.151 | 83.169 |
| 2 | 47055 | 3361 | 5.021 | 17.96 | 0.196 | 64.207 |
| 2 | 47055 | 3361 | 4.322 | 17.69 | 0.174 | 72.422 |
| 2 | 47056 | 3365 | 16.678 | 21.56 | 0.451 | 27.866 |
| 2 | 47056 | 3365 | 14.578 | 22.33 | 0.367 | 34.201 |
| 2 | 47056 | 3365 | 5.835 | 16.96 | 0.255 | 49.273 |
| 2 | 47056 | 3365 | 5.061 | 17.38 | 0.211 | 59.650 |
| 2 | 47056 | 3365 | 5.111 | 14.73 | 0.296 | 42.464 |
| 2 | 47056 | 3365 | 9.461 | 14.63 | 0.555 | 22.635 |
| 2 | 47056 | 3365 | 7.025 | 14.80 | 0.403 | 31.176 |
| 2 | 47056 | 3365 | 8.494 | 15.30 | 0.456 | 27.541 |
| 2 | 47056 | 3365 | 5.078 | 16.13 | 0.245 | 51.211 |
| 2 | 47056 | 3365 | 7.15 | 21.48 | 0.195 | 64.500 |
| 2 | 48862 | 3403 | 6.267 | 15.26 | 0.338 | 37.177 |
| 2 | 48862 | 3403 | 4.064 | 16.04 | 0.199 | 63.284 |
| 2 | 48862 | 3403 | 3.031 | 9.55 | 0.417 | 30.103 |
| 2 | 48862 | 3403 | 4.39 | 12.10 | 0.377 | 33.334 |
| 2 | 48862 | 3403 | 6.084 | 13.66 | 0.410 | 30.661 |
| 2 | 47026 | 3403 | 6.938 | 19.92 | 0.220 | 57.193 |
| 2 | 47026 | 3403 | 6.026 | 14.72 | 0.349 | 35.957 |
| 2 | 47026 | 3403 | 5.574 | 12.91 | 0.421 | 29.878 |
| 2 | 47026 | 3403 | 8.114 | 18.55 | 0.296 | 42.408 |
| 2 | 47026 | 3403 | 7.388 | 19.30 | 0.249 | 50.439 |
| 2 | 46999 | 3403 | 9.188 | 22.11 | 0.236 | 53.201 |
| 2 | 46999 | 3403 | 9.993 | 25.88 | 0.187 | 67.024 |
| 2 | 46999 | 3403 | 13.582 | 26.93 | 0.235 | 53.404 |
| 2 | 46999 | 3403 | 10.298 | 27.68 | 0.169 | 74.406 |
| 2 | 46999 | 3403 | 12.132 | 27.93 | 0.195 | 64.304 |
| 4 | 47279 | 4070-4080 | 4.093 | 11.82 | 0.368 | 34.129 |
| 4 | 47279 | 4070-4080 | 4.826 | 11.19 | 0.485 | 25.932 |
| 4 | 47279 | 4070-4080 | 1.663 | 6.86 | 0.444 | 28.273 |
| 4 | 47279 | 4070-4080 | 2.7 | 10.81 | 0.290 | 43.288 |
| 4 | 47279 | 4070-4080 | 2.537 | 9.02 | 0.392 | 32.048 |
| 5 | 47986 | 4657 | 32.739 | 38.23 | 0.282 | 44.633 |
| 5 | 47986 | 4657 | 24.16 | 33.35 | 0.273 | 46.044 |
| 5 | 47986 | 4657 | 19.668 | 20.95 | 0.563 | 22.316 |
| 5 | 47986 | 4657 | 31.649 | 30.46 | 0.429 | 29.310 |
| 5 | 47986 | 4657 | 48.873 | 50.89 | 0.237 | 52.984 |
