# Supplementary material for: Increased Atmospheric SO2 Detected from Changes in Leaf Physiognomy across the Triassic–Jurassic Boundary Interval of East Greenland
Source: PLoS One. 2013 Apr 10;8(4):e60614. doi: 10.1371/journal.pone.0060614 (PMC3622679; doi:10.1371/journal.pone.0060614)
Supplement: Table S7 — All measured values for all fossil Podozamites leaves measured in the analysis. (DOC) [file pone.0060614.s007.doc]

Table S7: All measured values for all fossil *Podozamites* leaves measured in the analysis. Gray shading indicated that the value was an outlier (over twice the standard deviation of the mean value) and was not included in analyses. Samples are held in the Field Museum of Natural History, Chicago, Illinois, USA.

| Bed | Sample number | Height (cm) | Area (mm^2^) | Perimeter (mm) | Shape Factor | Compactness |
| --- | --- | --- | --- | --- | --- | --- |
| 1 | 46862 | 1378 | 432.8 | 271.80 | 0.074 | 170.691 |
| 1 | 46869 | 1378 | 147.2 | 106.30 | 0.164 | 76.764 |
| 1 | 46886 | 1398 | 66.2 | 82.95 | 0.121 | 103.938 |
| 1 | 46910 | 1363 | 308.3 | 183.30 | 0.115 | 108.981 |
| 1 | 46912 | 1363 | 757.9 | 255.49 | 0.146 | 86.126 |
| 1 | 46912 | 1363 | 462.6 | 206.15 | 0.137 | 91.867 |
| 1 | 46919 | 1398 | 103.9 | 96.90 | 0.139 | 90.372 |
| 1 | 46919 | 1398 | 50.4 | 79.23 | 0.101 | 124.551 |
| 1 | 46919 | 1398 | 40.2 | 74.78 | 0.090 | 139.106 |
| 1 | 46941 | 1378 | 42.4 | 64.29 | 0.129 | 97.481 |
| 1 | 46941 | 1378 | 22.5 | 63.66 | 0.070 | 180.115 |
| 1 | 46941 | 1378 | 30.1 | 45.88 | 0.180 | 69.933 |
| 1 | 46960 | 1363 | 95.7 | 93.00 | 0.139 | 90.376 |
| 1 | 46960 | 1363 | 42.2 | 51.63 | 0.199 | 63.167 |
| 1 | 46960 | 1363 | 26.9 | 40.63 | 0.204 | 61.368 |
| 1 | 46960 | 1364 | 24.2 | 52.95 | 0.108 | 115.855 |
| 1 | 46961 | 1363 | 51.9 | 68.48 | 0.139 | 90.357 |
| 1 | 46961 | 1363 | 43 | 59.98 | 0.150 | 83.665 |
| 2 | 46985 | 3348.5 | 34 | 46.55 | 0.197 | 63.732 |
| 2 | 46985 | 3348.5 | 11.5 | 28.92 | 0.172 | 72.728 |
| 2 | 46985 | 3348.5 | 30.5 | 36.05 | 0.295 | 42.610 |
| 2 | 46985 | 3348.5 | 64.5 | 75.54 | 0.142 | 88.470 |
| 2 | 46989 | 3348.5 | 158.7 | 98.85 | 0.204 | 61.571 |
| 2 | 46990 | 3348.5 | 84.1 | 92.00 | 0.125 | 100.642 |
| 2 | 46990 | 3348.5 | 75.3 | 86.79 | 0.126 | 100.033 |
| 2 | 46990 | 3348.5 | 49.6 | 63.21 | 0.156 | 80.555 |
| 2 | 46997 | 3361 | 256 | 113.38 | 0.250 | 50.215 |
| 2 | 47026 | 3363 | 176.1 | 133.53 | 0.124 | 101.251 |
| 2 | 47026 | 3363 | 190.8 | 98.21 | 0.249 | 50.551 |
| 2 | 47027 | 3363 | 149.2 | 108.74 | 0.159 | 79.252 |
| 2 | 47044 | 3361 | 168.5 | 139.29 | 0.109 | 115.144 |
| 2 | 47044 | 3361 | 86.3 | 85.47 | 0.148 | 84.648 |
| 2 | 47044 | 3361 | 104.2 | 82.51 | 0.192 | 65.335 |
| 2 | 47044 | 3361 | 55.9 | 67.96 | 0.152 | 82.622 |
| 2 | 47052 | 3363 | 86.6 | 64.76 | 0.260 | 48.428 |
| 2 | 47053 | 3363 | 142.9 | 85.44 | 0.246 | 51.085 |
| 2 | 47106 | 3363 | 91 | 73.15 | 0.214 | 58.801 |
| 2 | 47106 | 3363 | 57.6 | 56.77 | 0.225 | 55.952 |
| 2 | 47106 | 3363 | 41.9 | 60.36 | 0.144 | 86.953 |
| 2 | 48873 | 3361 | 90.8 | 100.69 | 0.113 | 111.657 |
| 2 | 48873 | 3361 | 115 | 108.61 | 0.123 | 102.575 |
| 3 | 47171 | 3758 | 102.5 | 62.32 | 0.332 | 37.891 |
| 3 | 47173 | 3758 | 171 | 102.32 | 0.205 | 61.224 |
| 3 | 47173 | 3758 | 206.1 | 94.55 | 0.290 | 43.376 |
| 3 | 47174 | 3758 | 107.2 | 64.80 | 0.321 | 39.170 |
| 3 | 47174 | 3758 | 141.1 | 122.46 | 0.118 | 106.282 |
| 3 | 47176 | 3758 | 199.5 | 93.58 | 0.286 | 43.896 |
| 3 | 47176 | 3758 | 169 | 114.35 | 0.162 | 77.372 |
| 3 | 47178 | 3765 | 221.6 | 95.09 | 0.308 | 40.804 |
| 3 | 47178 | 3765 | 155.4 | 101.72 | 0.189 | 66.583 |
| 3 | 47179 | 3765 | 63.1 | 64.10 | 0.193 | 65.116 |
| 3 | 47182 | 3750 | 71.8 | 58.86 | 0.261 | 48.252 |
| 3 | 47182 | 3750 | 81.1 | 63.43 | 0.253 | 49.610 |
| 3 | 47182 | 3750 | 45.6 | 41.80 | 0.328 | 38.317 |
| 3 | 47182 | 3750 | 81.1 | 63.43 | 0.253 | 49.610 |
| 3 | 47182 | 3750 | 45.6 | 41.80 | 0.328 | 38.317 |
| 3 | 47184 | 3750 | 172.5 | 90.51 | 0.265 | 47.490 |
| 3 | 47184 | 3750 | 67.5 | 56.49 | 0.266 | 47.276 |
| 3 | 47185 | 3775 | 199.4 | 76.31 | 0.430 | 29.204 |
| 3 | 47185 | 3775 | 66.1 | 42.78 | 0.454 | 27.687 |
| 3 | 47186 | 3775 | 66.4 | 46.31 | 0.389 | 32.298 |
| 3 | 47186 | 3775 | 103.8 | 53.20 | 0.461 | 27.266 |
| 3 | 47187 | 3758 | 610 | 171.73 | 0.260 | 48.346 |
| 3 | 47187 | 3758 | 155.6 | 107.37 | 0.170 | 74.089 |
| 3 | 47188 | 3758 | 155.1 | 117.88 | 0.140 | 89.592 |
| 3 | 47189 | 3758 | 115.4 | 75.48 | 0.255 | 49.369 |
| 3 | 47189 | 3758 | 155.4 | 101.80 | 0.188 | 66.688 |
| 3 | 47190 | 3758 | 93.2 | 73.40 | 0.217 | 57.806 |
| 3 | 47191 | 3775 | 99.6 | 83.55 | 0.179 | 70.086 |
| 3 | 47191 | 3775 | 177.1 | 119.03 | 0.157 | 80.001 |
| 3 | 47191 | 3775 | 197.2 | 125.02 | 0.159 | 79.260 |
| 3 | 47192 | 3705 | 134 | 88.37 | 0.216 | 58.278 |
| 3 | 47192 | 3705 | 85.6 | 59.69 | 0.302 | 41.623 |
| 3 | 47194 | 3777 | 247.2 | 107.77 | 0.267 | 46.984 |
| 3 | 47194 | 3777 | 200.7 | 95.70 | 0.275 | 45.633 |
| 3 | 47194 | 3777 | 172.7 | 74.44 | 0.392 | 32.086 |
| 3 | 47194 | 3777 | 181.6 | 88.83 | 0.289 | 43.451 |
| 3 | 47197 | 3765 | 165.1 | 96.86 | 0.221 | 56.825 |
| 3 | 47199 | 3758 | 104.9 | 91.45 | 0.158 | 79.725 |
| 3 | 47199 | 3758 | 89.7 | 72.67 | 0.213 | 58.873 |
| 3 | 47201 | 3758 | 92.1 | 78.78 | 0.186 | 67.386 |
| 3 | 47201 | 3758 | 122.9 | 75.19 | 0.273 | 46.001 |
| 3 | 47201 | 3758 | 82.4 | 50.32 | 0.409 | 30.729 |
| 3 | 47201 | 3758 | 58.4 | 46.41 | 0.340 | 36.882 |
| 3 | 47202 | 3758 | 40.2 | 60.74 | 0.137 | 91.775 |
| 3 | 47202 | 3758 | 65.5 | 71.01 | 0.163 | 76.984 |
| 3 | 47204 | 3758 | 134 | 79.90 | 0.264 | 47.642 |
| 3 | 47205 | 3758 | 278.9 | 109.16 | 0.294 | 42.725 |
| 3 | 47205 | 3758 | 198.2 | 94.89 | 0.277 | 45.429 |
| 3 | 47205 | 3758 | 172.4 | 83.04 | 0.314 | 39.998 |
| 3 | 47205 | 3758 | 87.2 | 65.36 | 0.256 | 48.990 |
| 3 | 47207 | 3761 | 138.6 | 83.43 | 0.250 | 50.221 |
| 3 | 47207 | 3761 | 203.3 | 115.15 | 0.193 | 65.221 |
| 3 | 47207 | 3761 | 179.2 | 117.19 | 0.164 | 76.638 |
| 3 | 47207 | 3761 | 177.8 | 95.95 | 0.243 | 51.780 |
| 3 | 47208 | 3758 | 302.6 | 120.93 | 0.260 | 48.328 |
| 3 | 47209 | 3761 | 155.8 | 122.67 | 0.130 | 96.585 |
| 3 | 47209 | 3761 | 168.7 | 140.36 | 0.108 | 116.781 |
| 3 | 47209 | 3761 | 70.3 | 45.26 | 0.432 | 29.139 |
| 3 | 47209 | 3761 | 177.9 | 140.86 | 0.113 | 111.532 |
| 3 | 47213 | unknown | 156.7 | 127.37 | 0.121 | 103.530 |
| 3 | 47214 | 3765 | 120.6 | 78.76 | 0.244 | 51.436 |
| 3 | 47217 | 3763 | 177.6 | 100.62 | 0.220 | 57.007 |
| 3 | 47217 | 3763 | 60.1 | 62.74 | 0.192 | 65.496 |
| 3 | 47217 | 3763 | 102.1 | 80.06 | 0.200 | 62.778 |
| 3 | 47217 | 3763 | 79 | 56.02 | 0.316 | 39.725 |
| 3 | 47223 | 3763 | 91.7 | 67.08 | 0.256 | 49.070 |
| 3 | 47223 | 3763 | 63.1 | 49.90 | 0.319 | 39.461 |
| 3 | 47225 | 3725 | 250.3 | 149.36 | 0.141 | 89.127 |
| 3 | 47225 | 3725 | 136.3 | 81.82 | 0.256 | 49.116 |
| 3 | 47225 | 3725 | 288.4 | 117.30 | 0.263 | 47.709 |
| 3 | 47225 | 3725 | 65.3 | 48.37 | 0.351 | 35.829 |
| 3 | 47225 | 3725 | 218.5 | 134.84 | 0.151 | 83.212 |
| 3 | 47225 | 3725 | 155.5 | 106.17 | 0.173 | 72.489 |
| 3 | 47225 | 3725 | 144.9 | 97.86 | 0.190 | 66.091 |
| 3 | 47225 | 3725 | 192.9 | 109.69 | 0.201 | 62.374 |
| 3 | 47225 | 3725 | 70.3 | 51.02 | 0.339 | 37.028 |
| 3 | 47227 | 3725 | 74.9 | 57.14 | 0.288 | 43.591 |
| 3 | 47227 | 3725 | 86.1 | 68.07 | 0.234 | 53.816 |
| 3 | 47228 | 3765 | 141.5 | 68.58 | 0.378 | 33.238 |
| 3 | 47228 | 3765 | 94.1 | 64.86 | 0.281 | 44.706 |
| 3 | 47228 | 3765 | 127.9 | 70.50 | 0.323 | 38.860 |
| 3 | 47229 | 3765 | 221.4 | 110.12 | 0.229 | 54.772 |
| 3 | 47229 | 3765 | 191 | 101.41 | 0.233 | 53.843 |
| 3 | 47229 | 3765 | 151.9 | 83.54 | 0.273 | 45.944 |
| 3 | 47230 | 3765 | 101.9 | 65.88 | 0.295 | 42.592 |
| 3 | 47230 | 3765 | 80.8 | 63.24 | 0.254 | 49.496 |
| 3 | 47230 | 3765 | 83.1 | 58.07 | 0.310 | 40.579 |
| 3 | 47231 | 3763 | 338.7 | 129.57 | 0.254 | 49.567 |
| 3 | 47231 | 3763 | 93.6 | 66.35 | 0.267 | 47.033 |
| 3 | 47231 | 3763 | 201.6 | 106.19 | 0.225 | 55.934 |
| 3 | 47233 | 3770 | 163.6 | 89.96 | 0.254 | 49.467 |
| 3 | 47233 | 3770 | 209.4 | 89.03 | 0.332 | 37.853 |
| 3 | 47234 | 3758 | 166.4 | 107.66 | 0.180 | 69.656 |
| 3 | 47234 | 3758 | 99.1 | 82.94 | 0.181 | 69.415 |
| 3 | 47234 | 3758 | 123.5 | 103.46 | 0.145 | 86.672 |
| 3 | 47235 | 3758 | 80.7 | 63.34 | 0.253 | 49.714 |
| 3 | 47235 | 3758 | 49.4 | 55.54 | 0.201 | 62.443 |
| 3 | 47235 | 3758 | 67.5 | 56.94 | 0.262 | 48.032 |
| 3 | 47235 | 3758 | 53.5 | 49.43 | 0.275 | 45.670 |
| 3 | 47236 | 3775 | 65.1 | 72.06 | 0.158 | 79.764 |
| 3 | 47239 | 3758 | 172.3 | 99.96 | 0.217 | 57.992 |
| 3 | 47239 | 3758 | 54.5 | 71.51 | 0.134 | 93.829 |
| 3 | 47240 | 3758 | 227.7 | 130.49 | 0.168 | 74.781 |
| 3 | 47240 | 3758 | 61.4 | 64.87 | 0.183 | 68.536 |
| 3 | 47242 | 3775 | 105.5 | 76.26 | 0.228 | 55.124 |
| 3 | 47245 | 3758 | 179.4 | 98.44 | 0.233 | 54.016 |
| 3 | 47247 | 3775 | 95.3 | 74.08 | 0.218 | 57.585 |
| 3 | 47247 | 3775 | 151.1 | 83.89 | 0.270 | 46.575 |
| 3 | 47247 | 3775 | 104.7 | 60.51 | 0.359 | 34.971 |
| 3 | 47249 | 3758 | 259.2 | 110.36 | 0.267 | 46.988 |
| 3 | 47249 | 3758 | 1010.6 | 180.81 | 0.388 | 32.349 |
| 3 | 47249 | 3758 | 183.2 | 100.27 | 0.229 | 54.880 |
| 3 | 47249 | 3758 | 137.8 | 72.75 | 0.327 | 38.408 |
| 3 | 47249 | 3758 | 197.4 | 104.06 | 0.229 | 54.856 |
| 3 | 47251 | 3758 | 180.7 | 89.05 | 0.286 | 43.884 |
| 3 | 47251 | 3758 | 93 | 71.44 | 0.229 | 54.878 |
| 3 | 47251 | 3758 | 125.7 | 90.47 | 0.193 | 65.114 |
| 3 | 47253 | 3758 | 153.3 | 103.74 | 0.179 | 70.202 |
| 3 | 47253 | 3758 | 70.3 | 76.09 | 0.153 | 82.357 |
| 3 | 47254 | 3758 | 117.6 | 92.44 | 0.173 | 72.663 |
| 3 | 47258 | 3758 | 271.2 | 122.56 | 0.227 | 55.387 |
| 3 | 47258 | 3758 | 311.8 | 110.96 | 0.318 | 39.487 |
| 3 | 47258 | 3758 | 166.7 | 116.60 | 0.154 | 81.557 |
| 3 | 47258 | 3758 | 233 | 142.16 | 0.145 | 86.736 |
| 3 | 47260 | 3758 | 54 | 59.55 | 0.191 | 65.670 |
| 3 | 47260 | 3758 | 47.8 | 63.89 | 0.147 | 85.396 |
| 3 | 47261 | 3758 | 299.6 | 139.80 | 0.193 | 65.234 |
| 3 | 47261 | 3758 | 219 | 127.83 | 0.168 | 74.614 |
| 3 | 47265 | 3748 | 235 | 130.65 | 0.173 | 72.636 |
| 3 | 47265 | 3748 | 237 | 129.42 | 0.178 | 70.673 |
| 3 | 47265 | 3748 | 191.5 | 95.46 | 0.264 | 47.585 |
| 3 | 47265 | 3748 | 144.4 | 83.19 | 0.262 | 47.926 |
| 3 | 47267 | 3761 | 72 | 66.43 | 0.205 | 61.291 |
| 3 | 47267 | 3761 | 84.8 | 75.00 | 0.189 | 66.333 |
| 3 | 47267 | 3761 | 79.2 | 72.11 | 0.191 | 65.655 |
| 3 | 47269 | 3725 | 85.8 | 59.59 | 0.304 | 41.387 |
| 3 | 47269 | 3725 | 97.5 | 60.37 | 0.336 | 37.380 |
| 3 | 47269 | 3725 | 93.5 | 57.80 | 0.352 | 35.731 |
| 3 | 47270 | 3725 | 104.4 | 79.14 | 0.210 | 59.992 |
| 3 | 47270 | 3725 | 40.8 | 49.76 | 0.207 | 60.688 |
| 3 | 47270 | 3725 | 118.8 | 76.37 | 0.256 | 49.094 |
| 3 | 47271 | 3725 | 118.8 | 73.84 | 0.274 | 45.895 |
| 3 | 47271 | 3725 | 114.5 | 80.35 | 0.223 | 56.385 |
| 3 | 47272 | 3725 | 91.4 | 50.65 | 0.448 | 28.068 |
| 3 | 47272 | 3725 | 216.7 | 90.91 | 0.329 | 38.139 |
| 3 | 47274 | 3758 | 106.8 | 69.17 | 0.280 | 44.799 |
| 3 | 47275 | 3758 | 481.5 | 147.11 | 0.280 | 44.946 |
| 3 | 47275 | 3758 | 635.3 | 151.07 | 0.350 | 35.923 |
| 3 | 47275 | 3758 | 239.5 | 100.53 | 0.298 | 42.197 |
| 3 | 48127 | 3765 | 117.3 | 76.39 | 0.253 | 49.748 |
| 3 | 48127 | 3765 | 78.2 | 61.05 | 0.264 | 47.661 |
| 3 | 48130 | 3758 | 46.7 | 50.66 | 0.229 | 54.956 |
| 3 | 48130 | 3758 | 81.8 | 72.68 | 0.194 | 64.577 |
| 3 | 48130 | 3758 | 63 | 61.10 | 0.212 | 59.257 |
| 3 | 48131 | 3758 | 140.9 | 82.15 | 0.262 | 47.897 |
| 3 | 48131 | 3758 | 119.7 | 96.73 | 0.161 | 78.168 |
| 3 | 48131 | 3758 | 100 | 67.93 | 0.272 | 46.145 |
| 3 | 48131 | 3758 | 112.3 | 86.85 | 0.187 | 67.168 |
| 3 | 48131 | 3758 | 122 | 75.14 | 0.272 | 46.279 |
| 3 | 48132 | 3758 | 146.1 | 79.85 | 0.288 | 43.641 |
| 3 | 48132 | 3758 | 55.4 | 55.08 | 0.230 | 54.762 |
| 3 | 48133 | 3758 | 67.8 | 45.98 | 0.403 | 31.182 |
| 3 | 48133 | 3758 | 45.7 | 49.94 | 0.230 | 54.573 |
| 3 | 48134 | 3758 | 43.8 | 48.53 | 0.234 | 53.771 |
| 3 | 48134 | 3758 | 116.4 | 64.22 | 0.355 | 35.431 |
| 3 | 48135 | 3758 | 172.6 | 127.53 | 0.133 | 94.229 |
| 3 | 48135 | 3758 | 88.2 | 79.97 | 0.173 | 72.508 |
| 3 | 48135 | 3758 | 120.1 | 88.13 | 0.194 | 64.670 |
| 3 | 48135 | 3758 | 98.6 | 95.19 | 0.137 | 91.898 |
| 3 | 50002 | 3765 | 83.2 | 70.90 | 0.208 | 60.418 |
| 3 | 50002 | 3765 | 187 | 83.02 | 0.341 | 36.857 |
| 3 | 50002 | 3765 | 107.9 | 64.77 | 0.323 | 38.880 |
| 3 | 50003 | 3758 | 366.5 | 101.89 | 0.444 | 28.326 |
| 3 | 50004 | 3761 | 198.2 | 102.63 | 0.237 | 53.143 |
| 3 | 50004 | 3761 | 55 | 55.44 | 0.225 | 55.884 |
| 3 | 50004 | 3761 | 235.8 | 107.55 | 0.256 | 49.054 |
| 3 | 50005 | 3761 | 277.9 | 96.82 | 0.373 | 33.732 |
| 3 | 50006 | 3761 | 64.9 | 65.16 | 0.192 | 65.421 |
| 4 | 47138 | 4070 | 992.5 | 230.08 | 0.236 | 53.337 |
| 4 | 47318 | 4080-4095 | 125.2 | 88.10 | 0.203 | 61.994 |
| 4 | 47318 | 4080-4095 | 152.5 | 104.70 | 0.175 | 71.883 |
| 4 | 47318 | 4080-4095 | 147.8 | 114.11 | 0.143 | 88.099 |
| 4 | 47318 | 4080-4095 | 53 | 66.10 | 0.152 | 82.438 |
| 4 | 47439 | 4061 | 60.6 | 56.76 | 0.236 | 53.163 |
| 4 | 47439 | 4061 | 29.4 | 54.48 | 0.125 | 100.955 |
| 4 | 47439 | 4061 | 31.6 | 52.74 | 0.143 | 88.022 |
| 5 | 47018 | 4658 | 387 | 126.19 | 0.305 | 41.147 |
| 5 | 47035 | 4650 | 399.5 | 141.28 | 0.252 | 49.963 |
| 5 | 47035 | 4650 | 343.7 | 97.81 | 0.451 | 27.835 |
| 5 | 47519 | 4658 | 228.2 | 84.58 | 0.401 | 31.349 |
| 5 | 47519 | 4658 | 79.1 | 56.41 | 0.312 | 40.229 |
| 5 | 47519 | 4658 | 105 | 50.92 | 0.509 | 24.694 |
| 5 | 47519 | 4658 | 81.8 | 43.51 | 0.543 | 23.143 |
| 5 | 47522 | 4658 | 173.4 | 68.27 | 0.468 | 26.879 |
| 5 | 47522 | 4658 | 135.8 | 56.92 | 0.527 | 23.858 |
| 5 | 47522 | 4658 | 128.7 | 56.83 | 0.501 | 25.094 |
| 5 | 47522 | 4658 | 251.8 | 84.58 | 0.442 | 28.411 |
| 5 | 47534 | 4653 | 291.7 | 93.71 | 0.417 | 30.105 |
| 5 | 47534 | 4653 | 108.5 | 65.35 | 0.319 | 39.361 |
| 5 | 47534 | 4653 | 214.3 | 91.06 | 0.325 | 38.693 |
| 5 | 47535 | 4653 | 312.9 | 105.07 | 0.356 | 35.282 |
| 5 | 47535 | 4653 | 370.9 | 137.05 | 0.248 | 50.641 |
| 5 | 47535 | 4653 | 278.7 | 101.98 | 0.337 | 37.316 |
| 5 | 47536 | 4653 | 307.2 | 98.58 | 0.397 | 31.634 |
| 5 | 47540 | 4654 | 273.6 | 96.12 | 0.372 | 33.768 |
| 5 | 47593 | 4643 | 108.6 | 64.17 | 0.331 | 37.917 |
| 5 | 47593 | 4643 | 126.6 | 78.21 | 0.260 | 48.316 |
| 5 | 47596 | 4663 | 110 | 60.84 | 0.373 | 33.650 |
| 5 | 47596 | 4663 | 82.8 | 62.63 | 0.265 | 47.373 |
| 5 | 47610 | 4646 | 271.1 | 101.31 | 0.332 | 37.860 |
| 5 | 47610 | 4646 | 320.5 | 102.00 | 0.387 | 32.462 |
| 5 | 47631 | 4647 | 272 | 115.23 | 0.257 | 48.816 |
| 5 | 47639 | 4661 | 132.3 | 73.11 | 0.311 | 40.401 |
| 5 | 47639 | 4661 | 236.9 | 83.63 | 0.426 | 29.523 |
| 5 | 47639 | 4661 | 112.6 | 65.02 | 0.335 | 37.545 |
| 5 | 47656 | 4664 | 469.8 | 105.69 | 0.528 | 23.777 |
| 5 | 47656 | 4664 | 177.5 | 108.76 | 0.189 | 66.641 |
| 5 | 47656 | 4664 | 205.4 | 96.63 | 0.276 | 45.459 |
| 5 | 47663 | 4678 | 92.5 | 40.91 | 0.694 | 18.093 |
| 5 | 47892 | 4653 | 279.4 | 108.07 | 0.301 | 41.801 |
| 5 | 47895 | 4648 | 67 | 43.00 | 0.456 | 27.597 |
| 5 | 47895 | 4648 | 35.3 | 42.00 | 0.252 | 49.972 |
| 5 | 47896 | 4653 | 185.3 | 70.09 | 0.474 | 26.512 |
| 5 | 47896 | 4653 | 222.2 | 77.71 | 0.462 | 27.178 |
| 5 | 47896 | 4653 | 114.4 | 55.33 | 0.470 | 26.761 |
| 5 | 47896 | 4653 | 237.7 | 89.66 | 0.372 | 33.820 |
| 5 | 47907 | 4656 | 119 | 71.56 | 0.292 | 43.032 |
| 5 | 47907 | 4656 | 404.3 | 99.67 | 0.511 | 24.571 |
| 5 | 47937 | 4664 | 100.1 | 76.91 | 0.213 | 59.092 |
| 5 | 47953 | 4658 | 151.7 | 68.81 | 0.403 | 31.212 |
| 5 | 47953 | 4658 | 38.4 | 32.61 | 0.454 | 27.693 |
| 5 | 47953 | 4658 | 96.6 | 58.26 | 0.358 | 35.137 |
| 5 | 47953 | 4658 | 127.2 | 60.26 | 0.440 | 28.548 |
| 5 | 47953 | 4658 | 113 | 53.83 | 0.490 | 25.643 |
| 5 | 47954 | 4658 | 214.9 | 105.94 | 0.241 | 52.226 |
| 5 | 47954 | 4658 | 121.2 | 71.04 | 0.302 | 41.639 |
| 5 | 47962 | 4644 | 242.8 | 94.78 | 0.340 | 36.999 |
| 5 | 47962 | 4644 | 410.5 | 129.04 | 0.310 | 40.564 |
| 5 | 48027 | 4665 | 217.9 | 100.06 | 0.274 | 45.948 |
| 5 | 48027 | 4665 | 166 | 106.93 | 0.182 | 68.880 |
| 5 | 48027 | 4665 | 386.6 | 102.66 | 0.461 | 27.261 |
| 5 | 48027 | 4665 | 341.7 | 99.90 | 0.430 | 29.207 |
| 5 | 48027 | 4665 | 221.4 | 87.70 | 0.362 | 34.739 |
| 5 | 48028 | 4663 | 300.5 | 108.65 | 0.320 | 39.284 |
| 5 | 48030 | 4673 | 137.8 | 83.02 | 0.251 | 50.017 |
| 5 | 48030 | 4673 | 90.8 | 66.13 | 0.261 | 48.163 |
| 5 | 48030 | 4673 | 116.5 | 72.43 | 0.279 | 45.031 |
| 5 | 48030 | 4673 | 215.9 | 100.61 | 0.268 | 46.885 |
| 5 | 48033 | 4638 | 396.3 | 126.79 | 0.310 | 40.564 |
| 5 | 48033 | 4638 | 281.6 | 102.52 | 0.337 | 37.324 |
| 5 | 48033 | 4638 | 262.4 | 114.96 | 0.250 | 50.365 |
| 5 | 48046 | 4648 | 187 | 79.73 | 0.370 | 33.994 |
| 5 | 48046 | 4648 | 136.1 | 71.16 | 0.338 | 37.206 |
| 5 | 48046 | 4648 | 187.9 | 87.39 | 0.309 | 40.644 |
| 5 | 48047 | 4643 | 272.8 | 115.74 | 0.256 | 49.105 |
| 5 | 48047 | 4643 | 289.6 | 107.92 | 0.312 | 40.217 |
| 5 | 48048 | 4643 | 220.3 | 86.15 | 0.373 | 33.690 |
| 5 | 48048 | 4643 | 302.3 | 90.92 | 0.460 | 27.345 |
| 5 | 48073 | 4666 | 360.8 | 121.29 | 0.308 | 40.774 |
| 5 | 48073 | 4666 | 370.6 | 127.14 | 0.288 | 43.617 |
| 5 | 48073 | 4666 | 372 | 123.16 | 0.308 | 40.775 |
| 5 | 48073 | 4666 | 702.5 | 176.76 | 0.283 | 44.476 |
| 5 | 48073 | 4666 | 610.3 | 143.86 | 0.371 | 33.911 |
| 5 | 48075 | 4668 | 224.1 | 96.22 | 0.304 | 41.313 |
| 5 | 48084 | 4638 | 451.4 | 127.12 | 0.351 | 35.799 |
| 5 | 48084 | 4638 | 168.8 | 70.84 | 0.423 | 29.729 |
| 5 | 48084 | 4638 | 406.8 | 125.30 | 0.326 | 38.594 |
| 5 | 48084 | 4638 | 282.2 | 129.00 | 0.213 | 58.969 |
| 5 | 48358 | 4643 | 211.6 | 86.15 | 0.358 | 35.075 |
| 5 | 48358 | 4643 | 172.5 | 81.56 | 0.326 | 38.563 |
| 5 | 48360 | 4643 | 82.1 | 48.25 | 0.443 | 28.356 |
| 5 | 48361 | 4643 | 189.4 | 86.05 | 0.321 | 39.095 |
| 5 | 48367 | 4668 | 463.7 | 126.00 | 0.367 | 34.238 |
| 5 | 48367 | 4668 | 85.7 | 55.91 | 0.345 | 36.475 |
| 5 | 48368 | 4668 | 838.7 | 137.63 | 0.556 | 22.585 |
| 5 | 48368 | 4668 | 466 | 114.39 | 0.448 | 28.080 |
| 5 | 48368 | 4668 | 389.9 | 104.07 | 0.452 | 27.778 |
| 5 | 48368 | 4668 | 143.9 | 57.31 | 0.550 | 22.824 |
| 5 | 48369 | 4668 | 384.7 | 111.08 | 0.392 | 32.074 |
| 5 | 48369 | 4668 | 190.7 | 81.31 | 0.363 | 34.669 |
| 5 | 48375 | 4666 | 444.9 | 138.31 | 0.292 | 42.998 |
| 5 | 48375 | 4666 | 551.8 | 151.81 | 0.301 | 41.766 |
| 5 | 48375 | 4666 | 406.2 | 117.48 | 0.370 | 33.977 |
| 5 | 48375 | 4666 | 708 | 165.18 | 0.326 | 38.537 |
| 5 | 48375 | 4666 | 880.6 | 149.24 | 0.497 | 25.293 |
| 5 | 48375 | 4666 | 268.3 | 90.05 | 0.416 | 30.224 |
| 5 | 48391 | 4638 | 289.1 | 91.63 | 0.433 | 29.042 |
| 5 | 48781 | 4646 | 257.6 | 104.08 | 0.299 | 42.052 |
| 5 | 48781 | 4646 | 152.2 | 71.06 | 0.379 | 33.177 |
| 5 | 48781 | 4646 | 47.1 | 39.74 | 0.375 | 33.530 |
| 5 | 48798 | 4663 | 326.7 | 126.71 | 0.256 | 49.144 |
| 5 | 48800 | 4663 | 266.1 | 96.63 | 0.358 | 35.090 |
| 5 | 48800 | 4663 | 133.5 | 74.16 | 0.305 | 41.196 |
| 5 | 48822 | 4650 | 264.2 | 98.47 | 0.342 | 36.701 |
| 5 | 48825 | 4652 | 292.5 | 112.37 | 0.291 | 43.169 |
| 5 | 48825 | 4652 | 271.8 | 111.04 | 0.277 | 45.364 |
| 5 | 48825 | 4652 | 354.2 | 107.13 | 0.388 | 32.402 |
| 5 | 48826 | 4652 | 238.2 | 96.96 | 0.318 | 39.468 |
| 5 | 48827 | 4652 | 179 | 73.85 | 0.412 | 30.468 |
| 5 | 48827 | 4652 | 167.7 | 78.69 | 0.340 | 36.924 |
| 5 | 48828 | 4652 | 199.2 | 93.05 | 0.289 | 43.465 |
| 5 | 48829 | 4652 | 188.3 | 87.75 | 0.307 | 40.893 |
| 5 | 48839 | 4652 | 456.4 | 116.91 | 0.420 | 29.947 |
| 5 | 48839 | 4668 | 226.4 | 94.39 | 0.319 | 39.353 |
| 8 | 47803 | 9151 | 377.1 | 166.97 | 0.170 | 73.930 |
| 8 | 47803 | 9151 | 397.8 | 182.93 | 0.149 | 84.121 |
| 8 | 47803 | 9151 | 239 | 132.10 | 0.172 | 73.014 |
| 8 | 47831 | 9152 | 178.3 | 137.94 | 0.118 | 106.716 |
| 8 | 47831 | 9152 | 161.7 | 100.05 | 0.203 | 61.905 |
| 8 | 47831 | 9152 | 146.4 | 83.69 | 0.263 | 47.842 |
| 8 | 47868 | 9233.7 | 148.2 | 101.87 | 0.179 | 70.024 |
| 8 | 47868 | 9233.7 | 155.4 | 104.22 | 0.180 | 69.896 |
| 8 | 47868 | 9233.7 | 175.3 | 99.24 | 0.224 | 56.181 |
| 8 | 47868 | 9233.7 | 156.1 | 106.47 | 0.173 | 72.619 |
| 8 | 47868 | 9233.7 | 220.3 | 122.49 | 0.185 | 68.106 |
| 8 | 47882 | 9098 | 174.4 | 78.44 | 0.356 | 35.280 |
| 8 | 47882 | 9098 | 231.9 | 101.63 | 0.282 | 44.539 |
| 8 | 47882 | 9098 | 180 | 94.29 | 0.254 | 49.392 |
| 8 | 47882 | 9098 | 199.7 | 97.87 | 0.262 | 47.965 |
| 8 | 47888 | 9150 | 241.1 | 97.13 | 0.321 | 39.130 |
| 8 | 47888 | 9150 | 178.1 | 95.68 | 0.244 | 51.402 |
| 8 | 51088 | 9173 | 204.1 | 136.91 | 0.137 | 91.839 |
| 8 | 51096 | 9151 | 141.8 | 78.43 | 0.290 | 43.380 |
| 8 | 51096 | 9151 | 99 | 62.65 | 0.317 | 39.647 |
| 8 | 51096 | 9151 | 76.7 | 54.73 | 0.322 | 39.053 |
