# Supplementary material for: Increased Atmospheric SO2 Detected from Changes in Leaf Physiognomy across the Triassic–Jurassic Boundary Interval of East Greenland
Source: PLoS One. 2013 Apr 10;8(4):e60614. doi: 10.1371/journal.pone.0060614 (PMC3622679; doi:10.1371/journal.pone.0060614)
Supplement: Table S10 — All measured values for all fossil Anomozamites leaves measured in the analysis. (DOC) [file pone.0060614.s010.doc]

Table S10: All measured values for all fossil *Anomozamites* leaves measured in the analysis. Gray shading indicated that the value was an outlier (over twice the standard deviation of the mean value) and was not included in analyses. Samples are held in the Field Museum of Natural History, Chicago, Illinois, USA.

| Bed | Sample number | Height (cm) | Area (mm^2^) | Perimeter (mm) | Shape Factor | Compactness |
| --- | --- | --- | --- | --- | --- | --- |
| 1 | 46871 | 1380 | 177.6 | 52.04 | 0.824 | 15.249 |
| 1 | 46874 | 1373 | 70.2 | 32.96 | 0.812 | 15.475 |
| 1 | 46875 | 1376 | 78.2 | 40.02 | 0.614 | 20.481 |
| 1 | 46872 | 1373 | 69.3 | 33.32 | 0.784 | 16.021 |
| 1 | 46872 | 1373 | 35.9 | 23.48 | 0.818 | 15.357 |
| 1 | 46888 | 1403 | 40.1 | 24.89 | 0.814 | 15.449 |
| 1 | 46887 | 1403 | 55.5 | 28.99 | 0.830 | 15.143 |
| 1 | 46885 | 1380 | 166.3 | 60.83 | 0.565 | 22.251 |
| 1 | 46893 | unknown | 48.2 | 27.10 | 0.826 | 15.237 |
| 1 | 46903 | 1368 | 65 | 31.46 | 0.826 | 15.227 |
| 1 | 46905 | 1383 | 53.3 | 28.67 | 0.816 | 15.422 |
| 1 | 46910 | 1363 | 172.6 | 55.71 | 0.699 | 17.981 |
| 1 | 46912 | 1363 | 74.1 | 32.62 | 0.875 | 14.360 |
| 1 | 46929 | 1368 | 78.5 | 35.06 | 0.802 | 15.659 |
| 1 | 46973 | 1368 | 42.2 | 25.04 | 0.845 | 14.858 |
| 1 | 46976 | 1388 | 145.7 | 53.83 | 0.632 | 19.888 |
| 1.5 | 47125 | 2301-2304 | 35.5 | 25.12 | 0.707 | 17.775 |
| 1.5 | 47137 | 2301-2304 | 67.8 | 37.35 | 0.611 | 20.576 |
| 1.5 | 47132 | 2301-2304 | 123.3 | 45.66 | 0.743 | 16.909 |
| 1.5 | 47132 | 2301-2304 | 129.6 | 54.78 | 0.543 | 23.155 |
| 1.5 | 51069 | 1350 | 37.8 | 27.38 | 0.633 | 19.832 |
| 1.5 | 46855 | 2301-2304 | 242.8 | 73.51 | 0.565 | 22.256 |
| 1.5 | 46858 | 2301-2304 | 179.2 | 72.44 | 0.429 | 29.283 |
| 1.5 | 48886 | 2301-2304 | 167.2 | 105.23 | 0.190 | 66.228 |
| 1.5 | 46839 | 2301-2304 | 95.9 | 50.44 | 0.473 | 26.530 |
| 1.5 | 46839 | 2301-2304 | 116.4 | 50.51 | 0.573 | 21.918 |
| 1.5 | 46839 | 2301-2304 | 102.3 | 50.39 | 0.506 | 24.821 |
| 1.5 | 46840 | unknown | 33.3 | 23.13 | 0.784 | 16.066 |
| 1.5 | 46849 | 2306 | 123.9 | 51.39 | 0.590 | 21.315 |
| 2 | 47118 | no record | 60 | 32.02 | 0.735 | 17.088 |
| 2 | 47118 | no record | 56.2 | 30.41 | 0.764 | 16.455 |
| 2 | 47108 | 3363 | 57.9 | 29.49 | 0.837 | 15.020 |
| 2 | 47117 | no record | 93.1 | 39.53 | 0.748 | 16.784 |
| 2 | 47073 | 3373 | 54.4 | 28.45 | 0.844 | 14.879 |
| 2 | 47061 | 3358 | 50.7 | 26.98 | 0.876 | 14.357 |
| 2 | 47048 | 3403 | 124.6 | 52.32 | 0.572 | 21.969 |
| 2 | 47008 | 3348 | 67.7 | 32.96 | 0.784 | 16.047 |
| 2 | 47003 | 3403 | 40.6 | 27.11 | 0.694 | 18.102 |
| 2 | 47096 | 3403 | 72.5 | 33.23 | 0.825 | 15.231 |
| 3 | 48125 | 3765 | 40.9 | 30.43 | 0.555 | 22.640 |
| 4 | 47140 | 4072 | 70 | 32.24 | 0.846 | 14.849 |
| 4 | 47140 | 4072 | 36.4 | 24.05 | 0.790 | 15.890 |
| 4 | 47147 | 4075 | 200.4 | 54.06 | 0.861 | 14.583 |
| 4 | 47153 | 4072 | 40.5 | 25.64 | 0.774 | 16.232 |
| 4 | 47158 | 4074 | 49.3 | 28.30 | 0.773 | 16.245 |
| 4 | 47159 | 4074 | 26.7 | 20.67 | 0.785 | 16.002 |
| 4 | 47161 | 4072 | 176.8 | 53.31 | 0.781 | 16.074 |
| 4 | 47164 | 4070-4080 | 57.6 | 29.36 | 0.839 | 14.965 |
| 4 | 47279 | 4070-4080 | 72.6 | 32.99 | 0.838 | 14.991 |
| 4 | 47294 | 4055-4070 | 47.5 | 27.60 | 0.783 | 16.037 |
| 4 | 47295 | 4055-4070 | 38.5 | 23.63 | 0.866 | 14.503 |
| 4 | 47299 | 4070-4080 | 33.1 | 22.43 | 0.826 | 15.200 |
| 4 | 47299 | 4070-4080 | 24.1 | 18.89 | 0.848 | 14.806 |
| 4 | 47303 | unknown | 62.7 | 30.82 | 0.829 | 15.149 |
| 4 | 47305 | unknown | 65.1 | 31.02 | 0.850 | 14.781 |
| 4 | 47306 | unknown | 137.8 | 44.94 | 0.857 | 14.656 |
| 4 | 47310 | 4070-4080 | 41.7 | 25.44 | 0.809 | 15.520 |
| 4 | 47313 | unknown | 63.3 | 30.99 | 0.828 | 15.172 |
| 4 | 47332 | 4080-4095 | 0 | 0.00 |  |  |
| 4 | 47333 | 4070-4080 | 55.1 | 28.69 | 0.841 | 14.939 |
| 4 | 47346 | 4055-4070 | 84.1 | 36.01 | 0.815 | 15.419 |
| 4 | 47347 | 4055-4070 | 6.8 | 9.75 | 0.898 | 13.980 |
| 4 | 47359 | 4055-4070 | 138.1 | 45.90 | 0.823 | 15.256 |
| 4 | 47360 | 4055-4070 | 36.4 | 23.62 | 0.819 | 15.327 |
| 4 | 47361 | 4055-4070 | 95.2 | 37.50 | 0.850 | 14.772 |
| 4 | 47362 | 4055-4070 | 212.6 | 57.94 | 0.795 | 15.790 |
| 4 | 47364 | 4055-4070 | 68.8 | 31.39 | 0.877 | 14.322 |
| 4 | 47369 | unknown | 89.6 | 38.13 | 0.774 | 16.227 |
| 4 | 47370 | 4055-4070 | 43.6 | 25.57 | 0.838 | 14.996 |
| 4 | 47376 | 4055-4070 | 57.2 | 29.08 | 0.850 | 14.784 |
| 4 | 47378 | 4080 | 29.9 | 21.75 | 0.794 | 15.821 |
| 4 | 47381 | 4075 | 76.7 | 33.42 | 0.863 | 14.562 |
| 4 | 47382 | 4075 | 101.8 | 40.00 | 0.799 | 15.717 |
| 4 | 47394 | 4080 | 60.1 | 29.77 | 0.852 | 14.746 |
| 4 | 47405 | 4067 | 42.9 | 25.68 | 0.817 | 15.372 |
| 4 | 47406 | 4067 | 43.2 | 24.82 | 0.881 | 14.260 |
| 4 | 47409 | 4067 | 48.9 | 27.11 | 0.836 | 15.030 |
| 4 | 47410 | 4061 | 34.6 | 22.90 | 0.829 | 15.156 |
| 4 | 47411 | 4080 | 35.2 | 23.92 | 0.773 | 16.255 |
| 4 | 47414 | 4072 | 191.7 | 53.92 | 0.828 | 15.166 |
| 4 | 47415 | 4072 | 114.5 | 42.21 | 0.807 | 15.561 |
| 4 | 47426 | 4061 | 131 | 45.42 | 0.798 | 15.748 |
| 4 | 47427 | 4080 | 65.2 | 31.29 | 0.836 | 15.016 |
| 4 | 47427 | 4080 | 33.7 | 22.01 | 0.874 | 14.375 |
| 4 | 47430 | 4100 | 21.8 | 18.28 | 0.819 | 15.328 |
| 4 | 47435 | 4080 | 57.3 | 29.89 | 0.806 | 15.592 |
| 4 | 47439 | 4061 | 62.6 | 30.66 | 0.836 | 15.017 |
| 4 | 47439 |  | 26.4 | 20.02 | 0.827 | 15.182 |
| 4 | 47440 | 4080 | 58.2 | 30.00 | 0.812 | 15.464 |
| 4 | 47445 | 4075 | 33.9 | 22.28 | 0.858 | 14.643 |
| 4 | 47451 | 4063 | 198.2 | 54.98 | 0.824 | 15.251 |
| 4 | 47453 | 4070-4080 | 76.9 | 34.75 | 0.800 | 15.703 |
| 4 | 47455 | 4089 | 43.9 | 25.53 | 0.846 | 14.847 |
| 4 | 47457 | 4090 | 90.1 | 35.76 | 0.885 | 14.193 |
| 4 | 47458 | 4090 | 44.3 | 24.88 | 0.899 | 13.973 |
| 4 | 47459 | 4059 | 68.6 | 32.94 | 0.794 | 15.817 |
| 4 | 47463 | 4061 | 170.8 | 50.68 | 0.835 | 15.038 |
| 4 | 47463 | 4061 | 64.7 | 31.57 | 0.815 | 15.404 |
| 4 | 47463 | 4061 | 47.5 | 27.63 | 0.781 | 16.072 |
| 4 | 47471 | 4061 | 68.6 | 31.39 | 0.874 | 14.363 |
| 4 | 47471 | 4061 | 27.6 | 20.74 | 0.806 | 15.585 |
| 4 | 47481 | 4061 | 153.1 | 47.58 | 0.849 | 14.787 |
| 4 | 47486 | 4070-4080 | 77.2 | 34.02 | 0.838 | 14.992 |
| 4 | 47498 | 4055-4070 | 239.5 | 60.18 | 0.831 | 15.122 |
| 4 | 47502 | 4055-4070 | 40.1 | 24.77 | 0.821 | 15.301 |
| 4 | 47659 | 4090 | 72 | 33.57 | 0.802 | 15.652 |
| 4 | 47659 | 4090 | 80.9 | 35.28 | 0.816 | 15.385 |
| 4 | 48144 | 4060 | 66 | 32.73 | 0.774 | 16.231 |
| 4 | 48147 | 4067 | 168 | 50.02 | 0.843 | 14.893 |
| 4 | 48155 | 4061 | 43.4 | 25.49 | 0.839 | 14.971 |
| 4 | 48156 | 4061 | 57.6 | 29.18 | 0.850 | 14.783 |
| 4 | 48167 | 4055-4070 | 10.5 | 12.86 | 0.797 | 15.750 |
| 4 | 48168 | 4070-4080 | 30.3 | 21.55 | 0.819 | 15.327 |
| 4 | 48177 | 4055-4070 | 22.7 | 18.16 | 0.865 | 14.528 |
| 4 | 48200 | 4061 | 28.7 | 20.77 | 0.836 | 15.031 |
| 4 | 48200 | 4061 | 31.6 | 22.08 | 0.814 | 15.428 |
| 4 | 48201 | 4063 | 52.7 | 28.63 | 0.808 | 15.554 |
| 4 | 48211 | 4080 | 158.9 | 49.16 | 0.826 | 15.209 |
| 4 | 48213 | 4090 | 119.4 | 42.39 | 0.835 | 15.050 |
| 4 | 48215 | 4063 | 214.7 | 57.67 | 0.811 | 15.491 |
| 4 | 48216 | 4063 | 100.4 | 45.94 | 0.598 | 21.021 |
| 4 | 48220 | 4078 | 26.5 | 19.10 | 0.912 | 13.766 |
| 4 | 48237 | 4080 | 160.8 | 51.20 | 0.770 | 16.302 |
| 4 | 48238 | 4080 | 59.8 | 30.05 | 0.832 | 15.100 |
| 4 | 48240 | 4080 | 172.9 | 54.36 | 0.735 | 17.091 |
| 4 | 48240 | 4080 | 52.3 | 27.75 | 0.853 | 14.724 |
| 4 | 48249 | 4055-4070 | 107.4 | 40.25 | 0.833 | 15.084 |
| 4 | 48249 | 4055-4070 | 82.5 | 33.91 | 0.901 | 13.938 |
| 4 | 48251 | 4055-4070 | 60.7 | 29.57 | 0.872 | 14.405 |
| 4 | 48257 | 4055-4070 | 119.7 | 41.27 | 0.883 | 14.229 |
| 4 | 48257 | 4055-4070 | 61.3 | 30.91 | 0.806 | 15.586 |
| 4 | 48272 | 4070-4080 | 61.7 | 30.57 | 0.829 | 15.146 |
| 4 | 48277 | 4070-4080 | 22.1 | 19.79 | 0.709 | 17.721 |
| 4 | 48844 | 4068 | 39.5 | 23.54 | 0.895 | 14.029 |
| 4 | 48845 | 4068 | 172.4 | 50.15 | 0.861 | 14.588 |
| 4 | 48845 | unknown | 35.2 | 23.37 | 0.809 | 15.516 |
| 4 | 48848 | 4068 | 60.9 | 30.29 | 0.834 | 15.065 |
| 4 | 48851 | 4068 | 90.1 | 35.67 | 0.889 | 14.122 |
| 4 | 48852 | 4068 | 181.3 | 51.85 | 0.847 | 14.829 |
| 4 | 48852 | 4068 | 131 | 43.87 | 0.855 | 14.691 |
| 5 | 48080 | 4666 | 94.746 | 39.09 | 0.779 | 16.128 |
| 5 | 46977 | 4678 | 494.444 | 145.07 | 0.295 | 42.566 |
| 7 | 47739 | 7257 | 1243.51 | 141.79 | 0.777 | 16.168 |
| 7 | 47724 | unknown | 265.5 | 64.26 | 0.808 | 15.554 |
| 7 | 47715 | 7257 | 476.17 | 88.73 | 0.760 | 16.536 |
| 7 | 47716 | 7257 | 199.912 | 59.53 | 0.709 | 17.727 |
