# Supplementary material for: Increased Atmospheric SO2 Detected from Changes in Leaf Physiognomy across the Triassic–Jurassic Boundary Interval of East Greenland
Source: PLoS One. 2013 Apr 10;8(4):e60614. doi: 10.1371/journal.pone.0060614 (PMC3622679; doi:10.1371/journal.pone.0060614)
Supplement: Table S11 — All measured values for all fossil Pterophyllum leaves measured in the analysis. (DOC) [file pone.0060614.s011.doc]

Table S11: All measured values for all fossil *Pterophyllum* leaves measured in the analysis. Gray shading indicated that the value was an outlier (over twice the standard deviation of the mean value) and was not included in analyses. Samples are held in the Field Museum of Natural History, Chicago, Illinois, USA.

| Bed | Sample number | Height (cm) | Area (mm^2^) | Perimeter (mm) | Shape Factor | Compactness |
| --- | --- | --- | --- | --- | --- | --- |
| 1 | 46878 | 1378 | 156.9 | 107.27 | 0.171 | 73.339 |
| 1 | 46879 | 1388 | 182.8 | 60.07 | 0.637 | 19.740 |
| 1 | 46885 | 1380 | 44.3 | 25.79 | 0.837 | 15.014 |
| 1 | 46898 | 1358 | 57.4 | 29.73 | 0.817 | 15.398 |
| 1 | 46894 | 1383 | 103.5 | 55.63 | 0.420 | 29.900 |
| 1 | 46899 | 1358 | 36.8 | 27.41 | 0.615 | 20.416 |
| 1 | 46897 | 1388 | 43.8 | 25.05 | 0.876 | 14.327 |
| 1 | ? | 1383 | 47.5 | 27.58 | 0.785 | 16.014 |
| 1 | ? | 1383 | 28.6 | 20.59 | 0.847 | 14.823 |
| 1 | ? | 1383 | 30.4 | 21.79 | 0.803 | 15.619 |
| 1 | ? | 1383 | 54.8 | 30.45 | 0.743 | 16.920 |
| 1 | ? | 1383 | 29.6 | 21.91 | 0.775 | 16.218 |
| 1 | 46910 | 1363 | 121.2 | 45.72 | 0.728 | 17.247 |
| 1 | 46933 | 1373 | 84.4 | 42.12 | 0.598 | 21.020 |
| 1 | 46932 | 1390 | 57 | 31.69 | 0.713 | 17.619 |
| 1 | 46950 | 1388 | 95.2 | 39.70 | 0.759 | 16.556 |
| 1 | 46958 |  | 158.9 | 50.82 | 0.773 | 16.253 |
| 1 | 46965 | 1383 | 102.1 | 46.86 | 0.584 | 21.507 |
| 1 | no sample number | unknown | 45.7 | 29.31 | 0.668 | 18.798 |
| 1 | no sample number | unknown | 92 | 39.29 | 0.749 | 16.779 |
| 1.5 | 47133 | 2321 | 36.1 | 23.16 | 0.846 | 14.858 |
| 1.5 | 51069 | 1350 | 35.6 | 26.66 | 0.629 | 19.965 |
| 1.5 | 51073 | 1350 | 17.7 | 17.35 | 0.737 | 17.007 |
| 2 | 48376 | 3348.5 | 444.9 | 116.19 | 0.414 | 30.344 |
| 2 | 47120 | 3403 | 234.9 | 78.75 | 0.476 | 26.401 |
| 2 | 47121 | 3363 | 182.9 | 62.55 | 0.587 | 21.391 |
| 2 | 47111 | 3363 | 189.8 | 64.79 | 0.568 | 22.117 |
| 2 | 47111 | 3363 | 272.2 | 98.71 | 0.351 | 35.796 |
| 2 | 47081 | 3403 | 373.9 | 90.21 | 0.577 | 21.765 |
| 2 | 47081 | 3403 | 319.1 | 75.09 | 0.711 | 17.670 |
| 2 | 47085 | 3363 | 71 | 42.45 | 0.495 | 25.380 |
| 2 | 47084 | 3363 | 116.2 | 46.40 | 0.678 | 18.528 |
| 2 | 47086 | 3363 | 178.4 | 55.35 | 0.732 | 17.173 |
| 2 | 47087 | 3363 | 45.4 | 25.85 | 0.853 | 14.719 |
| 2 | 47022 | 3363 | 55.3 | 28.81 | 0.838 | 15.009 |
| 2 | 47066 | 3349 | 300.1 | 71.96 | 0.728 | 17.255 |
| 2 | 47048 | 3403 | 129.8 | 54.45 | 0.550 | 22.841 |
| 2 | 47052 | unknown | 99.4 | 42.26 | 0.699 | 17.967 |
| 2 | 47054 | 3425 | 56.7 | 29.32 | 0.829 | 15.162 |
| 2 | 47043 | 3348 | 215.3 | 97.50 | 0.285 | 44.154 |
| 2 | 47039 | 3363 | 180.7 | 60.84 | 0.613 | 20.484 |
| 2 | 47041 | 3398 | 107.9 | 41.31 | 0.795 | 15.816 |
| 2 | 47024 | 3366 | 396.4 | 96.73 | 0.532 | 23.604 |
| 2 | 48880 | 3363 | 62.1 | 31.25 | 0.799 | 15.726 |
| 2 | 47027 | unknown | 118.5 | 43.02 | 0.805 | 15.618 |
| 2 | 47023 | 3366 | 251.5 | 76.35 | 0.542 | 23.178 |
| 2 | 47022 | 3363 | 41 | 25.26 | 0.808 | 15.563 |
| 2 | 47016 | 3358 | 432.8 | 105.77 | 0.486 | 25.849 |
| 2 | 47016 | 3358 | 297.7 | 78.34 | 0.610 | 20.615 |
| 2 | 47014 | 3349 | 329.2 | 80.81 | 0.634 | 19.837 |
| 2 | 46999B | 3350 | 550.5 | 112.53 | 0.546 | 23.003 |
| 2 | 47089 | unknown | 121.7 | 43.53 | 0.807 | 15.570 |
| 3 | 48136 | 3765 | 73.7 | 32.92 | 0.855 | 14.705 |
| 4 | 47139 | 4072 | 37.9 | 24.10 | 0.820 | 15.325 |
| 4 | 47146 | 4061 | 136.2 | 45.55 | 0.824 | 15.233 |
| 4 | 47146 | unknown | 55.1 | 29.34 | 0.804 | 15.623 |
| 4 | 47148 | 4067 | 186 | 51.72 | 0.873 | 14.381 |
| 4 | 47148 | 4067 | 48.1 | 27.56 | 0.795 | 15.791 |
| 4 | 47149 | 4080 | 317.4 | 72.53 | 0.758 | 16.574 |
| 4 | 47149 | unknown | 40.5 | 25.23 | 0.799 | 15.717 |
| 4 | 47150 | 4067 | 210.3 | 55.59 | 0.855 | 14.694 |
| 4 | 47151 | 4063 | 166.6 | 50.55 | 0.819 | 15.338 |
| 4 | 47152 | 4068 | 183.1 | 51.46 | 0.868 | 14.463 |
| 4 | 47152 | 4068 | 129.2 | 45.89 | 0.771 | 16.299 |
| 4 | 47153 | 4072 | 160.1 | 49.44 | 0.823 | 15.267 |
| 4 | 47155 | 4074 | 196.2 | 57.35 | 0.749 | 16.764 |
| 4 | 47159 | 4074 | 179.1 | 52.81 | 0.807 | 15.572 |
| 4 | 47159 | 4074 | 60 | 33.01 | 0.692 | 18.161 |
| 4 | 47165 | 4060 | 103.5 | 39.21 | 0.846 | 14.854 |
| 4 | 47167 | 4060 | 104.7 | 41.01 | 0.782 | 16.063 |
| 4 | 47254 | 4098 | 116.2 | 43.00 | 0.789 | 15.912 |
| 4 | 47276 | 4070-4080 | 73.5 | 35.63 | 0.727 | 17.272 |
| 4 | 47277 | 4070-4080 | 129.7 | 45.68 | 0.781 | 16.088 |
| 4 | 47277 | 4070-4080 | 119.8 | 47.10 | 0.678 | 18.518 |
| 4 | 47278 | 4070-4080 | 183.7 | 54.97 | 0.764 | 16.449 |
| 4 | 47278 | 4070-4080 | 83.6 | 36.20 | 0.801 | 15.675 |
| 4 | 47292 | unknown | 102.8 | 39.41 | 0.831 | 15.108 |
| 4 | 47298 | unknown | 210.5 | 68.33 | 0.566 | 22.180 |
| 4 | 47300 | 4070-4075 | 99.3 | 39.96 | 0.781 | 16.081 |
| 4 | 47301 | 4070-4080 | 91.1 | 36.99 | 0.836 | 15.019 |
| 4 | 47304 | 4070-4080 | 108.6 | 40.02 | 0.852 | 14.748 |
| 4 | 47307 | 4070-4080 | 41 | 25.27 | 0.806 | 15.575 |
| 4 | 47307 | 4070-4080 | 39.5 | 23.97 | 0.863 | 14.546 |
| 4 | 47308 | 4070-4080 | 84.9 | 34.73 | 0.884 | 14.207 |
| 4 | 47309 | 4070-4080 | 63.6 | 30.49 | 0.859 | 14.617 |
| 4 | 47311 | 4070-4080 | 294.7 | 68.17 | 0.796 | 15.769 |
| 4 | 47312 | 4070-4080 | 207.8 | 57.82 | 0.781 | 16.088 |
| 4 | 47312 | 4070-4080 | 121.3 | 42.79 | 0.832 | 15.095 |
| 4 | 47313 | unknown | 63.2 | 31.81 | 0.784 | 16.011 |
| 4 | 47315 | 4070-4080 | 113.6 | 45.39 | 0.693 | 18.136 |
| 4 | 47316 | 4070-4080 | 84.1 | 35.43 | 0.841 | 14.926 |
| 4 | 47317 | 4070-4080 | 44.6 | 28.34 | 0.697 | 18.008 |
| 4 | 47321 | 4070-4080 | 236.9 | 59.33 | 0.845 | 14.859 |
| 4 | 47326 | 4080-4095 | 154.1 | 52.11 | 0.713 | 17.621 |
| 4 | 47329 | 4070-4080 | 99.8 | 39.06 | 0.822 | 15.287 |
| 4 | 47330 | 4070-4080 | 50.7 | 27.81 | 0.823 | 15.254 |
| 4 | 47330 | 4070-4080 | 71.2 | 32.18 | 0.864 | 14.544 |
| 4 | 47331 | 4070-4080 | 143.5 | 53.00 | 0.642 | 19.575 |
| 4 | 47334 | unknown | 66.4 | 31.53 | 0.839 | 14.972 |
| 4 | 47334 | 4070-4080 | 96.5 | 37.95 | 0.842 | 14.924 |
| 4 | 47335 | 4070-4080 | 66.4 | 31.59 | 0.836 | 15.029 |
| 4 | 47342 | 4070-4080 | 62.5 | 35.32 | 0.629 | 19.960 |
| 4 | 47345 | 4055-4070 | 47.8 | 27.18 | 0.813 | 15.455 |
| 4 | 47348 | 4055-4070 | 137.9 | 45.27 | 0.845 | 14.861 |
| 4 | 47349 | 4055-4070 | 168.8 | 49.43 | 0.868 | 14.475 |
| 4 | 47350 | unknown | 146.3 | 47.19 | 0.825 | 15.221 |
| 4 | 47353 | 4055-4070 | 96.8 | 37.70 | 0.855 | 14.683 |
| 4 | 47353 | 4055-4070 | 227.2 | 61.55 | 0.753 | 16.674 |
| 4 | 47354 | 4055-4070 | 106.8 | 47.14 | 0.604 | 20.807 |
| 4 | 47355 | 4055-4070 | 24.6 | 19.82 | 0.787 | 15.969 |
| 4 | 47356 | 4055-4070 | 89.3 | 35.05 | 0.913 | 13.757 |
| 4 | 47357 | 4055-4070 | 193.3 | 61.24 | 0.647 | 19.402 |
| 4 | 47366 | 4055-4070 | 211.9 | 57.99 | 0.791 | 15.870 |
| 4 | 47371 | 4055-4070 | 63.9 | 31.37 | 0.816 | 15.400 |
| 4 | 47374 | 4055-4070 | 48.9 | 27.44 | 0.816 | 15.398 |
| 4 | 47375 | 4055-4070 | 164.5 | 50.21 | 0.820 | 15.325 |
| 4 | 47377 | 4055-4070 | 155.6 | 49.05 | 0.812 | 15.462 |
| 4 | 47380 | 4075 | 64.5 | 30.21 | 0.888 | 14.150 |
| 4 | 47385 | 4080 | 153 | 48.64 | 0.812 | 15.463 |
| 4 | 47387 | 4080 | 62.3 | 31.77 | 0.775 | 16.201 |
| 4 | 47388 | 4075 | 67.6 | 31.87 | 0.836 | 15.025 |
| 4 | 47389 | 4075 | 203.7 | 55.62 | 0.827 | 15.187 |
| 4 | 47390 | 4080 | 62.6 | 30.73 | 0.833 | 15.085 |
| 4 | 47391 | 4075 | 260.7 | 74.64 | 0.588 | 21.370 |
| 4 | 47392 | 4075 | 51.1 | 27.30 | 0.861 | 14.585 |
| 4 | 47393 | 4065 | 47.6 | 26.73 | 0.837 | 15.010 |
| 4 | 47395 | 4080 | 260.7 | 74.64 | 0.588 | 21.370 |
| 4 | 47397 | 4090 | 163.3 | 49.85 | 0.825 | 15.218 |
| 4 | 47397 | 4090 | 192.4 | 56.27 | 0.763 | 16.457 |
| 4 | 47399 | 4080 | 63.6 | 30.99 | 0.832 | 15.100 |
| 4 | 47399 | 4080 | 204.6 | 57.09 | 0.788 | 15.930 |
| 4 | 47401 | 4070-4080 | 188.3 | 54.28 | 0.803 | 15.647 |
| 4 | 47416 | 4075 | 24.8 | 19.53 | 0.817 | 15.380 |
| 4 | 47416 | 4075 | 179.7 | 55.89 | 0.723 | 17.383 |
| 4 | 47417 | 4065 | 270 | 64.26 | 0.821 | 15.294 |
| 4 | 47418 | 4065 | 205.6 | 58.60 | 0.752 | 16.702 |
| 4 | 47419 | 4065 | 391.5 | 82.74 | 0.718 | 17.486 |
| 4 | 47419 | 4065 | 217 | 59.16 | 0.779 | 16.129 |
| 4 | 47419 | 4065 | 68.5 | 33.77 | 0.754 | 16.648 |
| 4 | 47421 | 4090 | 83.1 | 36.13 | 0.800 | 15.709 |
| 4 | 47424 | 4061 | 134.7 | 45.75 | 0.808 | 15.539 |
| 4 | 47429 | 4090 | 190.6 | 56.20 | 0.758 | 16.571 |
| 4 | 47434 | 4065 | 208.8 | 59.07 | 0.752 | 16.711 |
| 4 | 47436 | 4061 | 214.8 | 59.45 | 0.763 | 16.454 |
| 4 | 47441 | 4078 | 63.9 | 31.64 | 0.802 | 15.667 |
| 4 | 47447 | 4082 | 80.7 | 35.57 | 0.801 | 15.678 |
| 4 | 47449 | 4075 | 99.2 | 38.00 | 0.863 | 14.556 |
| 4 | 47449 | 4075 | 34.2 | 22.22 | 0.870 | 14.437 |
| 4 | 47452 | 4063 | 308.7 | 71.46 | 0.759 | 16.542 |
| 4 | 47456 | 4065 | 54.7 | 29.46 | 0.792 | 15.866 |
| 4 | 47456 | 4065 | 20 | 17.45 | 0.825 | 15.225 |
| 4 | 47460 | 4061 | 160.8 | 48.91 | 0.844 | 14.877 |
| 4 | 47464 | 4059 | 36.4 | 23.10 | 0.857 | 14.660 |
| 4 | 47465 | 4114 | 472.8 | 83.02 | 0.862 | 14.578 |
| 4 | 47466 | 4114 | 199.1 | 54.65 | 0.837 | 15.001 |
| 4 | 47468 | 4100 | 87.1 | 36.09 | 0.840 | 14.954 |
| 4 | 47469 | 4063 | 102 | 43.78 | 0.668 | 18.791 |
| 4 | 47470 | 4075 | 159.8 | 49.42 | 0.822 | 15.284 |
| 4 | 47475 | 4065 | 165.8 | 50.44 | 0.819 | 15.345 |
| 4 | 47476 | 4061 | 126.2 | 46.17 | 0.744 | 16.891 |
| 4 | 47479 | unknown | 83.6 | 35.99 | 0.811 | 15.494 |
| 4 | 47484 | 4055-4070 | 177.1 | 56.35 | 0.701 | 17.930 |
| 4 | 47487 | 4090 | 69.7 | 36.66 | 0.651 | 19.282 |
| 4 | 47494 | 4055-4070 | 201.5 | 56.98 | 0.780 | 16.113 |
| 4 | 47997 | 4070-4080 | 245.9 | 62.87 | 0.781 | 16.074 |
| 4 | 48145 | 4060 | 89.1 | 41.00 | 0.666 | 18.866 |
| 4 | 48145 | 4060 | 165.8 | 51.55 | 0.784 | 16.028 |
| 4 | 48153 | 4061 | 328.5 | 68.05 | 0.891 | 14.097 |
| 4 | 48154 | 4061 | 64.6 | 33.59 | 0.719 | 17.466 |
| 4 | 48157 | unknown | 157.3 | 47.16 | 0.888 | 14.139 |
| 4 | 48158 | 4061 | 86.7 | 38.22 | 0.745 | 16.849 |
| 4 | 48158 | 4061 | 60.6 | 30.74 | 0.805 | 15.593 |
| 4 | 48159 | unknown | 220.9 | 56.53 | 0.868 | 14.466 |
| 4 | 48160 | 4072 | 59.2 | 30.01 | 0.826 | 15.213 |
| 4 | 48161 | 4070 | 61.2 | 32.52 | 0.727 | 17.280 |
| 4 | 48162 | 4070 | 66.7 | 37.02 | 0.611 | 20.547 |
| 4 | 48164 | 4070 | 27.8 | 20.05 | 0.869 | 14.461 |
| 4 | 48165 | unknown | 60.6 | 29.82 | 0.856 | 14.674 |
| 4 | 48170 | 4070-4080 | 177.4 | 53.59 | 0.776 | 16.189 |
| 4 | 48175 | unknown | 61.7 | 30.65 | 0.825 | 15.226 |
| 4 | 48176 | unknown | 35.6 | 23.62 | 0.801 | 15.671 |
| 4 | 48182 | 4070 | 70.4 | 32.71 | 0.826 | 15.198 |
| 4 | 48186 | 4065 | 131.4 | 44.71 | 0.826 | 15.213 |
| 4 | 48188 | 4065 | 29.5 | 20.70 | 0.865 | 14.525 |
| 4 | 48190 | 4065 | 75.7 | 34.27 | 0.810 | 15.514 |
| 4 | 48196 | 4059 | 116.5 | 42.58 | 0.807 | 15.563 |
| 4 | 48197 | 4059 | 96.3 | 38.76 | 0.805 | 15.601 |
| 4 | 48198 | 4061 | 182.8 | 51.97 | 0.850 | 14.775 |
| 4 | 48199 | 4061 | 180.9 | 56.90 | 0.702 | 17.897 |
| 4 | 48202 | 4063 | 256.6 | 62.38 | 0.828 | 15.165 |
| 4 | 48203 | 4063 | 276.2 | 69.36 | 0.721 | 17.418 |
| 4 | 48204 | 4063 | 165.4 | 56.08 | 0.661 | 19.014 |
| 4 | 48205 | 4063 | 59 | 30.12 | 0.817 | 15.377 |
| 4 | 48205 | 4063 | 52.8 | 29.51 | 0.762 | 16.493 |
| 4 | 48206 | 4063 | 38.4 | 24.33 | 0.815 | 15.415 |
| 4 | 48209 | 4063 | 78.4 | 37.90 | 0.686 | 18.322 |
| 4 | 48210 | 4078 | 59.4 | 30.73 | 0.790 | 15.898 |
| 4 | 48212 | 4090 | 120.6 | 42.49 | 0.839 | 14.970 |
| 4 | 48213 | 4090 | 118 | 42.25 | 0.830 | 15.128 |
| 4 | 48225 | 4061 | 115.8 | 42.34 | 0.811 | 15.481 |
| 4 | 48226 | 4065 | 88.9 | 36.70 | 0.829 | 15.151 |
| 4 | 48241 | 4063 | 349.2 | 71.42 | 0.860 | 14.607 |
| 4 | 48242 | 4063 | 86.7 | 37.72 | 0.765 | 16.411 |
| 4 | 48243 | 4063 | 43.9 | 26.23 | 0.801 | 15.672 |
| 4 | 48244 | 4090 | 161.2 | 48.72 | 0.853 | 14.725 |
| 4 | 48245 | 4080 | 89.9 | 36.29 | 0.857 | 14.649 |
| 4 | 48253 | 4080 | 199.3 | 55.41 | 0.815 | 15.405 |
| 4 | 48254 | unknown | 141.9 | 48.38 | 0.761 | 16.495 |
| 4 | 48255 | unknown | 98.4 | 40.87 | 0.740 | 16.975 |
| 4 | 48259 | 4080 | 98 | 40.63 | 0.746 | 16.845 |
| 4 | 48260 | 4080 | 61 | 30.18 | 0.841 | 14.932 |
| 4 | 48260 | 4080 | 216.4 | 60.09 | 0.753 | 16.686 |
| 4 | 48261 | 4063 | 231.4 | 61.23 | 0.775 | 16.202 |
| 4 | 48263 | 4070-4080 | 56.1 | 28.42 | 0.872 | 14.397 |
| 4 | 48264 | 4070-4080 | 229.9 | 58.53 | 0.843 | 14.901 |
| 4 | 48270 | 4080-4095 | 51.5 | 33.46 | 0.578 | 21.739 |
| 4 | 48273 | 4070-4080 | 69.1 | 31.81 | 0.858 | 14.644 |
| 4 | 48274 | 4070-4080 | 126.2 | 41.92 | 0.902 | 13.925 |
| 4 | 48277 | 4070-4080 | 73.8 | 35.02 | 0.756 | 16.618 |
| 4 | 48842 | 4075 | 140.2 | 50.70 | 0.685 | 18.334 |
| 4 | 48843 | 4063 | 113.9 | 41.81 | 0.818 | 15.347 |
| 4 | 48844 | 4068 | 188.3 | 56.69 | 0.736 | 17.067 |
| 4 | 48845 | 4068 | 148 | 47.45 | 0.826 | 15.213 |
| 4 | 48880 | 4055-4070 | 140.2 | 46.87 | 0.802 | 15.669 |
| 4 | 48892 | 4055-4070 | 84.2 | 34.81 | 0.873 | 14.391 |
| 4 | 48892 | 4055-4070 | 160.5 | 48.83 | 0.845 | 14.856 |
| 4 | 48894 | 4060 | 203 | 55.01 | 0.843 | 14.907 |
| 4 | 48896 | 4060 | 115.4 | 41.43 | 0.844 | 14.874 |
| 4 | 48897 | 4060 | 210.2 | 58.23 | 0.779 | 16.131 |
| 4 | 48898 | 4070-4080 | 248.5 | 63.23 | 0.781 | 16.089 |
| 5 | 48013 |  | 1007.991 | 130.43 | 0.744 | 16.876 |
| 5 | 47392 | 4642 | 92.448 | 41.03 | 0.690 | 18.206 |
| 6 | 47782 | 6084 | 25.173 | 20.19 | 0.776 | 16.187 |
| 6 | 50026 | 6094 | 6.103 | 9.82 | 0.795 | 15.807 |
