# Supplementary material for: Increased Atmospheric SO2 Detected from Changes in Leaf Physiognomy across the Triassic–Jurassic Boundary Interval of East Greenland
Source: PLoS One. 2013 Apr 10;8(4):e60614. doi: 10.1371/journal.pone.0060614 (PMC3622679; doi:10.1371/journal.pone.0060614)
Supplement: Table S12 — Kruskal Wallis and Mann-Whitney U pair-wise comparisons for area in Anomozamites in the different beds in which leaves are present at Astartekløft, East Greenland. (DOC) [file pone.0060614.s012.doc]

Table S12: Kruskal Wallis and Mann-Whitney U pair-wise comparisons for area in *Anomozamites* in the different beds in which leaves are present at Astartekløft, East Greenland. Beds 1–5 are Triassic in age and beds 6–8 are Jurassic in age. Post-hoc pair-wise comparisons are based on Bonferroni-corrected Mann Whitney U test. Note that beds with less than 7 samples (See SI Appendix S2) many not provide accurate pair-wise comparisons.

| H = 19.17; p = 0.00388 | | | | | | | |
| --- | --- | --- | --- | --- | --- | --- | --- |
| 0 | 1 | 1.5 | 2 | 3 | 4 | 5 | 7 |
| 1 | 0 | 0.2268 | 0.729 | 0.2976 | 0.5508 | 0.06786 | 0.003495 |
| 1.5 |  | 0 | 0.08166 | 0.504 | 0.05411 | 0.5228 | 0.006362 |
| 2 |  |  | 0 | 0.2963 | 0.9706 | 0.04513 | 0.006928 |
| 3 |  |  |  | 0 | 0.4414 | 0.5403 | 0.2888 |
| 4 |  |  |  |  | 0 | 0.0637 | 0.0008308 |
| 5 |  |  |  |  |  | 0 | 0.817 |
| 7 |  |  |  |  |  |  | 0 |
