# Supplementary material for: Increased Atmospheric SO2 Detected from Changes in Leaf Physiognomy across the Triassic–Jurassic Boundary Interval of East Greenland
Source: PLoS One. 2013 Apr 10;8(4):e60614. doi: 10.1371/journal.pone.0060614 (PMC3622679; doi:10.1371/journal.pone.0060614)
Supplement: Table S15 — Kruskal Wallis and Mann-Whitney U pair-wise comparisons for compactness in Anomozamites in the different beds in which leaves are present at Astartekløft, East Greenland. (DOC) [file pone.0060614.s015.doc]

Table S15: Kruskal Wallis and Mann-Whitney U pair-wise comparisons for compactness in *Anomozamites* in the different beds in which leaves are present at Astartekløft, East Greenland. Beds 1–5 are Triassic in age and beds 6–8 are Jurassic in age. Post-hoc pair-wise comparisons are based on Bonferroni-corrected Mann Whitney U test. Note that beds with less than 7 samples (See SI Appendix S2) many not provide accurate pair-wise comparisons.

| H = 48.81; p = 8.148^e-9^ | | | | | | | |
| --- | --- | --- | --- | --- | --- | --- | --- |
| 0 | 1 | 1.5 | 2 | 3 | 4 | 5 | 7 |
| 1 | 0 | 0.0001922 | 0.9749 | 0.1325 | 0.02013 | 0.09548 | 0.1517 |
| 1.5 |  | 0 | 0.0009511 | 0.6885 | 3.278^e-8^ | 0.9273 | 0.01804 |
| 2 |  |  | 0 | 0.1637 | 0.03542 | 0.2888 | 0.4875 |
| 3 |  |  |  | 0 | 0.09033 | 0.5403 | 0.2888 |
| 4 |  |  |  |  | 0 | 0.02553 | 0.004119 |
| 5 |  |  |  |  |  | 0 | 0.817 |
| 7 |  |  |  |  |  |  | 0 |
