# Supplementary material for: Increased Atmospheric SO2 Detected from Changes in Leaf Physiognomy across the Triassic–Jurassic Boundary Interval of East Greenland
Source: PLoS One. 2013 Apr 10;8(4):e60614. doi: 10.1371/journal.pone.0060614 (PMC3622679; doi:10.1371/journal.pone.0060614)
Supplement: Table S16 — Kruskal Wallis and Mann-Whitney U pair-wise comparisons for area in Pterophyllum in the different beds in which leaves are present at Astartekløft, East Greenland. (DOC) [file pone.0060614.s016.doc]

Table S16: Kruskal Wallis and Mann-Whitney U pair-wise comparisons for area in *Pterophyllum* in the different beds in which leaves are present at Astartekløft, East Greenland. Beds 1–5 are Triassic in age and beds 6–8 are Jurassic in age. Post-hoc pair-wise comparisons are based on Bonferroni-corrected Mann Whitney U test. Note that beds with less than 7 samples (See SI Appendix S2) many not provide accurate pair-wise comparisons.

| H = 32.15; p = 1.53^e-5^ | | | | | | | |
| --- | --- | --- | --- | --- | --- | --- | --- |
| 0 | 1 | 1.5 | 2 | 3 | 4 | 5 | 6 |
| 1 | 0 | 0.03937 | 0.0001872 | 0.7842 | 0.0006442 | 0.5228 | 0.02747 |
| 1.5 |  | 0 | 0.005833 | 0.3711 | 0.005184 | 0.3711 | 0.3865 |
| 2 |  |  | 0 | 0.404 | 0.01099 | 0.404 | 0.02292 |
| 3 |  |  |  | 0 | 0.5357 | 1 | 0.5403 |
| 4 |  |  |  |  | 0 | 0.793 | 0.01752 |
| 5 |  |  |  |  |  | 0 | 0.5403 |
