# Supplementary material for: Increased Atmospheric SO2 Detected from Changes in Leaf Physiognomy across the Triassic–Jurassic Boundary Interval of East Greenland
Source: PLoS One. 2013 Apr 10;8(4):e60614. doi: 10.1371/journal.pone.0060614 (PMC3622679; doi:10.1371/journal.pone.0060614)
Supplement: Table S19 — Kruskal Wallis and Mann-Whitney U pair-wise comparisons for compactness in Pterophyllum in the different beds in which leaves are present at Astartekløft, East Greenland. (DOC) [file pone.0060614.s019.doc]

Table S19: Kruskal Wallis and Mann-Whitney U pair-wise comparisons for compactness in *Pterophyllum* in the different beds in which leaves are present at Astartekløft, East Greenland. Beds 1–5 are Triassic in age and beds 6–8 are Jurassic in age. Post-hoc pair-wise comparisons are based on Bonferroni-corrected Mann Whitney U test. Note that beds with less than 7 samples (See SI Appendix S2) many not provide accurate pair-wise comparisons.

| H = 38.07; p = 1.09^e-6^ | | | | | | | |
| --- | --- | --- | --- | --- | --- | --- | --- |
| 0 | 1 | 1.5 | 2 | 3 | 4 | 5 | 6 |
| 1 | 0 | 0.9599 | 0.05317 | 0.1709 | 0.002005 | 0.5228 | 0.4128 |
| 1.5 |  | 0 | 0.267 | 0.3711 | 0.2755 | 1 | 0.7728 |
| 2 |  |  | 0 | 0.1085 | 2.172^e-7^ | 0.9488 | 0.3967 |
| 3 |  |  |  | 0 | 0.2274 | 1 | 0.5403 |
| 4 |  |  |  |  | 0 | 0.1178 | 0.3705 |
| 5 |  |  |  |  |  | 0 | 0.5403 |
