# Supplementary material for: Increased Atmospheric SO2 Detected from Changes in Leaf Physiognomy across the Triassic–Jurassic Boundary Interval of East Greenland
Source: PLoS One. 2013 Apr 10;8(4):e60614. doi: 10.1371/journal.pone.0060614 (PMC3622679; doi:10.1371/journal.pone.0060614)
Supplement: Table S21 — Kruskal Wallis and Mann-Whitney U pair-wise comparisons for perimeter in Elatocladus in the different beds in which leaves are present at Astartekløft, East Greenland. (DOC) [file pone.0060614.s021.doc]

Table S21: Kruskal Wallis and Mann-Whitney U pair-wise comparisons for perimeter in *Elatocladus* in the different beds in which leaves are present at Astartekløft, East Greenland. Beds 1–5 are Triassic in age and beds 6–8 are Jurassic in age. Post-hoc pair-wise comparisons are based on Bonferroni-corrected Mann Whitney U test. Note that beds with less than 7 samples (See SI Appendix S2) many not provide accurate pair-wise comparisons.

| H = 23.98; p = 2.536^e-5^ | | | | |
| --- | --- | --- | --- | --- |
| Bed | 1.5 | 2 | 4 | 5 |
| 1.5 | 0 | 0.04436 | 0.002694 | 0.07575 |
| 2 |  | 0 | 0.0005684 | 0.001166 |
| 4 |  |  | 0 | 0.01219 |
