# Supplementary material for: Increased Atmospheric SO2 Detected from Changes in Leaf Physiognomy across the Triassic–Jurassic Boundary Interval of East Greenland
Source: PLoS One. 2013 Apr 10;8(4):e60614. doi: 10.1371/journal.pone.0060614 (PMC3622679; doi:10.1371/journal.pone.0060614)
Supplement: Table S24 — Kruskal Wallis and Mann-Whitney U pair-wise comparisons for area in Podozamites in the different beds in which leaves are present at Astartekløft, East Greenland. (DOC) [file pone.0060614.s024.doc]

Table S24: Kruskal Wallis and Mann-Whitney U pair-wise comparisons for area in *Podozamites* in the different beds in which leaves are present at Astartekløft, East Greenland. Beds 1–5 are Triassic in age and beds 6–8 are Jurassic in age. Post-hoc pair-wise comparisons are based on Bonferroni-corrected Mann Whitney U test. Note that beds with less than 7 samples (See SI Appendix S2) many not provide accurate pair-wise comparisons.

| H = 98.38; p= 1.16^e-19^ | | | | | | |
| --- | --- | --- | --- | --- | --- | --- |
| Bed | 1 | 2 | 3 | 4 | 5 | 8 |
| 1 | 0 | 0.07683 | 0.0001023 | 0.3909 | 9.478^e-07^ | 0.0001047 |
| 2 |  | 0 | 0.1972 | 0.03948 | 2.438^e-15^ | 0.0002737 |
| 3 |  |  | 0 | 0.03948 | 2.438^e-15^ | 0.002931 |
| 4 |  |  |  | 0 | 0.0007183 | 0.001476 |
| 5 |  |  |  |  | 0 | 0.02104 |
| 8 |  |  |  |  |  | 0 |
