# Supplementary material for: Increased Atmospheric SO2 Detected from Changes in Leaf Physiognomy across the Triassic–Jurassic Boundary Interval of East Greenland
Source: PLoS One. 2013 Apr 10;8(4):e60614. doi: 10.1371/journal.pone.0060614 (PMC3622679; doi:10.1371/journal.pone.0060614)
Supplement: Table S27 — Kruskal Wallis and Mann-Whitney U pair-wise comparisons for compactness in Podozamites in the different beds in which leaves are present at Astartekløft, East Greenland. (DOC) [file pone.0060614.s027.doc]

Table S27: Kruskal Wallis and Mann-Whitney U pair-wise comparisons for compactness in *Podozamites* in the different beds in which leaves are present at Astartekløft, East Greenland. Beds 1–5 are Triassic in age and beds 6–8 are Jurassic in age. Post-hoc pair-wise comparisons are based on Bonferroni-corrected Mann Whitney U test. Note that beds with less than 7 samples (See SI Appendix S2) many not provide accurate pair-wise comparisons.

| Table S27: Kruskal-Wallis analysis of compactness measurements for *Podozamites*. | | | | | | |
| --- | --- | --- | --- | --- | --- | --- |
| H = 168.9; p= 1.252^e-34^ | | | | | | |
| Bed | 1 | 2 | 3 | 4 | 5 | 8 |
| 1 | 0 | 0.03453 | 1.375^e-7^ | 0.1087 | 1.186^e-9^ | 0.0002514 |
| 2 |  | 0 | 1.593^e-5^ | 0.7686 | 3.546^e-13^ | 0.008585 |
| 3 |  |  | 0 | 0.0024 | 2.586^e-25^ | 0.6825 |
| 4 |  |  |  | 0 | 1.033^e-5^ | 0.02417 |
| 5 |  |  |  |  | 0 | 1.676^e-7^ |
| 8 |  |  |  |  |  | 0 |
