# Supplementary material for: Increased Atmospheric SO2 Detected from Changes in Leaf Physiognomy across the Triassic–Jurassic Boundary Interval of East Greenland
Source: PLoS One. 2013 Apr 10;8(4):e60614. doi: 10.1371/journal.pone.0060614 (PMC3622679; doi:10.1371/journal.pone.0060614)
Supplement: Table S29 — Kruskal Wallis and Mann-Whitney U pair-wise comparisons for perimeter in Baiera in the different beds in which leaves are present at Astartekløft, East Greenland. (DOC) [file pone.0060614.s029.doc]

Table S29: Kruskal Wallis and Mann-Whitney U pair-wise comparisons for perimeter in *Baiera* in the different beds in which leaves are present at Astartekløft, East Greenland. Beds 1–5 are Triassic in age and beds 6–8 are Jurassic in age. Post-hoc pair-wise comparisons are based on Bonferroni-corrected Mann Whitney U test. Note that beds with less than 7 samples (See SI Appendix S2) many not provide accurate pair-wise comparisons.

| H = 5.128; p = 0.07698 | | | |
| --- | --- | --- | --- |
| Bed | 1 | 1.5 | 3 |
| 1 | 0 | 0.9214 | 0.03122 |
| 1.5 |  | 0 | 0.2472 |
